# Crizotinib- or Ceritinib-Conjugated Platinum(IV) Prodrugs As Potent Multiaction Agents Inducing Antiproliferative Effects in 2D and 3D Cancer Cell Models

**DOI:** 10.1021/acs.jmedchem.5c01858

**Published:** 2025-11-10

**Authors:** Sofia Sharkawy, Sourav Acharya, Hana Kostrhunová, Moumita Maji, Lenka Marková, Vojtěch Novohradský, Dan Gibson, Viktor Brabec

**Affiliations:** † Faculty of Science, Department of Biochemistry, Masaryk University, CZ-62500 Brno, Czech Republic; ‡ Czech Academy of Sciences, Institute of Biophysics, Kralovopolska 135, CZ-61200 Brno, Czech Republic; § Institute for Drug Research, School of Pharmacy, 26742 The Hebrew University of Jerusalem, Jerusalem-9112102, Israel; ∥ Department of Biophysics, Faculty of Science, Palacky University, Slechtitelu 27, 783 71 Olomouc, Czech Republic

## Abstract

Novel Pt­(IV) complexes conjugated with the kinase inhibitors crizotinib or ceritinib were synthesized and assessed for anticancer activity. Cisplatin-derived derivatives bearing phenylbutyrate and either crizotinib (complex **3**) or ceritinib (complex **7**) exhibited the greatest efficacy and selectivity against cancer cells while sparing noncancerous counterparts. Both compounds maintained activity in three-dimensional spheroid models, where they reduced viability, inhibited migration, and suppressed invasive outgrowth. Cellular accumulation studies confirmed efficient uptake of **3** and **7**. Mechanistic investigations revealed that crizotinib-containing complexes induced G2/M arrest, whereas ceritinib analogs, particularly **7**, caused S-phase arrest and DNA damage responses. Moreover, both agents triggered apoptosis and hallmarks of immunogenic cell death, including calreticulin exposure, ATP and HMGB1 release, and enhanced phagocytosis by macrophages. These findings highlight complexes **3** and **7** as promising multifunctional candidates that combine cytotoxic, anti-invasive, and immune-activating properties, supporting Pt­(IV)–kinase inhibitor conjugates as a potential strategy for targeted cancer chemotherapy.

## Graphical abstract

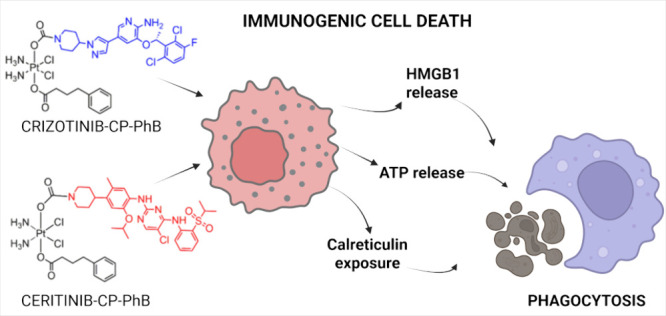

## Introduction

Platinum-based chemotherapeutic agents, such as cisplatin, carboplatin, and oxaliplatin, are widely used in clinical oncology and serve as first-line treatments for various types of cancer.^1^ These compounds are reactive square-planar Pt­(II) complexes (Figure 1A) that exert cytotoxic effects primarily by binding to nuclear DNA, thereby disrupting replication and transcription processes and inducing apoptosis.^2–4^ However, their lack of selectivity for cancer cells often results in dose-limiting toxicities, and tumor resistance to these agents remains a significant clinical challenge.^5^

**Figure 1:**
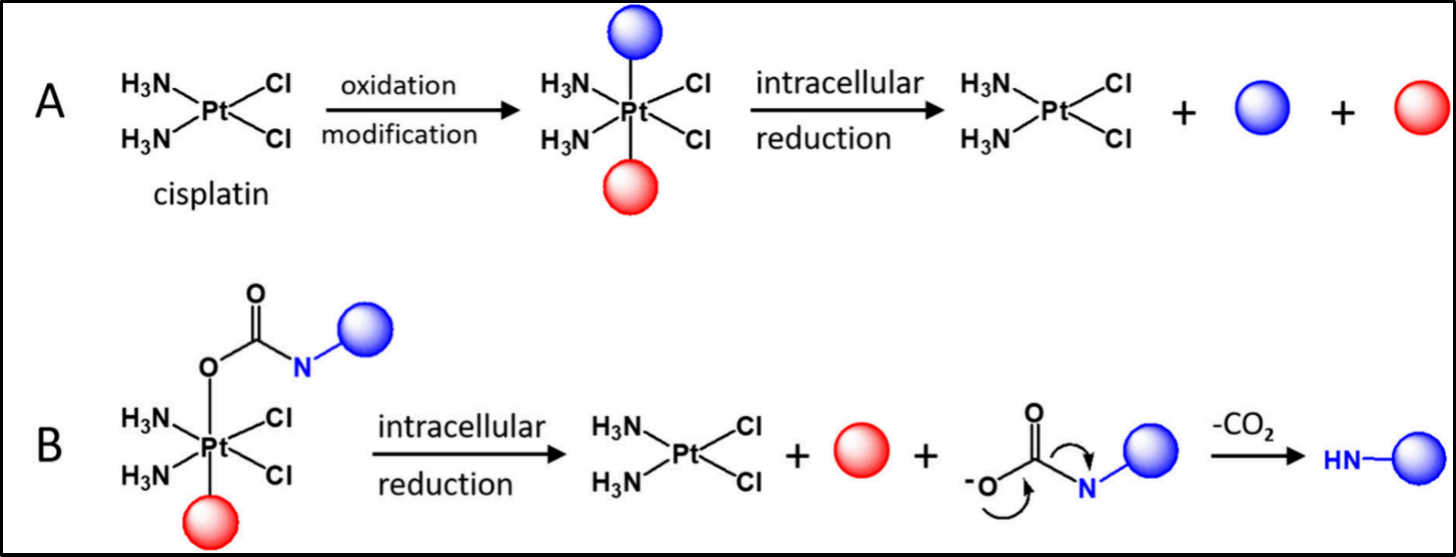
(A) reactive cisplatin is oxidized and modified to yield an inert 6-coordinate multiaction prodrug that is reduced in the cells to release cisplatin and the axial ligands. (B) A bioactive molecule that is conjugated to the Pt­(IV) through its secondary amine via a carbamate linkage is released in its active form following the reduction of the Pt­(IV) and decarboxylation of the released axial ligand.

To address these limitations, considerable efforts have been directed toward the development of platinum­(IV)-based prodrugs.^6–8^ Pt­(IV) complexes are kinetically inert, six-coordinate octahedral species derived from the oxidative addition of Pt­(II) compounds. These prodrugs undergo intracellular reduction, releasing the active Pt­(II) species along with two axial ligands (Figure 1A). When these axial ligands possess inherent biological activity, Pt­(IV) complexes function as multiaction agents, offering synergistic therapeutic effects while potentially mitigating toxicity and overcoming resistance.^8^

In clinical practice, platinum drugs are frequently administered in combination with other therapeutic agents.^9,10^ In this context, we consider it important to address some advantages and limitations of using antitumor Pt­(IV) complexes bearing bioactive axial ligands, as compared to administering a mixture of a parent Pt­(II) or Pt­(IV) complex with a separate bioactive molecule, thus providing a clearer context for the rationale behind the design of our Pt­(IV) complexes (for more details see, e.g., refs 11–16 ). The potential benefits of incorporating bioactive ligands directly into Pt­(IV) complexes, include: (i) Synergistic activity due to the corelease of both active components within the same cellular environment; (ii) improved pharmacokinetics and tumor targeting through modulation of lipophilicity and uptake; (iii) reduced systemic toxicity and better tolerability due to the prodrug nature of Pt­(IV) complexes; ability to overcome resistance mechanisms common to traditional Pt­(II) drugs; (iv) controlled and localized release of both the platinum core and the bioactive ligand; (v) multifunctional/“multi-action” mechanisms in a single entity. We also outline potential disadvantages and limitations: (i) The need for precise control of release kinetics; (ii) risk that ligand activity may be compromised upon conjugation; (iii) potential toxicity of the released ligand; (iv) synthetic complexity and regulatory challenges; (v) not every conjugate succeeds: axial ligands must not prevent Pt reduction/activation, and conjugation can alter cell uptake or potency; many promising Pt­(IV) conjugates still fail in translation. To illustrate the clinical relevance, we highlight that coadministration of a Pt complex and a bioactive ligand as separate agents often results in differing pharmacokinetics, lack of colocalization, and suboptimal therapeutic ratios, all of which may be addressed by rational Pt­(IV) prodrug design.

Interestingly, platinum drugs are often paired with pemetrexed^17,18^ or combined with ALK (anaplastic lymphoma kinase) inhibitors such as crizotinib and ceritinib for the treatment of ALK-rearranged NSCLC.^19^ ALK is a receptor tyrosine kinase involved in cellular signaling pathways that promote proliferation and survival; its dysregulation contributes to oncogenesis.^20^ ALK inhibitors block the kinase activity of the protein, thereby inhibiting downstream signaling cascades.^21^

Given the clinical success of combining ALK inhibitors with platinum-based chemotherapy, we aimed to develop Pt­(IV) prodrugs that integrate cisplatin or oxaliplatin with either crizotinib or ceritinib. These multimodal agents are designed to release both the DNA-damaging Pt­(II) moiety and the ALK inhibitor upon intracellular reduction, thereby targeting complementary cancer pathways. Notably, crizotinib and ceritinib are also recognized as inducers of immunogenic cell death (ICD), which can stimulate antitumor immune responses.^22^

While numerous multiaction Pt­(IV) prodrugs have been reported, most rely on the presence of a carboxylate functional group on the axial ligand to facilitate conjugation and subsequent release of the active form upon reduction. However, neither crizotinib nor ceritinib contains a carboxylate group, necessitating an alternative strategy. We therefore employed self-immolative linkers to conjugate these agents to the Pt­(IV) scaffold via carbamate bonds formed at their secondary amine sites, ensuring release of the intact, active drug (Figure 1B).^23^ Moreover, we incorporated another bioactive ligand, 4-phenylbutyrate, into the second axial position to form a “triple action” prodrug, as 4-phenylbutyrate is known to inhibit histone deacetylase (HDAC).^11^

Herein, we report the design, synthesis, characterization, and biological evaluation of a new class of multiaction Pt­(IV) prodrugs incorporating cisplatin or oxaliplatin and ALK inhibitors. To our knowledge, this study presents the first example of a Pt­(IV) prodrug conjugated to an ALK inhibitor, representing a novel approach to multiaction cancer therapies.

## Results and Discussion

### Synthesis

The Pt­(IV) prodrugs of crizotinib and ceritinib that were prepared for this study are depicted in Figure 2. The synthetic procedure illustrated in Scheme S1 is similar to that described previously.^23^ Briefly, the axial OH group of Pt­(IV) precursors (Scheme S1A–C) were activated with *N*,*N*′-disuccinylcarbonate (DSC) (Scheme S1D–F) and then reacted with crizotinib (**1**) or ceritinib (**5**) in DMF forming the multiaction Pt­(IV) prodrugs via a stable carbamate linkage (**2**–**4** and **6**–**8**). All the final complexes were purified by preparative high-performance liquid chromatography (HPLC) with isolated yields ranging from 40% to 56%. The complexes were characterized by HPLC, NMR, electrospray ionization mass spectrometry (ESI MS), and elemental analysis (Figures S1–S25).

**Figure 2:**
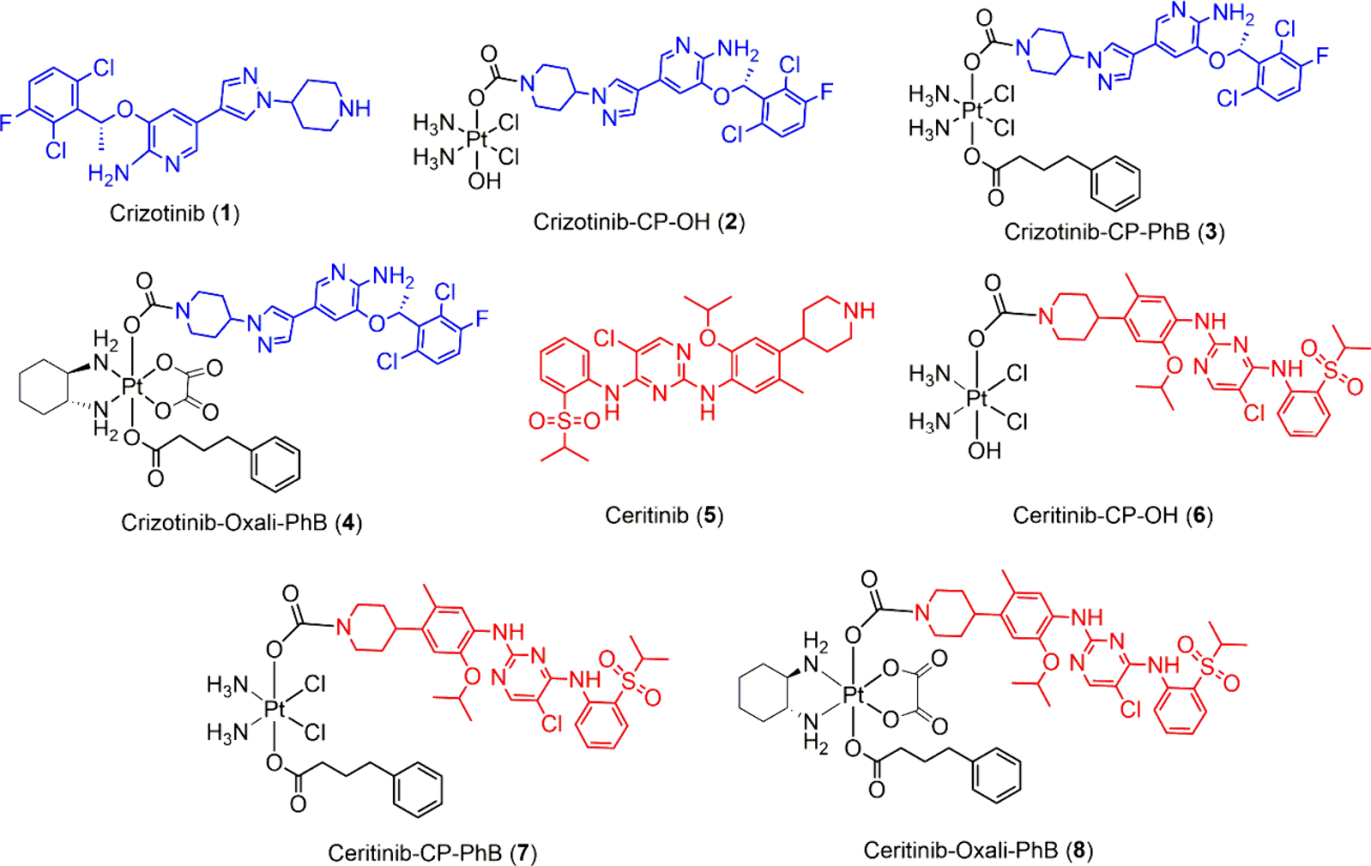
Chemical structures of the studied compounds.

### Stability in Cell Culture Medium

Two conditions must be met for Pt­(IV) complexes to serve as true prodrugs: they must remain stable in biological fluids and should be activated by reduction within the cancer cells to release the bioactive components in their active form, thereby facilitating the rapid onset of cytotoxic effects. Therefore, we evaluated the stability of the Pt­(IV) complexes of crizotinib (**2**–**4**) and ceritinib (**6**–**8**) in 10% DMSO in RPMI cell culture medium containing serum at 37 °C. The reduction in the area of the HPLC peak of the compounds was monitored as a function of time, and their half-lives were calculated (Table 1 and Figures S26,S27). All the compounds were stable, with half-lives ranging from 42 h to greater than 72 h. The oxaliplatin derivatives **4** and **8** are relatively more stable than the cisplatin derivatives with the identical axial ligands, with more than 80% and 65% respectively, of intact Pt­(IV) prodrugs still present after 3 days in the media (Figure S27). We also monitored the stability of the complexes in 10% DMSO in PBS at 37 °C following the reviewer’s suggestion. The results show that all the complexes are stable and have half-lives exceeding 72 h (Figure S28), ruling out premature activation of the prodrugs.

**Table 1:** Stability and Reduction of Complexes **2**–**4** and **6**–**8**

complexes	stability half-life (*t*_1/2_) h	reduction half-life (*t*_1/2_) h
**2**	75	2.7
**3**	42	7.7
**4**	>75	11.5
**6**	48	2.2
**7**	37	ND^a^
**8**	>75	12.5

a ND: not determined. Due to the poor solubility of complex **7** in phosphate buffer, it precipitates with time; thus, calculating *t*_1/2_ (half-life) is inconclusive. However, the reduction plot in Figure S32 clearly shows the release of free ceritinib.

In connection with the discussion focused on the stability of the investigated Pt complexes in biologically relevant environments, it is necessary to mention that while many cisplatin-derived Pt­(IV) complexes indeed undergo rapid biotransformation, reduction, or protein binding in the bloodstream, the extent of this instability varies significantly with the chemical structure. For example, a Pt­(IV) prodrug capable of noncovalent interaction with human serum albumin demonstrated significantly enhanced stability in whole blood compared to earlier, less modified analogues.^24^ Nonetheless, as reported in the literature,^25^ premature activation in blood remains a challenge for many Pt­(IV) prodrugs. Importantly, Pt­(IV) complexes with carefully selected ligand environments, particularly those that are more hydrophilic or sterically hindered, can exhibit increased kinetic inertness in blood.^26^ In our study, complexes **3** and **7** include phenylbutyrate axial ligands, which are known to enhance lipophilicity and potentially facilitate cellular uptake.^27^ However, their behavior in blood has not yet been evaluated experimentally. Thus, it should be noted that for the further preclinical or clinical development of the investigated Pt­(IV) compounds, it will be essential to evaluate their stability in blood models. Should stability prove insufficient, structural modifications may be required to enhance blood stability without compromising activity.

### Reduction of the Compounds

The reduction of Pt­(IV) complexes was performed by reacting with 10 equiv of l-ascorbic acid in 10% DMSO in 100 mM phosphate buffer (pH 7.4) at 37 °C, and was monitored by reverse-phase analytical HPLC. The half-lives for the reduction were calculated from the decreasing peak areas as a function of time. Importantly, we observed that following reduction, free crizotinib was released from compounds **2**–**4** (Figure 3 and Figures S29 and S30) and free ceritinib from compounds **6**–**8** (Figures S31–S33). The half-lives for the reduction of compounds **2**–**4** are reported in Table 1. The Pt­(IV) derivatives of cisplatin are reduced faster than the corresponding oxaliplatin derivatives with identical axial ligands and the monofuctionalized Pt­(IV) derivatives of cisplatin (**2** and **6**) are reduced faster than the bifunctionalized complexes (**3** and **7**) probably due to reduction of **2** and **6** by an inner sphere interaction of the ascorbate and the axial OH.^28^ Taken together, all of the complexes are very stable and are readily reduced.

**Figure 3:**
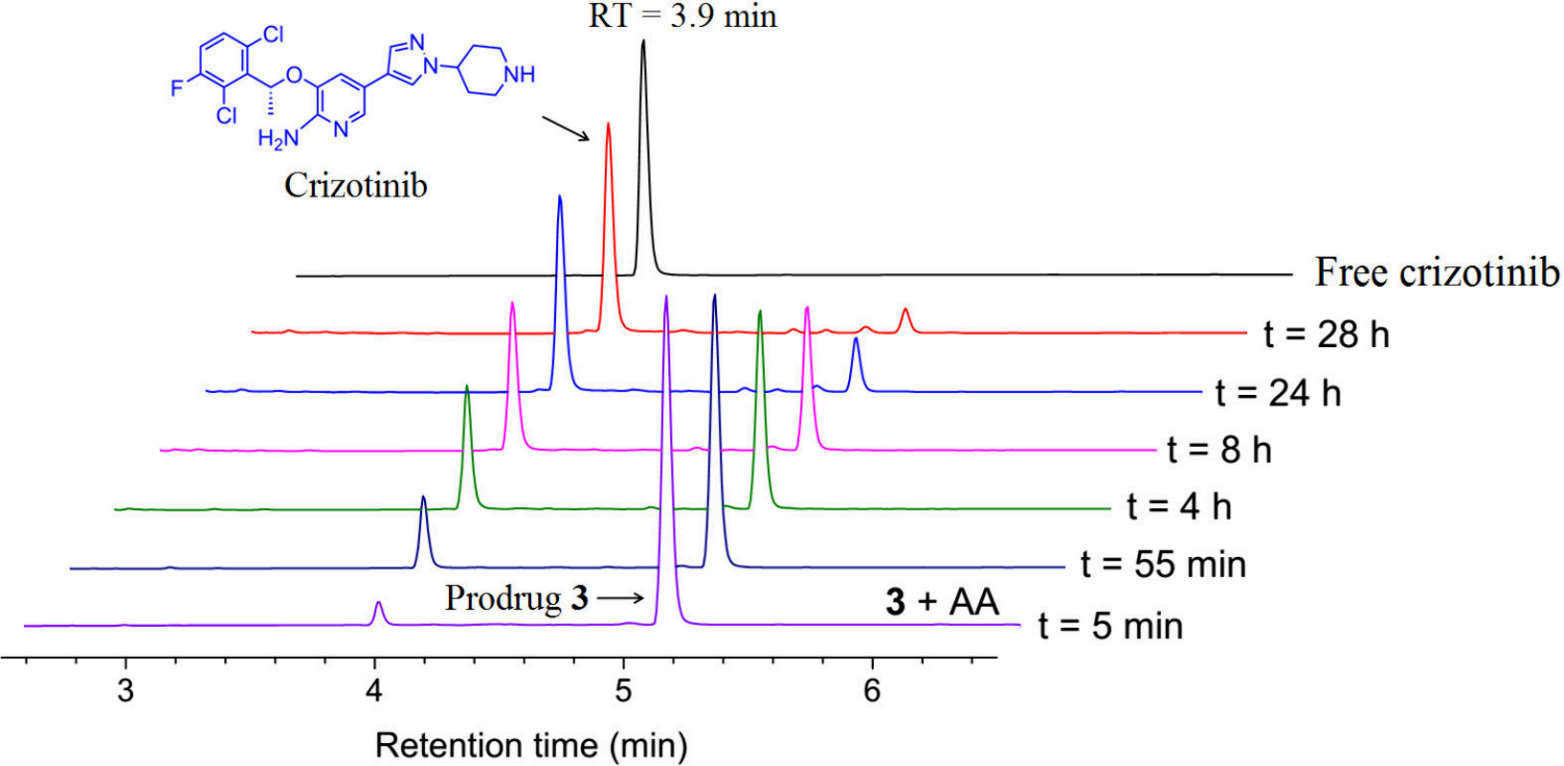
Reduction of **3** in the presence of 10 equiv of ascorbic acid taken at different time intervals in 0.1 M phosphate buffer at pH 7.4 at 37 °C. RT = 3.9 min shows the release of free crizotinib.

### Cytotoxic/Antiproliferative Activity

A panel of six cancer cell lines was used to evaluate the cytotoxic potential of the investigated compounds. IC_50_ values (IC_50_ = concentration of the agent inhibiting cell growth by 50%, relative to the nontreated control) determined using the 3-[4,5-dimethylthiazol-2-yl]-2,5 diphenyl tetrazolium bromide (MTT) assay after a 72 h treatment, are shown in Table 2. For comparative purposes, mixtures **MIX3**, **MIX4**, **MIX7**, and **MIX8**, containing the constituents of **3**, **4**, **7**, and **8**, respectively, in a 1:1:1 stoichiometric ratio (cisplatin/oxaliplatin:crizotinib/ceritinib:phenylbutyrate) were prepared and analyzed. The ligands alone (**1** and **5**) were effective against all cancer cell lines at low micromolar concentrations (and in some cases, submicromolar concentrations). These results are in accordance with published data.^29^ Pt­(IV) compounds monofunctionalized with either crizotinib or ceritinib (compounds **2** and **6**) showed slightly improved cytotoxicity compared with the ligands (and cisplatin). Further functionalization with phenylbutyrate resulted in even more potent agents, with IC_50_ values in the ranges of 0.44–0.57 μM and 0.07–0.35 μM for crizotinib and ceritinib, respectively. **7**, followed by **6** and **3**, were the most potent agents within this panel of cancer cell lines. The IC_50_ values for Pt­(IV) complexes (**2**–**4** and **6**–**8**) were determined using the molecular weights of the complexes, including associated trifluoroacetic acid and solvent molecules.

**Table 2:** Cytotoxic/Antiproliferative Activity in Human Cancer Cell Lines (IC_50_ Values (μM))^a^ ^,^ ^b^

	A549	NCI-H2228	U87-MG	HCT116	RD	MDA-MB-231
**1**	1.0 ± 0.2	0.82 ± 0.07	3.9 ± 0.5	1.9 ± 0.5	1.4 ± 0.4	1.2 ± 0.3
**2**	0.8 ± 0.1	0.74 ± 0.08	2.0 ± 0.7	2.1 ± 0.5	2.0 ± 0.4	1.9 ± 0.6
**3**	0.48 ± 0.06	0.45 ± 0.09	0.44 ± 0.05	0.57 ± 0.09	0.54 ± 0.07	0.50 ± 0.07
**4**	1.5 ± 0.4	1.3 ± 0.4	1.8 ± 0.4	1.0 ± 0.2	1.6 ± 0.2	1.5 ± 0.5
**5**	0.9 ± 0.2	0.51 ± 0.04	1.1 ± 1	0.9 ± 0.2	1.5 ± 0.3	1.4 ± 0.9
**6**	0.35 ± 0.06	0.21 ± 0.06	0.8 ± 0.2	0.7 ± 0.3	0.6 ± 0.2	1.1 ± 0.3
**7**	0.23 ± 0.08	0.07 ± 0.01	0.26 ± 0.09	0.19 ± 0.06	0.35 ± 0.03	0.22 ± 0.08
**8**	1.3 ± 0.2	0.8 ± 0.3	1.1 ± 0. 4	0.88 ± 0.06	2.2 ± 0.4	1.5 ± 0.4
**cisPt**	4.3 ± 0.2	2.4 ± 0.7	3.0 ± 0.2	8.8 ± 0.5	7.3 ± 0.2	27 ± 4
**OxPt**	1.6 ± 0.6	3.8 ± 0.4	ND^c^	1.3 ± 0.1	4.3 ± 0.5	6.2 ± 0.9
**MIX3**	1.1 ± 0.4	0.71 ± 0.07	1.4 ± 0.4	1.6 ± 0.6	1.1 ± 0.2	1.3 ± 0.2
**MIX4**	0.6 ± 0.1	0.7 ± 0.2	ND	ND	ND	ND
**MIX7**	0.9 ± 0.3	0.44 ± 0.04	0.62 ± 0.08	0.59 ± 0.04	0.61 ± 0.06	0.9 ± 0.1
**MIX8**	2.0 ± 0.3	3.1 ± 0.7	ND	ND	ND	ND
**MIX3**/**3** ^d^	2.3	1.6	3.2	2.8	2.0	2.6
**MIX7**/**7** ^e^	3.9	6.3	2.4	3.1	1.7	4.1

a Cell viability was determined with the MTT assay following a 72-h treatment.

b Mean ± SD from at least three independent experiments.

c Not determined.

d IC_50_(**MIX3**)/IC_50_(**3**)

e IC_50_(**MIX7**)/IC_50_(**7**)

As also shown in Table 2, the fold-changes between IC_50_(**MIX3**) and IC_50_(**3**), and between IC_50_(**MIX7**) and IC_50_(**7**), have been calculated. These results clearly indicate that the enhancement in potency is more pronounced for complex **7** compared with **MIX7** than for complex **3** compared with **MIX3**. To address the reviewer’s concern regarding statistical significance, we conducted a Student’s *t*-test analysis, with the corresponding *p*-values now provided in the footnotes to Table 2. The results demonstrate that the IC_50_ value of complex **3** differs significantly from that of **MIX3** across all tested cell lines, except A549. Likewise, the IC_50_ value of complex **7** is significantly different from that of **MIX7** in all cell lines. Furthermore, the calculated IC_50_ ratios, IC_50_(**MIX3**)/IC_50_(**3**) and IC_50_(**MIX7**)/IC_50_(**7**), ranged from 1.6 to 6.3 (see Table 2), thereby highlighting not only the statistical significance but also the biological relevance of the enhanced potency of complexes **3** and **7** compared with the corresponding mixtures.

Growth inhibition activity in cancer cell lines was compared with the activity to inhibit the proliferation of normal (noncancerous) cells, namely MRC-5, IMR-90, hTERT-HPNE, and MCF10A cells. The data is shown in Table 3. Selectivity indexes (SI) were calculated as (IC_50_ in noncancerous cells)/(average IC_50_ in cancer cells). The most favorable SIs were recorded for **7**, **6**, and **3**, ranging from 3.4 to 5.9. We further assessed the toxicity of several of the compounds in hepatocyte-like cells (HLC) generated from human pluripotent cells. The IC_50_ values in HLC were even higher than those in the other noncancerous cell lines, namely 9 ± 3 μM, 5 ± 1 μM, 8 ± 3 μM, and 4 ± 1 μM for **1**, **3**, **5**, and **7**, respectively.

**Table 3:** Cytotoxic/Antiproliferative Activity in Human Noncancerous Cell Lines and Chinese Hamster Cells (IC_50_ Values (μM))^a^ ^,^,^b^

	MRC-5	IMR-90	hTERT-HPNE	MCF10A	av cancer	av norm	SI^c^	CHOK1	MMC2	*F* ^d^
**1**	4.3 ± 0.3	7.6 ± 0.9	6.9 ± 0.8	6.4 ± 0.7	1.7	6.3	3.7	1.3 ± 0.5	1.4 ± 0.3	0.93
**2**	4.2 ± 0.9	7.4 ± 0.7	5.2 ± 0.6	5.1 ± 0.6	1.6	5.5	3.4	1.5 ± 0.6	0.77 ± 0.09	1.95
**3**	1.5 ± 0.6	2.3 ± 0.6	2.2 ± 0.4	2.6 ± 0.4	0.50	2.2	4.3	0.63 ± 0.05	0.23 ± 0.08	2.74(**)^e^
**4**	3.1 ± 0.9	2.9 ± 0.4	3.2 ± 0.6	2.4 ± 0.5	1.5	2.9	1.9	1.4 ± 0.4	1.3 ± 0.4	1.08
**5**	2.2 ± 0.5	3.7 ± 0.4	4.1 ± 0.2	3.4 ± 0.9	1.1	3.4	3.0	1.4 ± 0.6	1.3 ± 0.3	1.08
**6**	2.6 ± 0.9	4.1 ± 0.9	2.7 ± 0.5	2.6 ± 0.2	0.63	3.0	4.8	1.1 ± 0.2	0.29 ± 0.09	3.79(*)
**7**	1.2 ± 0.4	1.1 ± 0.3	2.0 ± 0.3	0.9 ± 0.2	0.22	1.3	5.9	0.46 ± 0.08	0.10 ± 0.03	4.60(**)
**8**	2.3 ± 0.6	5.0 ± 0.6	3.1 ± 0.6	2.9 ± 0.4	1.3	3.3	2.5	1.1 ± 0.2	0.9 ± 0.2	1.22
**cisPt**	10 ± 2	9.6 ± 0.8	12 ± 2	8.0 ± 0.9	8.8	9.9	1.1	24 ± 2	2.6 ± 0.6	9.23(***)

a Cell viability was determined with MTT assay following a 72 h treatment.

b MEAN ± SD from at least three independent experiments.

c Selectivity indexes defined as IC_50(normal cells)_/averageIC_50(cancer cells)_.

d Index *F* is defined as IC_50(CHOK1)_/IC_50(MMC2)_.

e Data was subjected to statistical analysis using Student’s *t*-test. The signs denote the significant difference between the two cell lines as follows: *p ≤ 0.05, **p ≤ 0.01, ***p ≤ 0.001.

In addressing the evaluation of the cytotoxic potential of the investigated compounds, it is interesting to note that the higher antiproliferative activity of the cisplatin-based complexes **3** and **7** compared to their oxaliplatin-based counterparts **4** and **8** could be expected because of their different reduction (Table 1) and subsequent activation of cisplatin-based complexes compared to oxaliplatin analogues. However, *in vivo*, the situation could be completely different. This observed trend aligns with existing clinical findings, namely that cisplatin-based regimens generally demonstrate equal or superior efficacy compared to oxaliplatin-based treatments.^30,31^ Specifically, in most NSCLC settings, cisplatin (or other classic cisplatin-based doublets) remains the standard of care, largely due to producing comparable or slightly better response and survival outcomes than oxaliplatin combinations in similar clinical contexts.^32^

The cytotoxicity assay was also employed to determine whether DNA damage might be involved in the mechanism of action of the tested compounds. The Chinese hamster ovary MMC2 cell line harbors the ERCC3/XPB mutation, which renders it deficient in nucleotide excision repair (NER), whereas the parent line CHOK1 is proficient in this repair process. Thus, the MMC2 line is often more sensitive to agents causing DNA damage, especially NER-reparable DNA damage, than CHOK1. As shown in Table 3, cisplatin, an established DNA-damaging agent, is approximately 9 times more potent in MMC2 than in CHOK1. Among the investigated compounds, **3**, **6**, and **7** exhibit significant differences in cytotoxicity between the two cell lines. Interestingly, the ceritinib-containing agents appear to be more DNA-damaging than the crizotinib-derived complexes. Although the factors *F* do not reach the value for cisplatin, it might be deduced that DNA damage might also be included in the mechanism of action of these compounds. To support this finding, we detected the amount of platinum bound to DNA in samples exposed to cisplatin, **3**, **7**, **MIX3**, and **MIX7**. The results shown in Figure S36 indicate that the DNA is platinated to a similar extent in all the samples, with values of 16.5, 19.9, 14.6, 24.3, and 20.0 pg Pt/μg DNA, respectively. Moreover, we detected γH2AX foci in NCI-H2228 cells treated with **3**, **7**, and cisplatin (Figure S37). Phosphorylation of H2AX is a commonly used marker of DNA damage.^33^ These results indicate that DNA represents, at least to some extent, a target of the action of **3** and **7**, presumably through the action of cisplatin released intracellularly from the Pt­(IV) complexes.

Crizotinib and ceritinib are used to treat advanced NSCLC with alterations in ALK (anaplastic lymphoma kinase). Further experiments were therefore conducted on lung cancer cell lines, human lung carcinoma (A549) and human nonsmall cell lung carcinoma with ALK mutation (NCI-H2228). Complexes **3** and **7** were chosen for the advanced studies.

### Cytotoxicity in 3D Spheroids

The cytotoxic activity of the novel group of compounds was also evaluated in the 3D cellular spheroid model, which better mimics the situation in solid tumors than 2D arrangements. In the 3D structure, the gradients of gases, nutrients, and therapeutics play a role in the final cytotoxicity of a compound. We investigated the activity of the most effective cisplatin-derived complexes in each group, **3** and **7**, the ligands **1** and **5**, as well as the mixtures **MIX3** and **MIX7**. In the 3D spheroids, **3** and **7** were the most active agents in both cell lines, with IC_50_s ranging from 0.11 to 0.98 μM (Table 4).

**Table 4:** Activity of the Compounds in 3D Spheroids^a^ of A549 and NCI-H2228 Cells Expressed as IC_50_ Values (μM)^b^

	A549	NCI-H2228
**1**	3 ± 1	1.5 ± 0.3
**3**	0.98 ± 0.05	0.41 ± 0.07
**MIX3**	2.6 ± 0.4	0.9 ± 0.1
**5**	2.3 ± 0.8	0.75 ± 0.09
**7**	0.43 ± 0.07	0.11 ± 0.03
**MIX7**	1.8 ± 0.2	0.71 ± 0.06
**cisPt**	19 ± 3	2.1 ± 0.9

a The cell viability was assessed with CellTiter-Glo 3D Cell Viability Assay.

b The spheroids were treated for 96 h.

Whereas IC_50_ values of **1** and **5** in the 3D model were higher than those in the 2D model, IC_50_ values of **3** and **7**, especially in NCI-H2228 spheroids, were comparable to those of the 2D model. The reason might be that the Pt­(IV) complexes **3** and **7** penetrate the NCI-H2228-derived spheroids more easily than the other compounds. It is noteworthy that **3** and **7** were significantly more potent than cisplatin or the mixture of their components, and more potent than crizotinib and ceritinib, respectively, attesting to the advantages of multiaction prodrugs.

### Cellular Accumulation

Since cellular accumulation is an early and essential event in the process of cell growth inhibition, we evaluated the cellular accumulation of the platinum-containing compounds. NCI-H2228 and A549 cells were exposed to 1 μM complexes for 4 h, and the Pt associated with the cells was determined using inductively coupled plasma mass spectrometry (ICP-MS). As shown in Figure 4, the compounds with the phenylbutyrate ligand (**3** and **7**) accumulated more readily in both A549 and NCI-H2228 cells than the monosubstituted complexes (**2** and **6**). It has been previously reported that the phenylbutyrate ligand increases the lipophilicity of Pt­(IV) agents, thereby facilitating cellular accumulation.^27^ Complexes **4** and **8**, derived from oxaliplatin, entered the cells less efficiently than the cisplatin-derived monosubstituted **2** and **6**, despite having the phenylbutyrate moiety in the axial position. All the compounds were taken up by A549 cells slightly better than by NCI-H2228 cells. This is not in line with the IC_50_ values determined in the previous experiment, where these complexes show slightly better activity in NCI-H2228 cells than in A549 cells. The amounts of ceritinib derivatives associated with the cells were roughly 2-fold higher than those of crizotinib derivatives in both cell lines. Of all the Pt compounds investigated in this work, **7** entered the cells most readily, followed by **6** and **3**.

**Figure 4:**
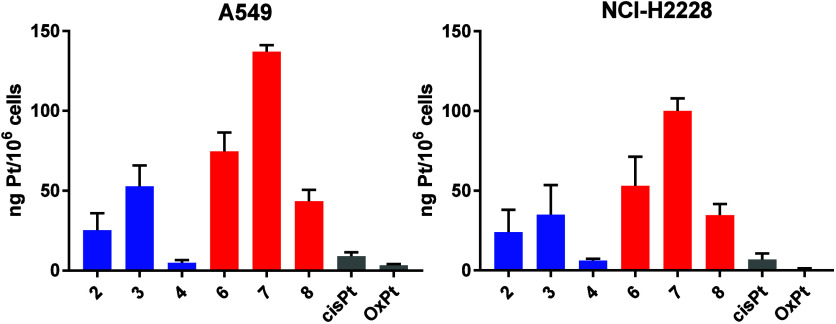
Cellular accumulation of the complexes. A549 and NCI-H2228 cells were exposed to a 1 μM concentration of the Pt-containing complexes for 4 h. The platinum amount in cell lysates was determined using ICP-MS. The data show the mean ± SD from two experiments.

When discussing the results describing the cellular accumulation of the investigated compounds, it is essential to mention the following. In the experiments described in this study, cellular accumulation was measured, which depends on influx, efflux, and the rate of biotransformation of the complexes in cells (in this case, particularly the rates of reduction and subsequent reaction in the platinum­(II) state). In this context, it is unsurprising that the cisplatin-derived complexes accumulated more than those derived from oxaliplatin since they are expected to undergo reduction and biotransformation at greater rates.^34,35^

Additionally, in the present study, the intracellular reduction of Pt­(IV) prodrugs produces either cisplatin or oxaliplatin. Many previous studies have demonstrated notable differences between these two agents in terms of their mechanisms of action, including differences in DNA binding kinetics, the size and structure of DNA adducts, interference with RNA and DNA polymerases due to steric effects, and recognition by DNA-damage response proteins. Additionally, oxaliplatin has been found to work through other mechanisms not shared by cisplatin.^36^ Therefore, it is not reasonable to expect a direct link between cellular uptake and cytotoxic activity when comparing compounds based on oxaliplatin and cisplatin.

### Effect on Cell Invasivity

Since crizotinib and ceritinib have been reported to possess anti-invasive properties,^37,38^ we investigated the potential of **3** and **7** (and for comparison **1**, **5**, **MIX3**, **MIX7**, and cisplatin) to affect cell migration/invasion. First, we employed the scratch test. The ability to slow the cell closing the open area was observable for all tested compounds to a similar extent (Figure S38). Whereas the open area was almost completely closed after 48 h in nontreated cells, it remained almost fully clear of cells in the treated samples. The evaluation of the results is shown in Figure 5A,B. Complex **3** and especially **7** displayed antimigratory potential comparable to that of crizotinib and ceritinib, although at lower concentrations (1.7-fold and 3.9-fold, respectively). Cisplatin alone reduced the area filling only partially.

**Figure 5:**
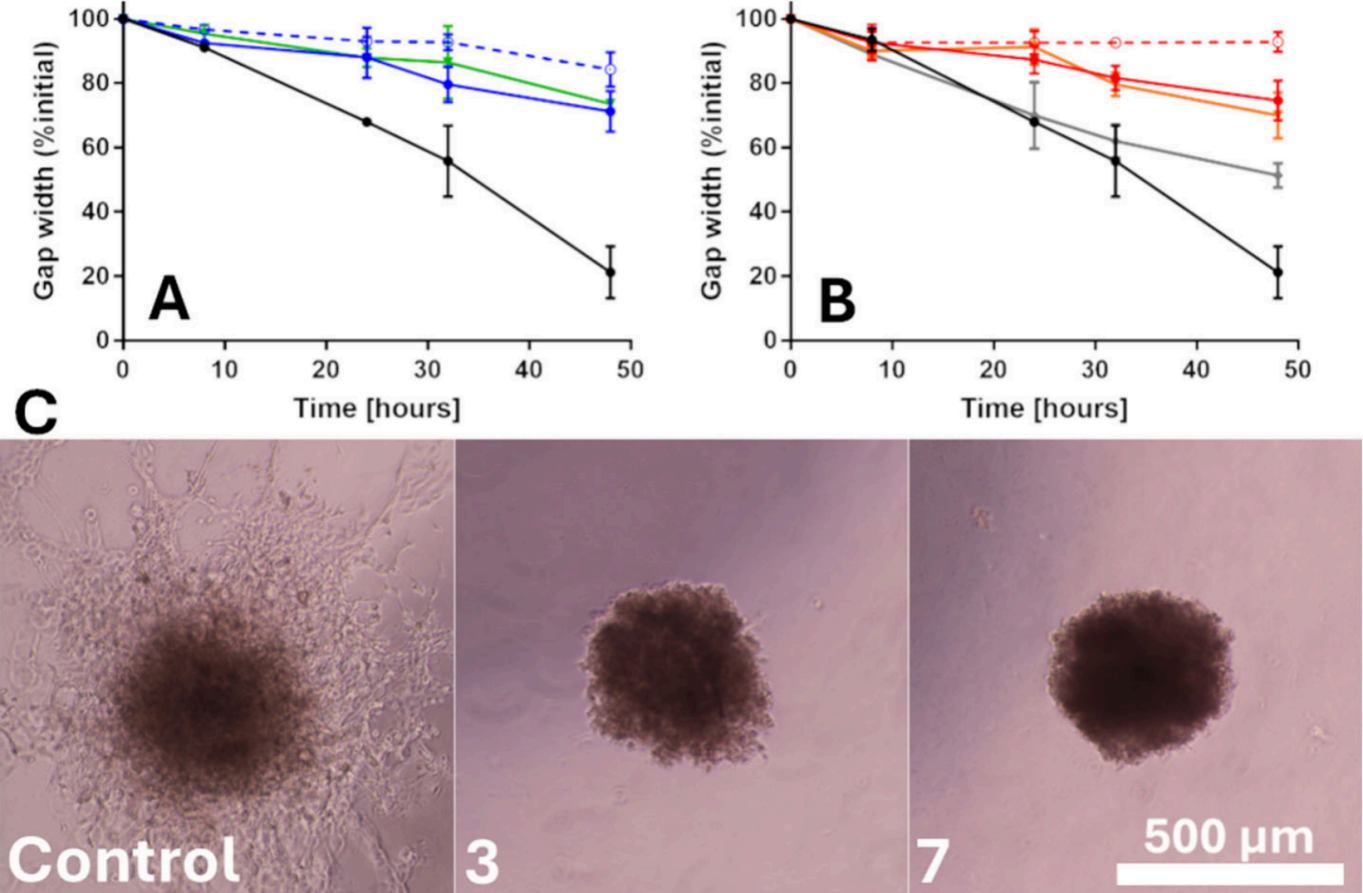
Antimigratory/anti-invasive effect. (A) and (B) Scratch test. (A) A549 cells were treated with **1**, blue-dashed; **3**, blue; **MIX3**, green. (B) A549 cells were treated with **5**, red-dashed; **7**, red, **MIX7**, orange; and cisPt, gray. The black line corresponds to nontreated cells. The results show the average evaluation from three wells. (C) Spheroid outgrowing into the Matrigel. Representative images of NCI-H2228 cells, nontreated or treated with **3** and **7** at concentrations corresponding to IC_50_ values.

Another experiment used to demonstrate the anti-invasive properties of the compounds involved monitoring the outgrowth of A549 and NCI-H2228 spheroids into the surrounding matrix. The spheroids were embedded in Matrigel, treated with the tested compounds, and monitored for 72 h (Figure 5C and Figure S39). The images of the spheroids indicate that the treatment with the investigated compounds almost completely inhibited the spheroid invasion into the Matrigel. Again, the effects of **3** and **7** were comparable to those of **1** and **5**, respectively, despite lower doses being used.

Both experiments demonstrate that Pt­(IV) complexes **3** and **7** inhibit the migratory and invasive abilities of A549 and NCI-H2228 cancer cells.

### Cell Cycle Modulation

The alterations in cell cycle profiles caused by the tested agents in NCI-H2228 cells may offer insight into their anticancer mechanism. To examine these changes, we utilized flow cytometry to analyze the cells treated with the representative agents (Figure 6 and Figure S40).

**Figure 6:**
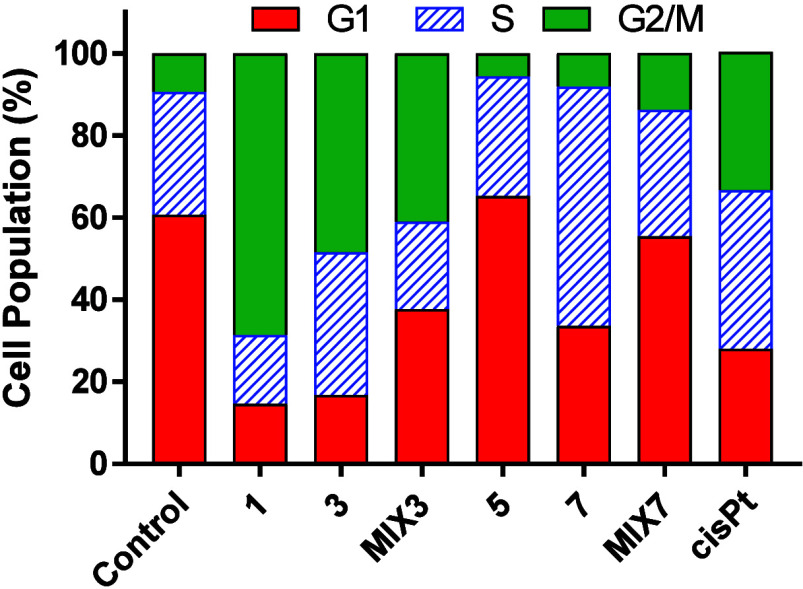
Cell cycle distribution. NCI-H2228 cells were treated with the tested compounds at concentrations corresponding to 2 × IC_50_ for 48 h. The cell distribution into individual phases of the cell cycle was performed using flow cytometry based on propidium iodide staining. The experiment was performed twice. Error bars are omitted for clarity.

As shown in Figure 6, there is a prominent difference between the profiles of cells exposed to **3** and **7**. Whereas **3** induced arrest in the G2/M phase, the majority of the population in cells treated with **7** was in the S phase. It appears that the G2/M arrest is a common phenomenon in the crizotinib-containing group of NCI-H2228 cells under the conditions used. Published papers report both G0/G1 and G2/M arrests in varying percentages depending on cell type, time of treatment, and concentration of crizotinib used.^39,40^

The population distribution of cells exposed to **7** differs from samples of cells exposed to **5** and **MIX7**. Ceritinib is reported to halt cell division and prevent the progression of cancer cells through the G1 phase.^41^ A similar profile characterizes the populations in **MIX7**-treated cells. The distinct cell cycle profile of **7**-treated NCI-H2228 cells might result from the combined mechanisms of action of the constituents of **7** (ceritinib, cisplatin, and phenylbutyrate).

The different cell cycle profiles of **3** and **7** might suggest that the mechanism of action (MOA) of these complexes might be different, and while the MOA of **3** seems to be similar to that of **1**, the MOA of **7** might differ from that of ceritinib alone, as well as from that of the mixture of its constituents.

### Detection of Cell Death Mode

Flow cytometric analysis was used to assess the mode of cell death induced in NCI-H2228 cells by the tested complexes. Annexin V staining was employed to recognize the externalization of phosphatidyl serine, a feature considered an apoptotic marker.^42^ Propidium iodide (PI) was used to stain cells with compromised cell membranes (a sign of necrosis). Staurosporine was included in the experiment as a positive control for apoptosis. The results are shown in Figure S41. The cells exposed to **3** and **7** exhibit an apoptotic phenotype after 48 h of treatment. Apoptosis appears to be the leading mode of cell death in the entire group of tested compounds, with a varying distribution of cell populations between early and late apoptosis.

### ALK Kinase Inhibition

To assess whether crizotinib or ceritinib exercises their biological activity upon their intracellular release, we performed Western blot detection of the ALK phosphorylation state in lysates of NCI-H2228 cells exposed to the tested compounds. In Figure 7, representative Western blot images show that **3** and **7**, as well as the ligands alone (**1** and **5**), and **MIX3** and **MIX7** inhibit the phosphorylation of ALK, as well as another receptor tyrosine kinase implicated in the progression of various cancer types (MET). Whereas the signals of pALK and pMET are decreased in the presence of the tested compounds, the overall amount of ALK and MET is not markedly changed. The results showing decreased phosphorylation of ALK and MET caused by **3** and **7** support our findings that the Pt­(IV) agents are reduced within the cells while simultaneously releasing bioactive ligands such as crizotinib or ceritinib. Cisplatin had no visible effect on the phosphorylation state of ALK and MET under the conditions employed.

**Figure 7:**
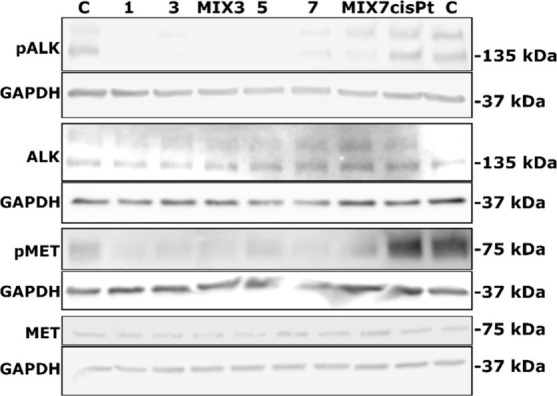
Western blot analysis of the phosphorylation state of ALK and MET. NCI-H2228 cells were treated with the indicated compounds at concentrations corresponding to their IC_50_ values for 24 h. Raw images are provided in Figure S42.

### Immunogenic Cell Death

Since crizotinib and ceritinib are reported to induce ICD,^43,44^ the primary objective of this work was to assess the potency of the new Pt­(IV) complexes to induce ICD in cancer cells. To achieve this goal, we used the highly immunogenic murine colorectal cell line CT26 alongside the lung NCI-H2228 cells (Figure 8).

**Figure 8:**
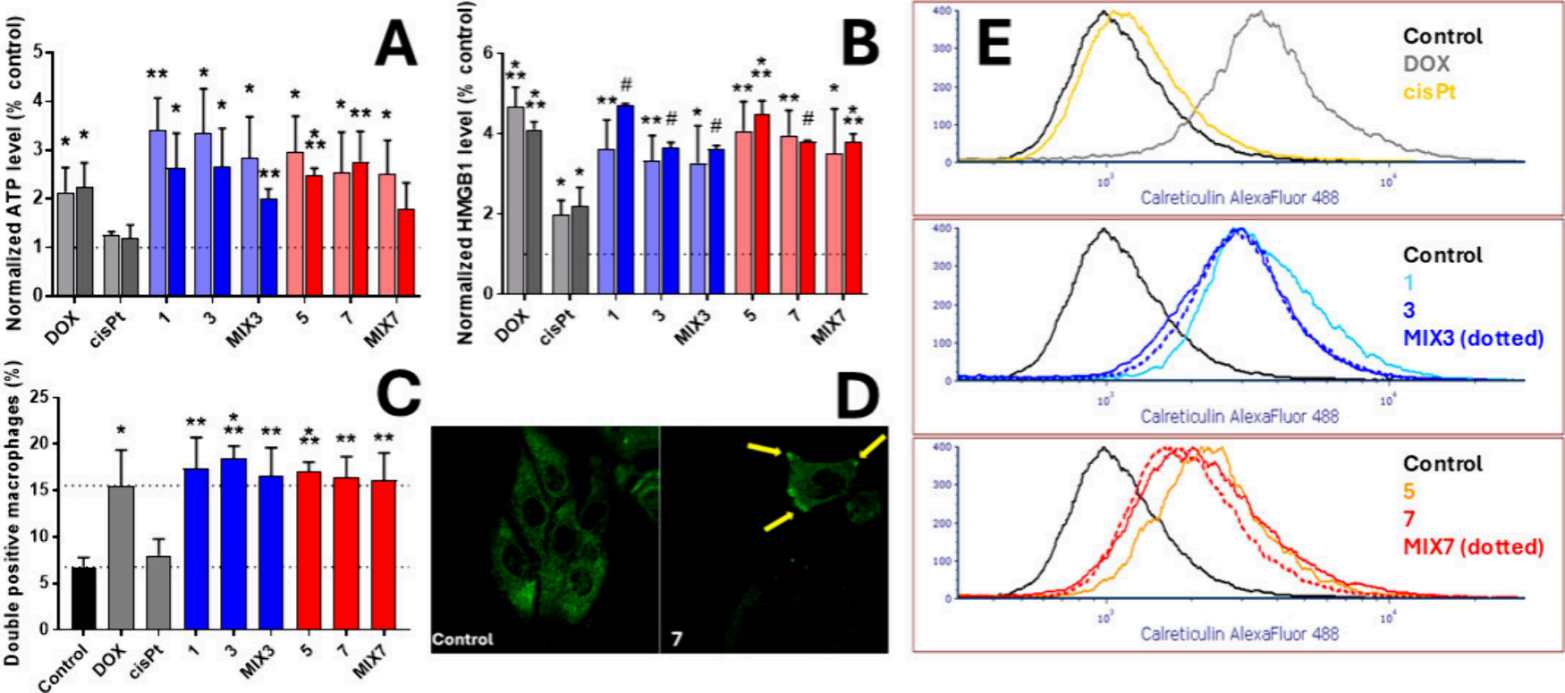
Immunogenic cell death. (A) Normalized values of extracellular ATP levels in NCI-H2228 (light colors) and CT26 (dark colors) cells, either nontreated or treated with the indicated compounds. The value of ATP determined for the nontreated control was set as 1. (B) Normalized values of externalized HMGB1 following the treatment of NCI-H2228 (light colors) and CT26 (dark colors) with the indicated compounds for 24 h. The value of HMGB1 determined for the nontreated control was set as 1. (C) Phagocytosis: CT26 cells were treated with the tested compounds and cocultivated with J774.A1 macrophages. The graph shows percentages of double-stained populations. Data in (A–C) were subjected to statistical analysis using Student’s *t*-test. The signs denote the significant difference from the untreated control as follows: *p ≤ 0.05, **p ≤ 0.01, ***p ≤ 0.001, and #*p* ≤ 0.0001. (D) Externalization of calreticulin, representative images. NCI-H2228 cells were either untreated or treated with **7**, fixed, and stained with an anticalreticulin antibody conjugated to AlexaFluor 488, and then recorded using a confocal microscope. Regions with a strong calreticulin signal are visible in the cell treated with **7** (yellow arrows). (E) Externalization of calreticulin. CT26 cells were nontreated or treated with indicated compounds at concentrations corresponding to IC_50_ values for 16 h, stained with anticalreticulin antibody, and analyzed with flow cytometry.

### Externalization of Calreticulin

During ICD, dying cells expose or release various hallmarks collectively known as damage-associated molecular patterns (DAMPs). Among the early DAMPs involved in ICD is calreticulin, a 46 kDa Ca^2+^ binding protein. Calreticulin is localized in the endoplasmic reticulum under physiological conditions.^45^ Upon effective stimulation of endoplasmic reticulum stress or induction of ICD, however, calreticulin undergoes translocation to the peripheral regions, specifically to the cytoplasmic membrane. This feature is considered an early “eat-me” signal for immune effector cells. The presentation of calreticulin on the cell surface attracts cytotoxic T-lymphocytes to the dying cells.^46^ The exposure of calreticulin powerfully triggers the cancer vaccination effect - an essential and transformative therapeutic advantage of ICD-inducing treatments.^47^

CT26 and NCI-H2228 cells were treated with the selected compounds for 16 h at concentrations corresponding to their respective IC_50_ values (IC_50_ values determined using the MTT assay for the CT26 cell line are shown in Table S1). The cells were stained with anticalreticulin-Alexa 488 antibody and analyzed with flow cytometry. Cells with compromised cell membranes (PI-positive) were excluded from the analysis. The results are shown in Figure 8E. The externalization of calreticulin was apparent in cells treated with **3** and **7**. The effect of **3** mimicked the effect of **1** and **MIX3**, and at the same time, the effect of **7** was similar to the impact of **5** and **MIX7**. The crizotinib-containing agents stimulated calreticulin externalization to a higher extent (comparable to **DOX**) than the ceritinib-containing compounds. The fluorescence intensity related to the externalized calreticulin was similar in both groups – the crizotinib group (**1**, **3**, and **MIX3**) and the ceritinib group (**5**, **7**, and **MIX7**), despite using different compound concentrations based on the IC_50_ values. It appears that equitoxic doses within each group stimulate a similar extent of calreticulin exposure. The induction of calreticulin externalization was weakest in cells exposed to cisplatin. The results show that all the tested compounds (except cisPt) may be effective inducers of ICD.

To visualize calreticulin exposure on the cell surface, we treated NCI-H2228 cells with compounds at equitoxic concentrations (IC_50_) for 16 h, fixed the cells, and stained them with an anticalreticulin antibody and a secondary antibody conjugated to AlexaFluor 488. The images are shown in Figure 8D and Figure S43. Spots with a strong calreticulin signal in the cell membrane region are visible in the treated cells.

### Release of ATP

Another hallmark considered as DAMP is the release of ATP. ATP externalization activates dendritic cell precursors and specific receptors (P2X7) on dendritic cells.^48^ The activation cascade leads to the secretion of interleukins and the release of specific cytokines, which consequently stimulate the immune response.^49^

ATP release was analyzed using the CLSII bioluminescence assay kit (Roche) after treatment of CT26 or NCI-H2228 cells with the tested compounds at concentrations corresponding to their respective IC_50_ values (Figure 8A). The amount of ATP released to the medium by cells dosed with **3** was 3.3-fold higher than that of nontreated cells and comparable to the values determined in cells exposed to **1**, despite the lower concentration of **3** used. The effect caused by **7** is 2.7 times that of the nontreated control and similar to the effect of **5**, even at a lower concentration of **7**. Cisplatin did not induce a significant release of ATP. Pt­(IV) complexes derived from cisplatin and containing crizotinib or ceritinib stimulate ATP externalization in CT26 and NCI-H2228 cells.

### Detection of Extracellular HMGB1

HMGB1 is a nonhistone chromatin-binding protein typically located in the cell nucleus. Under certain conditions, such as senescence, the protein is released from the nucleus of the cell and into the extracellular environment. HMGB1 released from the cells can activate dendritic cells through binding to TLR4 receptors, and thus, dendritic cells facilitate antigen presentation to the T cells.^50^ The release of HMGB1 belongs to the DAMP family.

CT26 and NCI-H2228 cells were treated with the investigated compounds at concentrations corresponding to their respective IC_50_ values for 24 h. The amount of HMGB1 in the medium was determined using an enzyme-linked immunosorbent assay (ELISA). The results shown in Figure 8B indicate that **3** and **7**, along with the other compounds, stimulated HMGB1 externalization to varying extents, ranging from 3.3-fold to 4.7-fold the value of the nontreated control. The lowest increase was noted for cisplatin-treated cells (2-fold), while the highest increase was observed in cells exposed to doxorubicin and **1**. All the crizotinib and ceritinib-containing compounds were able to induce HMGB1 release in CT26 and NCI-H2228 cells, although **3** and **7** were able to produce the effect at lower concentrations.

In sum, the three DAMPs analyses indicate that the crizotinib and ceritinib containing Pt­(IV) complexes induce calreticulin cell surface exposure and ATP and HMGB1 release in NCI-H2228 and CT26 cells to the extent similar to that of crizotinib and ceritinib alone or in the mixture with cisplatin and phenylbutyrate although at equitoxic concentrations, that means at concentrations 2-fold (**3**) or 4.5–7.1-fold (**7**) lower.

### Detection of Phagocytosis

Phagocytosis is a crucial immune-mediated mechanism that directly targets and eliminates cancer cells undergoing ICD and is primarily carried out by macrophages, neutrophils, natural killers, and certain specialized lymphocyte populations.^51^ Given that CT26 tumor cells exposed to the investigated compounds exhibited key characteristics of ICD, we explored their susceptibility to phagocytosis by macrophages through an *in vitro* assay.

CT26 cells were exposed to the investigated compounds at concentrations corresponding to IC_50_ values for 24 h. J774.A1 macrophages and the CT26 cells were then stained with the CellTracker green and red fluorophores, respectively, and cocultivated for 4 h at the J774.A1: CT26 ratio of 1:2. Subsequent flow cytometry analysis identified the double-stained population as phagocytotic cells (Figure 8C, Figure S44). The double-stained populations in samples with CT26 cells treated with **3** and **7** were significantly increased compared to nontreated control cells or cells treated with cisplatin. This was similar to the double-stained population in the sample with doxorubicin-treated CT26 cells, as well as the cells exposed to **1** and **5**, although at lower concentrations. The treatment of CT26 cells with crizotinib and ceritinib containing Pt­(IV) compounds stimulated phagocytosis of the cells with J774.A1 macrophages in the *in vitro* experiment.

## Conclusions

The newly synthesized and characterized Pt­(IV) complexes containing either crizotinib or ceritinib are effective in inhibiting the growth of cancer cells. The derivatives of cisplatin functionalized with phenylbutyrate and either crizotinib or ceritinib are the most efficient complexes in each group. Moreover, these compounds (**3** and **7**), along with monofunctionalized **6**, showed promising selectivity indices toward cancer over noncancerous cells. In addition to their effectiveness against cancer cells in a 2D arrangement, the agents also inhibited the growth and viability of A549 and NCI-H2228 cells in the spheroid model. Of the platinum-containing compounds, **7**, **6**, and **3** were the agents most easily accumulated in A549 and NCI-H2228 cells. The scratch test demonstrated that **3** and **7** inhibited A549 cell migration to a similar extent as **1** and **5**, albeit at lower concentrations. The outgrowth of A549 and NCI-H2228 spheroids into the Matrigel was also reduced in the presence of **3** and **7**. Whereas crizotinib-containing compounds arrested the cell cycle in G2/M phase, only a minor population of cells exposed to ceritinib-containing agents belonged to G2/M. A prominent population of cells exposed to **7** was arrested in S phase. The cytotoxic activity in NER proficient and deficient cells also indicated that the MOA of ceritinib-containing complexes **6** and **7** and crizotinib-containing complex **3** might involve DNA damage. This finding was supported by the detected DNA platination and phosphorylation of H2AX in NCI-H2228 cells dosed with **3** and **7**. Pt­(IV) complexes derived from cisplatin and containing crizotinib and ceritinib induced apoptosis in NCI-H2228 cells. Moreover, the Western blot analysis of NCI-H2228 cells exposed to some of the compounds showed that crizotinib and ceritinib are intracellularly released from **3** and **7** in their active form, able to act as tyrosine kinase inhibitors.

Furthermore, it has been shown that CT26 and NCI-H2228 cells exposed to **3** and **7** triggered responses collectively known as DAMPs (damage-associated molecular patterns), including the exposure of calreticulin to the cell surface and the externalization of ATP and HMGB1. These factors are prerequisites for immunogenic cell death, and we also demonstrated that phagocytosis by J774.A1 macrophages was induced when CT26 cells were treated with the investigated compounds. At the same time, **3** and **7** were as effective as **1** and **5** (and **MIX3** and **MIX7**), although used at lower concentrations.

In conclusion, the new Pt­(IV) complexes derived from cisplatin or oxaliplatin, containing either crizotinib or ceritinib, are potent anticancer agents. The best performers of each group, **3** and **7**, were shown to combine cytotoxicity with immune and anti-invasive activation in cancer cell models.

## Experimental Section

### Materials and Methods

All the chemicals and solvents were purchased from multiple commercial sources and used as received unless otherwise specified. Crizotinib (CAS no. 877399-52-5) and ceritinib (CAS no. 1032900-25-6) were purchased from 1PlusChem. The progress of the reactions was monitored using an analytical HPLC system (Thermo Scientific UltiMate 3000) with a reverse-phase C18 column (Phenomenex Kinetex, 100 mm length, 4.60 mm internal diameter, 2.6 μm Particle size, 100 Å pore size). The purity and retention time (RT) of the synthesized compound reported here were measured using the same analytical HPLC system, with a 0.1% trifluoroacetic acid (TFA) in water and acetonitrile gradient at a flow rate of 1 mL min^–1^. Reaction mixtures were purified on a preparative HPLC system (Thermo Scientific UltimaMate 3000 station) equipped with a reverse-phase C18 column (Phenomenex Luna, 250 mm × 21.2 mm, 10 μm, 100 Å), using a similar type of mobile phase at a flow rate of 15 mL min^–1^. UV detection was set at 220 nm. The fractions were combined and lyophilized to get the pure compounds. The newly synthesized Pt­(IV) complexes were characterized by ^1^H NMR, ^195^Pt NMR, ESI-MS, HPLC, and elemental analysis. All the complexes (**2**–**4** and **6**–**8**) have high purity (>95%) as determined by HPLC measurements and elemental analysis. All NMR data were collected on a Bruker AVANCE IIITM HD 500 MHz spectrometer. The data were processed using either MestReNova or Bruker TopSpin 3.6.0 software. ^1^H chemical shifts were referenced with the individual solvent residual peaks of the respective NMR solvents used. ^195^Pt NMR chemical shifts were reported with respect to the chemical shift of standard K_2_PtCl_4_ in water at −1624 ppm. Electrospray ionization mass spectra (ESI-MS) were done using a Thermo Scientific triple quadrature mass spectrometer (Quantum Access) by + ve mode electrospray ionization. Elemental analyses reported were performed using a Thermo Scientific FLASH 2000 element analyzer.

### Synthesis

#### Synthesis of *ctc*-[Pt­(NH_3_)_2_(crizotinib)­(OH)­Cl_2_] (**2**)

Oxoplatin (60 mg, 0.18 mmol) was stirred overnight with 49 mg of *N,N′*-disuccinimidyl carbonate (DSC) (0.19 mmol, 1.05 equiv) in 7 mL of DMSO at room temperature. Upon completion of the reaction, as indicated by ^195^Pt NMR, the reaction mixture was filtered, and the mother liquor was treated with large amounts of diethyl ether, affording a two-phase system. The ether phase was removed after the sample was centrifuged. The procedure was repeated several times until a sticky yellow solid was obtained. It was then suspended in a minimum amount of acetonitrile and then precipitated in diethyl ether. The precipitate was collected by centrifugation and was used for the next step without further purification. Yield: 58 mg (65.8%). To the solution of crizotinib (46 mg, 0.102 mmol) in DMF (5 mL) activated *ctc*-[Pt­(NH_3_)_2_(MSC)­(OH)­Cl_2_] (58 mg, 0.12 mmol) was added and stirred at 45 °C for 2 h followed by incubation at room temperature for 2 h. The formation of a new peak at RT = 4.16 min was observed by analytical HPLC. The reaction mixture was concentrated by evaporation and then diluted with acetonitrile before being injected into a preparative HPLC system. The product was isolated by injecting this solution into a preparative HPLC system using a gradient of 0.1% TFA in water and acetonitrile. The product isolated was concentrated and lyophilized. Yield: 56 mg (51%). RP-HPLC (analytical): RT= 4.16 min. ^1^H NMR (500 MHz, DMSO, 298 K) δ­(ppm): 8.06 (s, 1H), 7.74 (d, *J* = 1.6 Hz, 2H), 7.67–7.58 (m, 2H), 7.49 (t, *J* = 8.7 Hz, 1H), 7.12 (d, *J* = 1.3 Hz, 1H), 6.53–5.99 (m, 7H), 4.34 – 4.27 (m, 1H), 4.14 (d, *J* = 8.9 Hz, 2H), 2.86 (dd, *J* = 21.7, 9.9 Hz, 2H), 1.96 (d, *J* = 9.9 Hz, 2H), 1.86 (d, *J* = 6.6 Hz, 3H), 1.76 (d, *J* = 8.3 Hz, 2H). ^195^Pt NMR (107.5 MHz, DMSO, 298 K) δ (ppm): 1092. ESI-MS (+ve mode): *m*/*z* calculated for [C_22_H_29_Cl_4_FN_7_O_4_Pt^+^] 811.06 found 810.02. Elemental analysis calculated for [C_22_H_28_Cl_4_FN_7_O_4_Pt·2CF_3_COOH·3H_2_O] C, 28.58; H, 3.32; N, 8.97; Observed: C, 28.49; H, 3.01; N, 8.81.

#### Synthesis of *ctc*-[Pt­(NH_3_)_2_(crizotinib)­(PhB)­Cl_2_] (**3**)

*ctc*-[Pt­(NH_3_)_2_(PhB)­(OH)­(Cl)_2_] (72 mg, 0.15 mmol) was dissolved in DMF, and 1.2 equiv of DSC (46.4 mg, 0.18 mmol) was added, which was stirred at room temperature for 45 min. The DMF was evaporated under reduced pressure, and the sticky residue was resuspended in acetonitrile and precipitated in diethyl ether. The precipitate was collected by centrifugation and washed twice with diethyl ether and used for the next step without further purification. Yield: 73 mg (78%). To the solution of crizotinib (50.2 mg, 0.11 mmol) in DMF (2 mL) activated ctc-[Pt­(NH_3_)_2_(MSC)­(PhB)­Cl_2_] (68 mg, 0.109 mmol) was added in 2 mL DMF and stirred at room temperature. The progress of the reaction was monitored by HPLC. The new peak appeared at RT = 5.1 min (analytical HPLC). After 3h the reaction mixture was concentrated and diluted with acetonitrile. The final product was isolated by injecting the solution into a preparative HPLC system using a gradient of 0.1% TFA in water and acetonitrile. The product was concentrated and lyophilized. Yield: 71.9 mg (55%). RP-HPLC (analytical): RT= 5.09 min. ^1^H NMR (500 MHz, DMSO, 298 K) δ (ppm): 8.06 (s, 1H), 7.74 (d, *J* = 1.4 Hz, 2H), 7.66–7.58 (m, 2H), 7.48 (t, *J* = 8.7 Hz, 1H), 7.30–7.14 (m, 5H), 7.12 (d, *J* = 1.0 Hz, 1H), 6.63 (s, 6H), 6.26 (d, *J* = 6.7 Hz, 1H), 4.31 (td, *J* = 11.3, 5.6 Hz, 1H), 4.14 (s, 2H), 2.87 (d, *J* = 16.7 Hz, 2H), 2.59 (t, *J* = 7.6 Hz, 2H), 2.23 (t, *J* = 7.4 Hz, 2H), 1.96 (d, *J* = 9.9 Hz, 2H), 1.88–1.69 (m, 7H). ^195^Pt NMR: 107.5 MHz, DMSO, 298 K: δ (ppm) 1230. ESI-MS (+ve mode): *m*/*z* calculated for [C_32_H_39_Cl_4_FN_7_O_5_Pt^+^] 957.13 found 957.1. Elemental analysis calculated for [C_32_H_38_Cl_4_FN_7_O_5_Pt·1.5CF_3_COOH·1.5H_2_O] C, 36.41; H, 3.71; N, 8.49; Observed: C, 36.45; H, 3.74; N, 8.39.

#### Synthesis of *ctc*-[Pt­(DACH)­(crizotinib)­(PhB)­(Ox)] (**4**)

*ctc*-[Pt­(DACH)­(crizotinib)­(PhB)­(Ox)] was synthesized using the same procedure as explained above (for complex **3**) using *ctc*-[Pt­(DACH)_2_(PhB)­(OH)­(Cl)_2_] as precursor. Yield: 56%. RP-HPLC (analytical): RT= 5.08 min. ^1^H NMR (500 MHz, DMSO) δ (ppm): 9.26 (d, *J* = 121.8 Hz, 1H), 8.60 (s, 1H), 8.32 (d, *J* = 65.0 Hz, 2H), 8.06 (s, 1H), 7.74 (d, *J* = 1.6 Hz, 2H), 7.67–7.59 (m, 2H), 7.49 (t, *J* = 8.7 Hz, 1H), 7.26 (dd, *J* = 10.3, 4.6 Hz, 2H), 7.20–7.11 (m, 4H), 6.27 (q, *J* = 6.6 Hz, 1H), 4.39–4.30 (m, 1H), 4.03 (s, 2H), 2.90 (dd, *J* = 13.0, 10.3 Hz, 2H), 2.55 (d, *J* = 7.4 Hz, 3H), 2.33–2.25 (m, 2H), 2.21–2.10 (m, 2H), 1.96 (d, *J* = 9.9 Hz, 2H), 1.86 (d, *J* = 6.6 Hz, 3H), 1.79–1.61 (m, 4H), 1.56–1.35 (m, 4H), 1.16 (d, *J* = 7.6 Hz, 2H). ^195^Pt NMR: 107.5 MHz, DMSO, 298 K: δ (ppm) 1596. ESI-MS (+ve mode): *m*/*z* calculated for [C_40_H_47_Cl_2_FN_7_O_9_Pt^+^] 1053.24 found 1053.76. Elemental analysis calculated for [C_40_H_46_Cl_2_FN_7_O_9_Pt·1.5CF_3_COOH·3H_2_O] C, 40.38; H, 4.22; N, 7.67; Observed: C, 40.23; H, 3.96; N, 7.77.

#### Synthetic Procedure of the Pt­(IV)–Ceritinib Complexes (**6**-**8**)

Pt­(IV) complexes **6**–**8** were synthesized using the same procedure as explained above for the complexes **2**–**4**, respectively, using ceritinib (**5**) as the ligand instead of crizotinib.

#### *ctc*-[Pt­(NH_3_)_2_(ceritinib)­(OH)­Cl_2_] (**6**)

Yield: 40%. RP-HPLC (analytical): RT= 5.11 min. ^1^H NMR (500 MHz, DMSO) δ (ppm): 9.52 (s, 1H), 8.43 (d, *J* = 8.2 Hz, 1H), 8.26 (s, 1H), 8.17 (s, 1H), 7.84 (dd, *J* = 8.0, 1.3 Hz, 1H), 7.63 (t, *J* = 7.8 Hz, 1H), 7.48 (s, 1H), 7.37 (t, *J* = 7.6 Hz, 1H), 6.79 (s, 1H), 6.42 (s, 6H), 4.54 (dt, *J* = 12.2, 6.1 Hz, 1H), 4.20 (s, 2H), 3.48–3.41 (m, 1H), 2.86–2.71 (m, 3H), 2.13 (s, 3H), 1.66–1.44 (m, 4H), 1.21 (d, *J* = 6.0 Hz, 6H), 1.15 (d, *J* = 6.8 Hz, 6H). ^195^Pt NMR: 107.5 MHz, DMSO, 298 K: δ (ppm) 1110. ESI-MS (+ve mode): *m*/*z* calculated for [C_29_H_43_Cl_3_N_7_O_6_PtS^+^] 917.17 found 918.13. Elemental analysis calculated for [C_29_H_42_Cl_3_N_7_O_6_PtS·3CF_3_COOH·H_2_O] C, 32.89; H, 3.71; N, 7.67; Observed: C, 32.65; H, 3.87; N, 7.95.

#### *ctc*-[Pt­(NH_3_)_2_(ceritinib)­(PhB)­Cl_2_] (**7**)

Yield: 49%. RP-HPLC (analytical): RT= 6.25 min. ^1^H NMR (500 MHz, DMSO) δ (ppm): 9.55 (s, 1H), 8.42 (d, *J* = 8.1 Hz, 1H), 8.27 (s, 1H), 8.21 (s, 1H), 7.84 (dd, *J* = 8.0, 1.5 Hz, 1H), 7.64 (t, *J* = 7.8 Hz, 1H), 7.46 (s, 1H), 7.41–7.36 (m, 1H), 7.30–7.24 (m, 2H), 7.24–7.14 (m, 3H), 6.80 (s, 1H), 6.65 (s, 6H), 4.58–4.51 (m, 2H), 4.21 (s, 2H), 3.43 (dd, *J* = 13.6, 6.8 Hz, 1H), 2.77 (dd, *J* = 24.2, 12.4 Hz, 3H), 2.59 (t, *J* = 7.6 Hz, 2H), 2.24 (t, *J* = 7.4 Hz, 2H), 2.13 (s, 3H), 1.81–1.69 (m, 2H), 1.59 (s, 4H), 1.21 (d, *J* = 6.0 Hz, 6H), 1.15 (d, *J* = 6.8 Hz, 6H). ^195^Pt NMR: 107.5 MHz, DMSO, 298 K: δ (ppm) 1232. ESI-MS (+ve mode): *m*/*z* calculated for [C_39_H_53_Cl_3_N_7_O_7_PtS^+^] 1063.24 found 1063.10. Elemental analysis calculated for [C_39_H_52_Cl_3_N_7_O_7_PtS·3CF_3_COOH] C, 38.43; H, 3.94; N, 6.97; Observed: C, 38.42; H, 4.01; N, 7.23.

#### *ctc*-[Pt­(DACH)­(ceritinib)­(PhB)­(Ox)] (**8**)

Yield: 47%. RP-HPLC (analytical): RT= 6.20 min. ^1^H NMR (500 MHz, DMSO) δ (ppm): 9.70 (s, 1H), 9.57 (s, 1H), 9.13 (s, 1H), 8.78 (s, 1H), 8.54 (s, 1H), 8.40 (d, *J* = 8.2 Hz, 1H), 8.36 (s, 1H), 8.30–8.13 (m, 3H), 7.85 (dd, *J* = 8.0, 1.5 Hz, 1H), 7.66 (t, *J* = 7.5 Hz, 1H), 7.46 (s, 1H), 7.40 (t, *J* = 7.6 Hz, 1H), 7.26 (dd, *J* = 9.9, 5.3 Hz, 2H), 7.17 (dd, *J* = 7.3, 5.8 Hz, 3H), 6.77 (s, 1H), 4.65 (s, 1H), 4.09 (s, 2H), 3.44 (dt, *J* = 13.6, 6.8 Hz, 1H), 2.89–2.72 (m, 3H), 2.68–2.51 (m, 4H), 2.29 (dd, *J* = 7.6, 5.8 Hz, 2H), 2.12 (d, *J* = 18.2 Hz, 5H), 1.80–1.70 (m, 2H), 1.65 – 1.30 (m, 8H), 1.22 (d, *J* = 6.0 Hz, 6H), 1.15 (d, *J* = 6.8 Hz, 8H). ^195^Pt NMR: 107.5 MHz, DMSO, 298 K: δ (ppm) 1593. ESI-MS (+ve mode): *m*/*z* calculated for [C_47_H_61_ClN_7_O_11_PtS ^+^] 1161.34 found 1162.58. Elemental analysis calculated for [C_47_H_60_ClN_7_O_11_PtS.3CF_3_COOH.MeCN] C, 42.76; H, 4.31; N, 7.25; Observed: C, 43.08; H, 4.36; N, 7.27.

### Stability of the Pt­(IV) Complexes in Cell Culture Media

Stability studies of Pt­(IV) complexes were performed in RPMI 1640 (no glutamine) cell culture media supplemented with 10% fetal bovine serum (FBS), gentamycin sulfate solution (0.1% v/v), and l-glutamine solution (final concentration: 2 mM) on a Thermo Scientific UltiMAte 3000 HPLC. The stock solutions were prepared by dissolving the compounds in a 1:9 (v/v) mixture of DMSO and RPMI media. After preparation, the samples were injected and stored at 37 °C throughout the experiment. The final concentration of the complexes used for the experiment is 50–60 μM. The column used for the experiment is LC XB-C18 column (Phenomenex Kinetex, length 100 mm, internal diameter 4.60 mm, particle size 2.6 μm, and pore size 100 Å), and the method was set at flow rate 1.0 mL min, wavelength 260 nm (UV detector), 1.84 min 0.1% trifluoroacetic acid in water (100%) to equilibrate, in 5.84 min from 0 to 100% acetonitrile, and 2 min 100% acetonitrile.

### Reduction of Platinum Complexes by Ascorbic Acid Using RP-HPLC

The reduction of the Pt­(IV) complexes was performed in the presence of 10 equiv of l-ascorbic acid (AA) in phosphate buffer (100 mM, pH 7.4) at 37 °C using reverse-phase analytical HPLC. The final concentration of the Pt­(IV) complexes used in each experiment is 400–450 μM (100 μM for complex **7** due to poor solubility in phosphate buffer). The platinum complexes were dissolved in 10% DMSO with 50% MeOH and added to 100 mM phosphate buffer (pH 7.4). The mixture was then combined with an ascorbic acid solution at a final concentration of 4–4.5 mM and 1 mM for complex **7** (10 equiv of the Pt­(IV) complexes). The samples were injected and stored at 37 °C throughout the experiment. The column used for the experiment is LC XB-C18 column (Phenomenex Kinetex, length 100 mm, internal diameter 4.60 mm, particle size 2.6 μm, and pore size 100 Å), and the method was set at flow rate 1.0 mL min, wavelength 260 nm (UV detector), 1.84 min 0.1% trifluoroacetic acid in water (100%) to equilibrate, in 5.84 min from 0 to 100% acetonitrile, and 2 min 100% acetonitrile.

### Cell Lines

Human lung carcinoma (A549), human nonsmall cell lung carcinoma (NCI-H2228), human glioblastoma (U-87 MG), and human rhabdomyosarcoma (RD) cells were purchased from ATCC (Manassas, VA, USA), human colorectal carcinoma (HCT116), human breast carcinoma (MDA-MB-231), normal human lung fibroblasts (MRC-5), murine ovary carcinoma (CT26) and murine macrophages (J774.A1) were obtained from ECACC (Salisbury, UK). Immortalized epithelial pancreatic cell line (hTERT-HPNE), human fetal lung fibroblast cell line (IMR-90), and the epithelial cell line from mammary gland (MCF 10A) were purchased from ATCC. A pair of Chinese hamster ovary cell lines (CHOK1­(WT) and MMC2­(NER-deficient)) was kindly provided by Dr. Pirsel, Cancer Research Institute, Slovak Academy of Sciences (Bratislava, Slovakia). CT26 and NCI-H2228 cells were cultured in RPMI 1640 (Biosera, Boussens, France), and the remaining cell lines were cultured in DMEM medium (high glucose, 4.5 mg mL^–1^, Serva, Heidelberg, Germany). Both media were supplemented with gentamycin (50 mg mL^–1^, Serva, Heidelberg, Germany) and 10% heat-inactivated fetal bovine serum (PAA, Pasching, Austria). The medium for MRC-5 cells was further supplemented with 1% nonessential amino acids (Sigma-Aldrich, Prague, Czech Republic), and the medium for NCI-H2228 was supplemented with 1× GlutaMAX (Gibco). The cells were incubated in a humidified atmosphere containing 5% CO_2_ at 37 °C.

### Cytotoxic/Antiproliferative Activity

The cells were seeded on a 96-well plate at a proper density (1 × 10^3^ cells/well (HCT116), 2 × 10^3^ cells/well (RD, A549, U87-MG, CHOK1, MMC2), and 3 × 10^3^ (NCI-H2228, MDA-MB-231, MRC-5) and grown overnight. The cells were then treated with the tested compounds at various concentrations. In case of Pt­(IV) complexes the concentration were calculated using the molecular weight that include the trifluoroacetic acid and the solvent molecules as per the elemental analysis. After a 72 h treatment, MTT was added to the final concentration of 0.125 mg mL^–1^ and incubated with the cells for 3–4 h. MTT metabolization products, formazans, were dissolved in DMSO, and the resulting coloring was evaluated spectrophotometrically (570 nm vs reference 620 nm). IC_50_ values were calculated as compound concentrations corresponding to 50% inhibition of absorbance (vs control). Cytotoxicity in hepatocyte-like cells (HLC). STEMdiff Hepatocyte Kit was used to generate HLC from human pluripotent stem cells. Cytotoxicity of several of the tested compounds in HLC was evaluated using the sulphorhodamine B assay.

### Platination of DNA

NCI-H2228 cells were seeded in Petri dishes at a density of 2 × 10^6^ cells/dish and incubated overnight. The cells were then treated with the tested compounds at 5 μM concentration for 4 h. The cells were then harvested with trypsin, pelleted, and DNA was isolated using the DNAzol reagent following the manufacturer’s instructions. DNA concentration was then determined spectrophotometrically, and Pt content was measured with ICP-MS.

### Phosphorylation of H2AX

NCI-H2228 cells were seeded in dishes suitable for confocal microscopy (MatTEK, glass-bottom, 35 mm) at a density of 1 × 10^5^ cells/dish, incubated overnight, and treated with the tested compounds for 24 h. The cells were then fixed with methanol, blocked, and incubated with a primary anti-γH2AX antibody followed by incubation with a secondary AlexaFluor 488-conjugated antibody. The nuclei were counter-stained with DAPI. The samples were mounted with ProLong Diamond antifade mountant, and the images were recorded on a Leica SP8 confocal microscope.

### Cytotoxicity in 3D Spheroids

A549 and NCI-H2228 cells were seeded in ultralow attachment, U-shape plates (Corning) at a density of 800 cells/well in the tumorsphere medium consisting of DMEM/F12 medium (Sigma), fibroblast growth factor (20 ng/mL; Sigma), epidermal growth factor 2 (10 ng mL^–1^; Sigma), B27 supplement (1×; Gibco), bovine serum albumin, bovine serum albumin (BSA) (1.5 mg mL^–1^; Sigma). Four days after seeding, the spheroids were treated with a series of compound concentrations and incubated for an additional 96 h. CellTiter-Glo 3D viability assay was used for the experiment evaluation following the manufacturer’s instructions. The bioluminescence signal, proportional to the number of living cells, was measured using the luminescence reader SPARK, and IC_50_ values were calculated.

### Cellular Accumulation

A549 and NCI-H2228 cells were seeded in Petri dishes at a density of 2 × 10^6^ cells/dish and incubated overnight. The next day, the cells were treated with 1 μM compounds for 4 h. The cells were then harvested with trypsin, washed, counted, pelleted, and lysed in conc HCl for 4 days. The platinum amount in the lysates was determined using ICP-MS. Typically 2.5–3.5 × 10^6^ cells were analyzed in one sample. The results are expressed as MEAN ± SD from two independent measurements.

### Effect on Cell Invasiveness

#### Scratch Test

A549 cells were seeded in 24-well plates with inserts (Ibidi) at a density of 2 × 10^4^ cells/chamber and incubated overnight. The inserts were then withdrawn, and fresh medium containing the investigated compounds was added. The image of the cell-free region was recorded at the time of compound addition and then after 48 h. The cell-free area was evaluated with Fiji software.

#### Spheroid Outgrowing into the Matrigel

A549 and NCI-H2228 spheroids were grown as in the experiment above and embedded in Corning Matrigel Basement Membrane Matrix hESC (50 μL). Tumor sphere medium containing the investigated compounds at concentrations corresponding to IC_50_ values was added, and the spheroids were cultured for an additional 72 h. The images were recorded after 72 h.

### Cell Cycle Modulation

NCI-H2228 cells were seeded in 6-well plates (2.5 × 10^5^ cells/well), grown for 24 h, and then treated with the investigated compounds at concentrations corresponding to 2 × IC_50_ values. Following 48 h of incubation, the cells were harvested and fixed with 70% ethanol. The next day, the cells were washed with PBS (2×) and stained with propidium iodide (PI) (50 μg mL^–1^ with 100 μg mL^–1^ RNase A) in Vindel’s solution (10 mM Tris-Cl (pH = 8.0), 10 mM NaCl, 0.1% Triton X-100) for 30 min at 37 °C. Cell cycle distribution was analyzed using flow cytometry (FACS Verse), 3 × 10^4^ cells were analyzed following the exclusion of cell aggregates, and the data was analyzed with FCS Express 7 (DeNovo software, Glendale, CA). The results are expressed as MEAN from two experiments.

### Detection of Cell Death Mode

NCI-H2228 cells were seeded in 6-well plates (2.5 × 10^5^ cells/well), grown for 24 h, and then treated with the investigated compounds at concentrations corresponding to 3 × IC_50_ values for 48 h. Staurosporine (4 μM; 2 h) was used as a positive control of apoptosis. The cells were then harvested with trypsin and stained with Annexin V-Pacific Blue conjugate (1:20 dilution) and PI (10 μg mL^–1^) for 15 min. The samples were analyzed with flow cytometry (FACS Verse), 3 × 10^4^ cells were analyzed following the exclusion of cell aggregates, and the data was analyzed with FCS Express 7 (DeNovo software, Glendale, CA).

### Western Blot

NCI-H2228 cells were seeded in 6-well plates at a density of 2 × 10^5^ cells/well and incubated overnight. The cells were then treated with the tested compounds at concentrations corresponding to their respective IC_50_ values for 24 h. The cells were then harvested and lysed using RIPA lysis buffer supplemented with protease and phosphatase inhibitors as recommended by the manufacturer. Protein extracts were resolved with SDS-PAGE, transferred to a PVDF membrane, and the respective proteins were detected with specific antibodies (anti-phosphoALK, anti-ALK, anti-phosphoMET, anti-MET, and anti-GADPH) and HRP-conjugated secondary antibodies. The signals were obtained with SignalFire ECL Reagent and recorded with an Amersham680 reader.

### Immunogenic Cell Death

#### Externalization of Calreticulin. Detection of Calreticulin Externalization with Flow Cytometry

CT26 cells were seeded in 6-well plates at a density of 2.5 × 10^5^ cells/well and grown overnight. The cells were then treated with the investigated compounds at their equitoxic concentrations corresponding to IC_50_ values for 16 h. The cells were then harvested, fixed with 4% paraformaldehyde, washed thoroughly, and stained with an anti-Calreticulin–AlexaFluor 488 conjugated antibody (Abcam) at 4 °C overnight. PI was added before the flow cytometry analysis with a BD FACS Verse flow cytometer. Then 3 × 10^4^ cells were analyzed following the exclusion of cell aggregates. Data was analyzed with FSC Express 7 software (DeNovo software, Glendale, CA).

#### Detection of Calreticulin Translocation with a Confocal Microscope

NCI-H2228 cells were seeded on 2 cm × 2 cm coverslips in 6-well plates at a density of 2 × 10^5^ cells/well and grown overnight. The cells were then treated with the investigated compounds at their equitoxic concentrations corresponding to IC_50_ values for 16 h. The cells were then fixed with methanol, washed appropriately, permeabilized with 0.1% Triton X-100 in PBS, blocked with 3% fetal bovine serum (FBS), and stained with an anticalreticulin antibody (Abcam) at 4 °C overnight. Following washing, the antirabbit AlexaFluor 488-conjugated antibody (Abcam) was added and incubated with the cells for 2 h. The cells were then washed and mounted with ProLong Diamond Antifade (Invitrogen). The images were captured using a Leica SP5 Confocal Microscope.

### Release of ATP

CT26 and NCI-H2228 cells were seeded in 48-well plates at a density of 5 × 10^4^ cells/well and grown overnight. The next day, the cells were treated with equitoxic concentrations of the compounds corresponding to IC_50_ values and incubated for 20 h. Then 50 μL of supernatant from the wells was transferred into a 96-well white plate (Corning) and processed using an ATP bioluminescence assay kit CLSII following the manufacturer’s instructions. Luminescence was measured with a SPARK multimode reader (TECAN).

### Detection of Extracellular HMGB1

CT26 and NCI-H2228 cells were seeded in 48-well plates at a density of 5 × 10^4^ cells/well and grown overnight. The next day, the cells were treated with equitoxic concentrations of the compounds corresponding to IC_50_ values and incubated for 24 h. Aliquots of the supernatants were then withdrawn and processed using the HMGB1 Express ELISA kit (TECAN) according to the manufacturer’s instructions. The final absorbance, proportional to the HMGB1 amount, was read at 420 nm using a multimode SPARK reader (TECAN).

### Detection of Phagocytosis

CT26 cells were seeded at a density of 2 × 10^5^ cells/well in 6-well plates and incubated for 24 h. The cells were then treated with the compounds at concentrations corresponding to IC_50_ values and incubated for an additional 24 h. Then, the cells in the samples were stained with CellTracker (red CMTPX; Thermo Fisher Scientific). At the same time, J774.A1 macrophages were stained with CellTracker (green CMFDA; ThermoFisher Scientific). The cells were washed and coincubated at a ratio of J774.A1:CT26 of 1:2 for 4 h. Following the coincubation, the cells were harvested and analyzed with flow cytometry (BD FACS Verse). Total cell count was normalized to 3 × 10^4^ macrophages. The data were analyzed with FCS Express 7 (DeNovo software; Glendale, CA).

## Supplementary Material





## Notes

NMR spectra, HPLC chromatograms, and ESI-MS spectra of the investigated complexes; synthetic approach to the preparation of complexes **2**–**4** and **6**–**8**; stability half-lives (*t*_1/2_) of **2**, **3**, **7** and **8**; percentage of intact complexes **4** and **8** in different time intervals; stability in PBS; reduction of **2**, **4**, **6**, **7**, and **8** in the presence of ascorbic acid; reduction of **2** and **6** by ESI-MS; DNA platination; DNA damage; scratch test; spheroid outgrowing into the Matrigel; cell cycle and cell death; Western blots; cytotoxic/antiproliferative activity of selected compounds in CT26 cell line; calreticulin exposure to the cell membrane; phagocytosis (PDF)
Molecular formula strings and biological data (CSV)

## Abbreviations Used

2D: two-dimensional
3D: three-dimensional
AA: l-ascorbic acid
ALK: anaplastic lymphoma kinase
BSA: bovine serum albumin
DAMP: damage-associated molecular pattern
DSC: *N*,*N*′-disuccinylcarbonate
ELISA: enzyme-linked immunosorbent assay
FBS: fetal bovine serum
IC_50_: concentration of the agent inhibiting cell growth by 50%
ICD: immunogenic cell death
ICP-MS: inductively coupled plasma mass spectrometry
MOA: mechanism of action
MTT: 3-[4,5-dimethylthiazol-2-yl]-2,5 diphenyl tetrazolium bromide
NER: nucleotide excision repair
NSCLC: nonsmall cell lung cancer
PBS: phosphate buffered saline
PI: propidium iodide
RD: human rhabdomyosarcoma
RPMI: Roswell Park Memorial Institute
SD: standard deviation
RT: retention time
SI: selectivity index
*t*_1/2_: half-life
TFA: trifluoroacetic acid

## References

[1] Zhang C., Xu C., Gao X., Yao Q. (2022). Platinum-based drugs for cancer therapy
and anti-tumor strategies. Theranostics.

[2] Wang D., Lippard S. J. (2005). Cellular processing
of platinum anticancer drugs. Nature Rev. Drug
Discovery.

[3] Gibson D. (2009). The mechanism
of action of platinum anticancer agents—what do we really know
about it?. Dalton Trans..

[4] Brabec V., Hrabina O., Kasparkova J. (2017). Cytotoxic platinum coordination compounds.
DNA binding agents. Coord. Chem. Rev..

[5] Oun R., Moussa Y. E., Wheate N. J. (2018). The side
effects of platinum-based
chemotherapy drugs: a review for chemists. Dalton
Trans..

[6] Gibson D. (2019). Multi-action
Pt­(IV) anticancer agents; do we understand how they work?. J. Inorg. Biochem..

[7] Gibson D. (2016). Platinum­(IV)
anticancer prodrugs - hypotheses and facts. Dalton Trans..

[8] Xu Z., Wang Z., Deng Z., Zhu G. (2021). Recent advances in
the synthesis, stability, and activation of platinum­(IV) anticancer
prodrugs. Coord. Chem. Rev..

[9] Li G., Che X., Wang S., Liu D., Xie D., Jiang B., Zheng Z., Zheng X., Wu G. (2025). The role of cisplatin
in modulating the tumor immune microenvironment and its combination
therapy strategies: a new approach to enhance anti-tumor efficacy. Annals of medicine.

[10] Yu C., Wang Z., Sun Z., Zhang L., Zhang W., Xu Y., Zhang J.-J. (2020). Platinum-based combination therapy: Molecular rationale,
current clinical uses, and future perspectives. J. Med. Chem..

[11] Raveendran R., Braude J. P., Wexselblatt E., Novohradsky V., Stuchlikova O., Brabec V., Gandin V., Gibson D. (2016). Pt­(IV) derivatives
of cisplatin and oxaliplatin with phenylbutyrate axial ligands are
potent cytotoxic agents that act by several mechanisms of action. Chem. Sci..

[12] Li X., Liu Y., Tian H. (2018). Current developments in Pt­(IV) prodrugs conjugated
with bioactive ligands. Bioinorg. Chem. Appl..

[13] Navas F., Chocarro-Calvo A., Iglesias-Hernández P., Fernández-García P., Morales V., García-Martínez J. M., Sanz R., De la Vieja A., García-Jiménez C., García-Muñoz R. A. (2024). Promising anticancer prodrugs based
on Pt­(IV) complexes with bis-organosilane ligands in axial positions. J. Med. Chem..

[14] Fronik P., Poetsch I., Kastner A., Mendrina T., Hager S., Hohenwallner K., Schueffl H., Herndler-Brandstetter D., Koellensperger G., Rampler E., Kopecka J., Riganti C., Berger W., Keppler B. K., Heffeter P., Kowol C. R. (2021). Structure–activity
relationships of triple-action platinum­(IV) prodrugs with albumin-binding
properties and immunomodulating ligands. J.
Med. Chem..

[15] Spector D., Zharova A., Bykusov V., Karetnikov G., Beloglazkina E., Krasnovskaya O. (2025). Recent advances in antitumor Pt­(IV)
complexes: Dual targeting and chemoimmunotherapy. Coord. Chem. Rev..

[16] Xu L., Kong X., Li X., Zhang B., Deng Y., Wang J., Duan C., Zhang D., Liu W. (2024). Current status
of novel multifunctional targeted Pt­(IV) compounds and their reductive
release properties. Molecules (Basel, Switzerland).

[17] Stinchcombe T. E., Borghaei H., Barker S. S., Treat J. A., Obasaju C. (2016). Pemetrexed
with platinum combination as a backbone for targeted therapy in non-small-cell
lung cancer. Clin. Lung Cancer.

[18] Xiao H. Q., Tian R. H., Zhang Z. H., Du K. Q., Ni Y. M. (2016). Efficacy
of pemetrexed plus platinum doublet chemotherapy as first-line treatment
for advanced nonsquamous non-small-cell-lung cancer: a systematic
review and meta-analysis. OncoTargets Ther..

[19] Soria J. C., Tan D. S. W., Chiari R., Wu Y. L., Paz-Ares L., Wolf J., Geater S. L., Orlov S., Cortinovis D., Yu C. J., Hochmair M., Cortot A. B., Tsai C. M., Moro-Sibilot D., Campelo R. G., McCulloch T., Sen P., Dugan M., Pantano S., Branle F., Massacesi C., de Castro G. (2017). First-line ceritinib versus platinum-based
chemotherapy in advanced ALK-rearranged non-small-cell lung cancer
(ASCEND-4): a randomised, open-label, phase 3 study. Lancet.

[20] Hallberg B., Palmer R. H. (2013). Mechanistic insight into ALK receptor
tyrosine kinase
in human cancer biology. Nature Rev. Cancer.

[21] Gandhi S., Chen H., Zhao Y., Dy G. K. (2015). First-line treatment
of advanced ALK-positive non-small-cell lung cancer. Lung Cancer (Auckland, N.Z.).

[22] Petrazzuolo A., Perez-Lanzon M., Liu P., Maiuri M. C., Kroemer G. (2021). Crizotinib
and ceritinib trigger immunogenic cell death via on-target effects. Oncoimmunology.

[23] Babu T., Sarkar A., Karmakar S., Schmidt C., Gibson D. (2020). Multiaction
Pt­(IV) carbamate complexes can codeliver Pt­(II) drugs and amine containing
bioactive molecules. Inorg. Chem..

[24] Zheng Y. R., Suntharalingam K., Johnstone T. C., Yoo H., Lin W., Brooks J. G., Lippard S. J. (2014). Pt­(IV) prodrugs designed to bind
non-covalently to human serum albumin for drug delivery. J. Am. Chem. Soc..

[25] Schueffl H., Theiner S., Hermann G., Mayr J., Fronik P., Groza D., van Schonhooven S., Galvez L., Sommerfeld N. S., Schintlmeister A., Reipert S., Wagner M., Mader R. M., Koellensperger G., Keppler B. K., Berger W., Kowol C. R., Legin A., Heffeter P. (2021). Albumin-targeting of an oxaliplatin-releasing
platinum­(iv) prodrug results in pronounced anticancer activity due
to endocytotic drug uptake in vivo. Chem. Sci..

[26] Theiner S., Grabarics M., Galvez L., Varbanov H. P., Sommerfeld N. S., Galanski M. S., Keppler B. K., Koellensperger G. (2018). The impact
of whole human blood on the kinetic inertness of platinum­(iv) prodrugs
- an HPLC-ICP-MS study. Dalton Trans..

[27] Kostrhunova H., Petruzzella E., Gibson D., Kasparkova J., Brabec V. (2019). A new anticancer Pt­(IV)
prodrug that acts by mechanisms
involving DNA damage and different epigenetic effects. Chem.—Eur. J..

[28] Wexselblatt E., Gibson D. (2012). What do we know about the reduction of Pt­(IV) pro-drugs?. J. Inorg. Biochem..

[29] Friboulet L., Li N., Katayama R., Lee C. C., Gainor J. F., Crystal A. S., Michellys P. Y., Awad M. M., Yanagitani N., Kim S., Pferdekamper A. C., Li J., Kasibhatla S., Sun F., Sun X., Hua S., McNamara P., Mahmood S., Lockerman E. L., Fujita N., Nishio M., Harris J. L., Shaw A. T., Engelman J. A. (2014). The ALK inhibitor ceritinib overcomes
crizotinib resistance in non-small cell lung cancer. Cancer Discovery.

[30] Huang J., Zhao Y., Xu Y., Zhu Y., Huang J., Liu Y., Zhao L., Li Z., Liu H., Wang Q. L., Qi X. (2016). Comparative effectiveness and safety
between oxaliplatin-based and
cisplatin-based therapy in advanced gastric cancer: A meta-analysis
of randomized controlled trials. Oncotarget.

[31] Kollmannsberger C., Rick O., Derigs H.-G., Schleucher N., Schöffski P., Beyer J., Schoch R., Sayer H. G., Gerl A., Kuczyk M., Spott C., Kanz L., Bokemeyer C. (2002). Activity of oxaliplatin in patients
with relapsed or
cisplatin-refractory germ cell cancer: A study of the German testicular
cancer study group. J. Clin. Oncol..

[32] Atmaca A., Al-Batran S. E., Werner D., Pauligk C., Güner T., Koepke A., Bernhard H., Wenzel T., Banat A. G., Brueck P., Caca K., Prasnikar N., Kullmann F., Günther Derigs H., Koenigsmann M., Dingeldein G., Neuhaus T., Jäger E. (2013). A randomised
multicentre phase II study with cisplatin/docetaxel vs oxaliplatin/docetaxel
as first-line therapy in patients with advanced or metastatic non-small
cell lung cancer. Br. J. Cancer.

[33] Mah L. J., El-Osta A., Karagiannis T. C. (2010). gH2AX:
a sensitive molecular marker
of DNA damage and repair. Leukemia.

[34] Mayr J., Heffeter P., Groza D., Galvez L., Koellensperger G., Roller A., Alte B., Haider M., Berger W., Kowol C. R., Keppler B. K. (2017). An albumin-based
tumor-targeted oxaliplatin
prodrug with distinctly improved anticancer activity in vivo. Chem. Sci..

[35] Frensemeier L. M., Mayr J., Koellensperger G., Keppler B. K., Kowol C. R., Karst U. (2018). Structure elucidation and quantification of the reduction products
of anticancer Pt­(iv) prodrugs by electrochemistry/mass spectrometry
(EC-MS). Analyst.

[36] Riddell, I. A. ; Lippard, S. J. Cisplatin and oxaliplatin: Our current understanding of their actions. In Metallo-Drugs: Development and Action of Anticancer Agents, Sigel, A. , Sigel, H. , Freisinger, E. , Sigel, R. K. O. , Eds.; De Gruyter 2018; pp 1–42.

[37] Park S., Cho E. A., Chun J. N., Lee D. Y., Lee S., Kim M. Y., Bae S. M., Jo S. I., Lee S. H., Park H. H., Kim T. M., So I., Kim S.-Y., Jeon J.-H. (2022). Crizotinib attenuates cancer metastasis
by inhibiting
TGFβ signaling in non-small cell lung cancer cells. Exp. Mol. Med..

[38] Xu X., Yang G., Shi N. (2025). Ceritinib reduces transendothelial
invasion of non-small cell lung cancer cells by restoring claudin-10
and suppressing VEGF-A signaling. Biochem. Genet..

[39] Boulos J. C., Saeed M. E. M., Chatterjee M., Bülbül Y., Crudo F., Marko D., Munder M., Klauck S. M., Efferth T. (2021). Repurposing of the ALK inhibitor crizotinib for acute
leukemia and multiple myeloma cells. Pharmaceuticals.

[40] Megiorni F., McDowell H. P., Camero S., Mannarino O., Ceccarelli S., Paiano M., Losty P. D., Pizer B., Shukla R., Pizzuti A., Clerico A., Dominici C. (2015). Crizotinib-induced
antitumour activity in human alveolar rhabdomyosarcoma cells is not
solely dependent on ALK and MET inhibition. J. Exp. Clin. Cancer Res..

[41] Subbiah V., Kuravi S., Ganguly S., Welch D. R., Vivian C. J., Mushtaq M. U., Hegde A., Iyer S., Behrang A., Ali S. M., Madison R. W., Venstrom J. M., Jensen R. A., McGuirk J. P., Amin H. M., Balusu R. (2021). Precision therapy with
anaplastic lymphoma kinase inhibitor ceritinib in ALK-rearranged anaplastic
large cell lymphoma. ESMO open.

[42] Nagata S., Suzuki J., Segawa K., Fujii T. (2016). Exposure of phosphatidylserine
on the cell surface. Cell Death Differ..

[43] Liu P., Zhao L., Pol J., Levesque S., Petrazzuolo A., Pfirschke C., Engblom C., Rickelt S., Yamazaki T., Iribarren K., Senovilla L., Bezu L., Vacchelli E., Sica V., Melis A., Martin T., Xia L., Yang H., Li Q., Chen J., Durand S., Aprahamian F., Lefevre D., Broutin S., Paci A., Bongers A., Minard-Colin V., Tartour E., Zitvogel L., Apetoh L., Ma Y., Pittet M. J., Kepp O., Kroemer G. (2019). Crizotinib-induced immunogenic cell death in non-small
cell lung cancer. Nature Commun..

[44] Petrazzuolo A., Perez-Lanzon M., Liu P., Maiuri M. C., Kroemer G. (2021). Crizotinib
and ceritinib trigger immunogenic cell death via on-target effects. Oncoimmunol..

[45] Zhou J., Wang G., Chen Y., Wang H., Hua Y., Cai Z. (2019). Immunogenic cell death in cancer therapy: Present and
emerging inducers. J. Cell. Mol. Med..

[46] Obeid M., Tesniere A., Ghiringhelli F., Fimia G. M., Apetoh L., Perfettini J.-L., Castedo M., Mignot G., Panaretakis T., Casares N., Métivier D., Larochette N., van Endert P., Ciccosanti F., Piacentini M., Zitvogel L., Kroemer G. (2007). Calreticulin exposure dictates the
immunogenicity of cancer cell death. Nature
Med..

[47] Bianchi M.
E. (2007). DAMPs,
PAMPs and alarmins: all we need to know about danger. Journal of leukocyte biology.

[48] Michaud M., Martins I., Sukkurwala A. Q., Adjemian S., Ma Y., Pellegatti P., Shen S., Kepp O., Scoazec M., Mignot G., Rello-Varona S., Tailler M., Menger L., Vacchelli E., Galluzzi L., Ghiringhelli F., di Virgilio F., Zitvogel L., Kroemer G. (2011). Autophagy-dependent
anticancer immune responses Induced by chemotherapeutic agents in
mice. Science.

[49] Garg A. D., Krysko D. V., Verfaillie T., Kaczmarek A., Ferreira G. B., Marysael T., Rubio N., Firczuk M., Mathieu C., Roebroek A. J., Annaert W., Golab J., de Witte P., Vandenabeele P., Agostinis P. (2012). A novel pathway
combining calreticulin exposure and ATP secretion in immunogenic cancer
cell death. EMBO J..

[50] Apetoh L., Ghiringhelli F., Tesniere A., Obeid M., Ortiz C., Criollo A., Mignot G., Maiuri M. C., Ullrich E., Saulnier P., Yang H., Amigorena S., Ryffel B., Barrat F. J., Saftig P., Levi F., Lidereau R., Nogues C., Mira J.-P., Chompret A., Joulin V., Clavel-Chapelon F., Bourhis J., André F., Delaloge S., Tursz T., Kroemer G., Zitvogel L. (2007). Toll-like
receptor 4–dependent contribution of the immune system to anticancer
chemotherapy and radiotherapy. Nature Med..

[51] Englinger B., Pirker C., Heffeter P., Terenzi A., Kowol C. R., Keppler B. K., Berger W. (2019). Metal drugs
and the anticancer immune
response. Chem. Rev..

