# Supplementary material for: Crizotinib- or Ceritinib-Conjugated Platinum(IV) Prodrugs As Potent Multiaction Agents Inducing Antiproliferative Effects in 2D and 3D Cancer Cell Models
Source: J Med Chem. 2025 Nov 10;68(22):24094–107. doi: 10.1021/acs.jmedchem.5c01858 (PMC12670396; doi:10.1021/acs.jmedchem.5c01858)
Supplement: Supplementary file 1 [file jm5c01858_si_001.pdf]

Supporting Information

Crizotinib- or ceritinib-conjugated platinum(IV) prodrugs as potent multi-action agents inducing antiproliferative effects in 2D and 3D cancer cell models

Sofia Sharkawy,[#ab] Sourav Acharya,[#c] Hana Kostrhunová,[b] Moumita Maji,[c] Lenka Marková,[b] Vojtěch Novohradský,[b] Dan Gibson[*c] and Viktor Brabec[*d]

[a]*Faculty of Science, Department of Biochemistry, Masaryk University, CZ-62500 Brno, Czech Republic*
[b]*Czech Academy of Sciences, Institute of Biophysics, Kralovopolska 135, CZ-61200 Brno, Czech Republic*
[c]*Institute for Drug Research, School of Pharmacy, The Hebrew University of Jerusalem, Jerusalem-9112102, Israel*
[d]*Department of Biophysics, Faculty of Science, Palacky University, Slechtitelu 27, 783 71 Olomouc, Czech Republic*

[#]S.S. and S.A. contributed equally to this work.
*Corresponding authors' e-mail addresses:
e-mail: dang@ekmd.huji.ac.il (D. Gibson); vbrabec44@gmail.com (V. Brabec)

Table of Contents

Figure S1. HPLC chromatogram of complex **2** ran with 0 – 100% linear gradient of acetonitrile in 0.1% TFA in water over 5.84 min + 2 min constant 100% acetonitrile 4

Figure S2. ^{195}Pt NMR of *ctc*-[Pt(NH_3)$_2$(crizotinib)(OH)Cl_2] (**2**) in DMSO-d_6............ 4

Figure S3. ^{195}Pt NMR of *ctc*-[Pt(NH_3)$_2$(crizotinib)(OH)Cl_2] (**2**) in DMSO-d_6............ 5

Figure S4. ESI-MS spectrum (+ve mode) of complex **2**............ 5

Figure S5. HPLC chromatogram of complex **3** ran with 0 – 100% linear gradient of acetonitrile in 0.1% TFA in water over 5.84 min + 2 min constant 100% acetonitrile 6

Figure S6. ^{1}H NMR of *ctc*-[Pt(NH_3)$_2$(crizotinib)(PhB)Cl_2] (**3**) in DMSO-d_6............ 6

Figure S7. ^{195}Pt NMR of *ctc*-[Pt(NH_3)$_2$(crizotinib)(PhB)Cl_2] (**3**) in DMSO-d6. 7

Figure S8. HSQC of *ctc*-[Pt(NH_3)$_2$(crizotinib)(PhB)Cl_2] (**3**) in DMSO-d_6. 7

Figure S9. ESI-MS spectrum (+ve mode) of complex **3**............ 8

Figure S10. HPLC chromatogram of complex **4** ran with 0 – 100% linear gradient of acetonitrile in 0.1% TFA in water over 5.84 min + 2 min constant 100% acetonitrile. 8

Figure S11. ^{1}H NMR of *ctc*-[Pt(DACH)(crizotinib)(PhB)(Ox)] (**4**) in DMSO-d_6. 9

Figure S12. ^{195}Pt NMR of ctc-[Pt(DACH)(crizotinib)(PhB)(Ox)] (**4**) in DMSO-d_6. 9

Figure S13. ESI-MS spectrum (+ve mode) of complex **4**. 10

Figure S15. ^{1}H NMR of ctc-[Pt(NH_3)$_2$(ceritinib)(OH)Cl_2] (**6**) in DMSO-d6. 11

Figure S16. ^{195}Pt NMR of ctc-[Pt(NH_3)$_2$(ceritinib)(OH)Cl_2] (**6**) in DMSO-d6. 11

Figure S17. ESI-MS spectrum (+ve mode) of complex **6**. 12

Figure S18. HPLC chromatogram of complex **7** ran with 0 – 100% linear gradient of acetonitrile in 0.1% TFA in water over 5.84 min + 2 min constant 100% acetonitrile 12

Figure S19. ^{1}H NMR of ctc-[Pt(NH_3)$_2$(ceritinib)(PhB)Cl_2] (**7**) in DMSO-d_6 13

Figure S20. ^{195}Pt NMR of ctc-[Pt(NH_3)$_2$(ceritinib)(PhB)Cl_2] (**7**) in DMSO-d6. 13

Figure S21. ESI-MS spectrum (+ve mode) of complex **7**. 14

Figure S22. HPLC chromatogram of complex **8** ran with 0 – 100% linear gradient of acetonitrile in 0.1% TFA in water over 5.84 min + 2 min constant 100% acetonitrile 14

Figure S23. ^{1}H NMR of ctc-[Pt(DACH)(ceritinib)(PhB)(Ox)] (**8**) in DMSO-d6. 15

Figure S24. ^{195}Pt NMR of ctc-[Pt(DACH)(ceritinib)(PhB)(Ox)] (**8**) in DMSO-d6. 15

Figure S25. ESI-MS spectrum (+ve mode) of complex **8**. 16

Scheme S1. Generalized synthetic approach to the preparation of complexes Pt(IV)-crizotinib (**2-4**) and Pt(IV)-ceritinib complexes (**6-8**) 16

Figure S26. Stability half-lives of complexes (A) **2**, (B) **3**, (C) **6** and (D) **7** in 10 % DMSO in RPMI media at 37 °C determined by HPLC. 17

Figure S27. Percentage of intact complexes **4** and **8** in different time intervals in 10 % DMSO in RPMI media at 37 °C. 17

Figure S28. Stability plot of the complexes at different time intervals in 10 % DMSO in PBS at 37 °C monitored by HPLC. 18

Figure S29. Reduction of **2** in the presence of 10 equiv. ascorbic acid taken at different time intervals in 100 mM phosphate buffer at pH 7.4 at 37 ºC 19

Figure S30. Reduction of **4** in the presence of 10 equiv. ascorbic acid taken at different time intervals in 100 mM phosphate buffer at pH 7.4 at 37 ºC. 19

Figure S31. Reduction of **6** in the presence of 10 equiv. ascorbic acid taken at different time intervals in 100 mM phosphate buffer at pH 7.4 at 37 ºC 20

Figure S32. Reduction of **7** in the presence of 10 equiv. ascorbic acid taken at different time intervals in 100 mM phosphate buffer at pH 7.4 at 37 ºC 20

Figure S33. Reduction of **8** in the presence of 10 equiv. ascorbic acid taken at different time intervals in 100 mM phosphate buffer at pH 7.4 at 37 ºC. 21

Figure S34. ESI-MS data for the reduction of **2** in the presence of 10 equiv. ascorbic acid in 100 mM phosphate buffer at pH 7.4 at 37 ºC after overnight incubation. 21

Figure S35. ESI-MS data for the reduction of **6** in the presence of 10 equiv. ascorbic acid in 100 mM phosphate buffer at pH 7.4 at 37 ºC after overnight incubation. 22

Figure S36. DNA platination. NCI-H2228 cells were exposed to 5µM complexes for 4 h. Cell pellets were lyzed and DNA was isolated with DNAzol. DNA concentration was determined spectrophotometrically and platinum content was determined with ICP-MS. The results are shown as MEAN±SD from two experiments. 22

Figure S37. DNA damage. H2AX phosphorylation (γH2AX foci) was used as the indicator of DNA damage in samples exposed to **3**, **7,** and cisplatin. NCI-H2228 cells were nontreated or treated with the tested compounds at concentrations corresponding to their respective 3xIC_{50} values for 24 hours. The cells were stained with an anti-γH2AX antibody and a secondary

AlexaFluor 488-conjugated antibody and counter-stained with DAPI. The images were recorded with a confocal microscope. 23

Figure S38. Scratch test. A549 cells were grown in 24-well plates with insert chambers (Ibidi). The inserts were then removed, and fresh medium containing the complexes at concentrations corresponding to IC_{50} values was added. The images were recorded immediately after the addition and then after 48 h. Representative images. 24

Figure S39. Spheroid outgrowing into the Matrigel. Top two rows - A549-derived spheroids. Bottom two rows – NCI-H2228 derived spheroids. The spheroids were grown for four days, then embedded in Matrigel and treated with the respective compounds at concentrations corresponding to their IC_{50} values. The images were recorded after 72 h of treatment. Representative images. 25

Figure S40. Cell cycle. Representative flow cytometry histograms of NCI-H2228 cells exposed to $2xIC_{50}$ concentrations of the investigated compounds for 48 h. Cell distribution into individual cell cycle phases was performed after staining with propidium iodide. Red – G1; Blue-striped – S; Green – G2/M. 26

Figure S41. Cell death. NCI-H2228 cells were exposed to the investigated compounds at concentrations corresponding to $3xIC_{50}$ values for 24 h. The cells were then stained with Annexin V/propidium iodide (PI), and the cell distribution into the quadrants is as follows: left-bottom: Annexin V+/PI- (living cells); right-bottom: Annexin V+/PI- (early apoptotic cells); left-top: Annexin V-/PI+ (early necrotic cells); right-top: Annexin V+/PI+ (late apoptotic/necrotic cells). STAU – Staurosporine-treated cells. 26

Figure S42. Western blot. Raw images for Figure 7. 27

Table S1. Cytotoxic/antiproliferative activity of selected compounds in CT26 cell line[ab] 28

Figure S43. Calreticulin exposure to the cell membrane. NCI-H2228 cells were treated with the compounds at concentrations corresponding to the IC_{50} values for 16 h. Following fixation, the cells were stained with a primary anti-calreticulin antibody and a secondary AlexaFluor 488-conjugated antibody. 28

Figure S44. Phagocytosis. CT26 cells were treated with the compounds at concentrations corresponding to IC50 values for 24 h. CT26 samples were then stained with CellTracker red, and J774.A1 macrophages with CellTracker green. The cancer cells were co-incubated with the macrophages for 4 h. The top panel shows the process of evaluating the results. Representative images 29

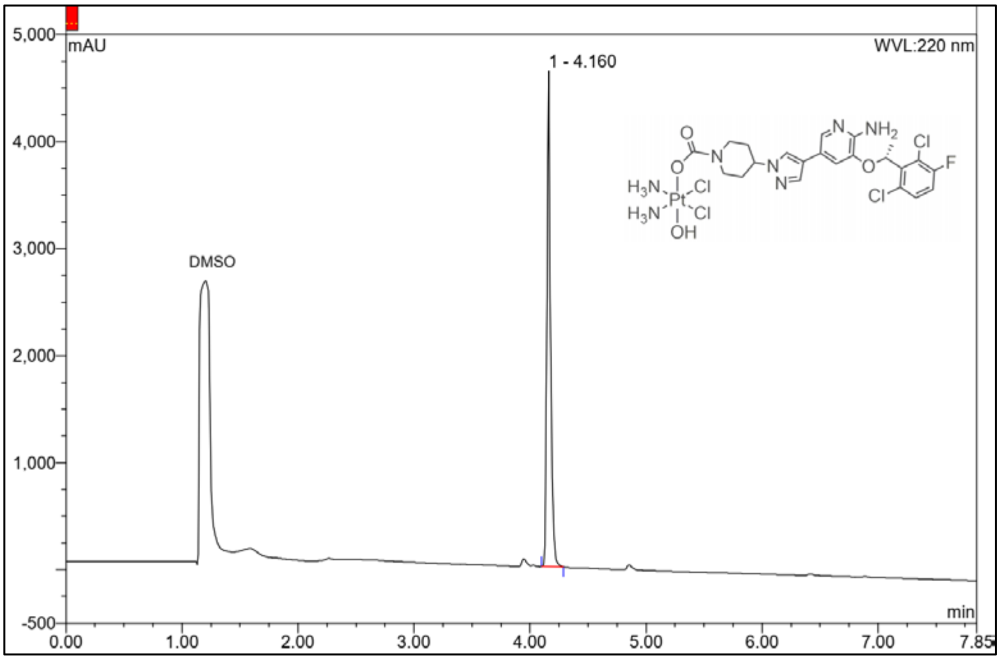

Figure S1. HPLC chromatogram of complex **2** ran with 0 – 100% linear gradient of acetonitrile in 0.1% TFA in water over 5.84 min + 2 min constant 100% acetonitrile

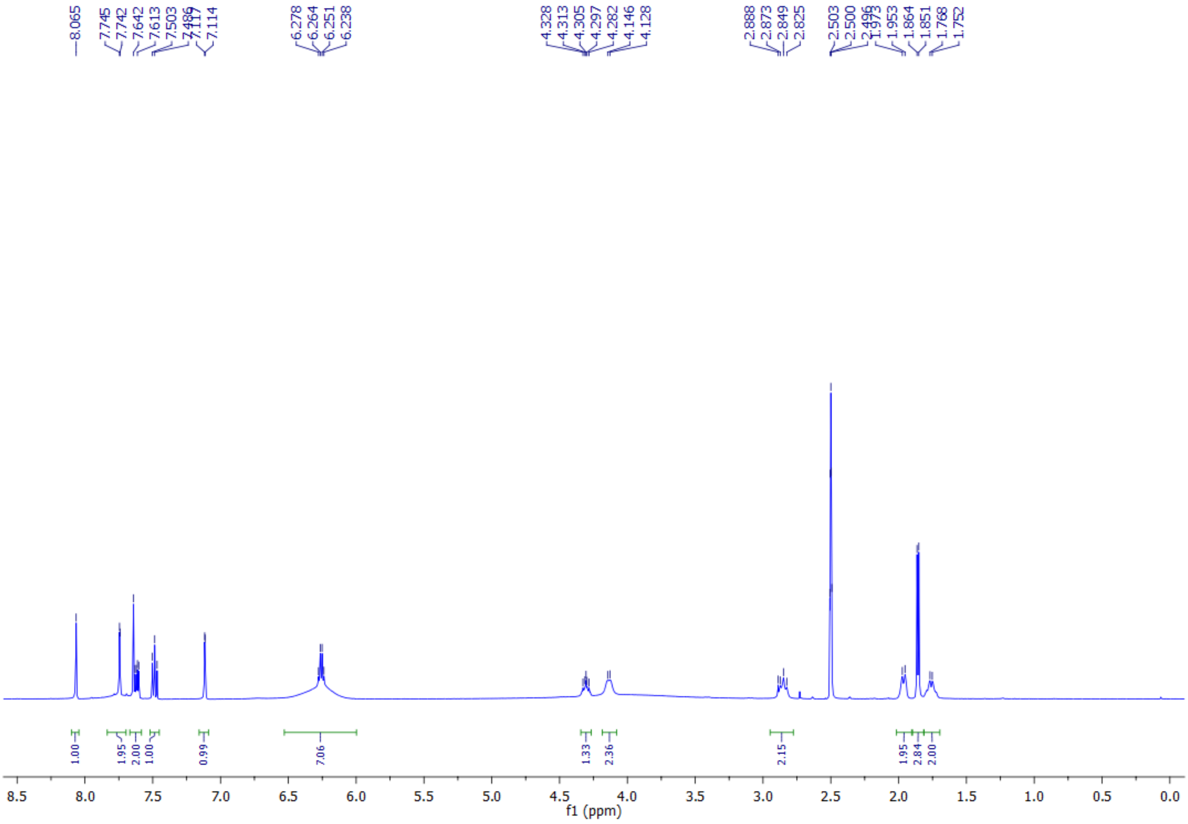

Figure S2. ^{195}Pt NMR of *ctc*-[Pt(NH_3)$_2$(crizotinib)(OH)Cl_2] (**2**) in DMSO-d_6.

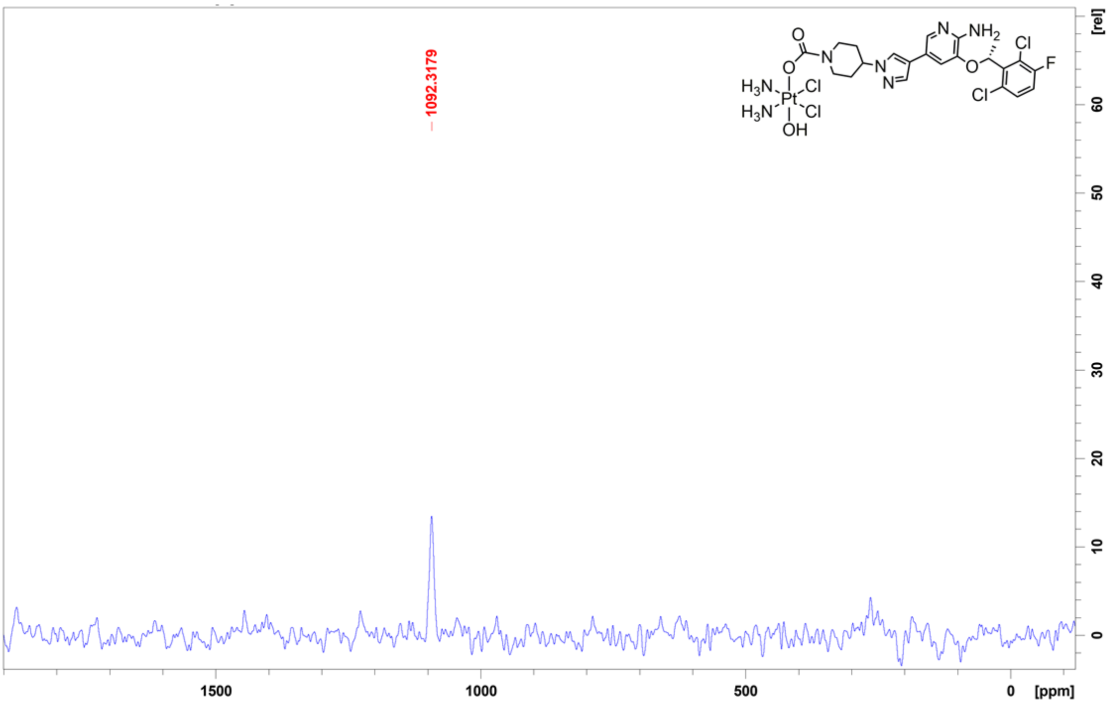

Figure S3. ^{195}Pt NMR of *ctc*-[Pt(NH_3)$_2$(crizotinib)(OH)Cl_2] (**2**) in DMSO-d$_6$.

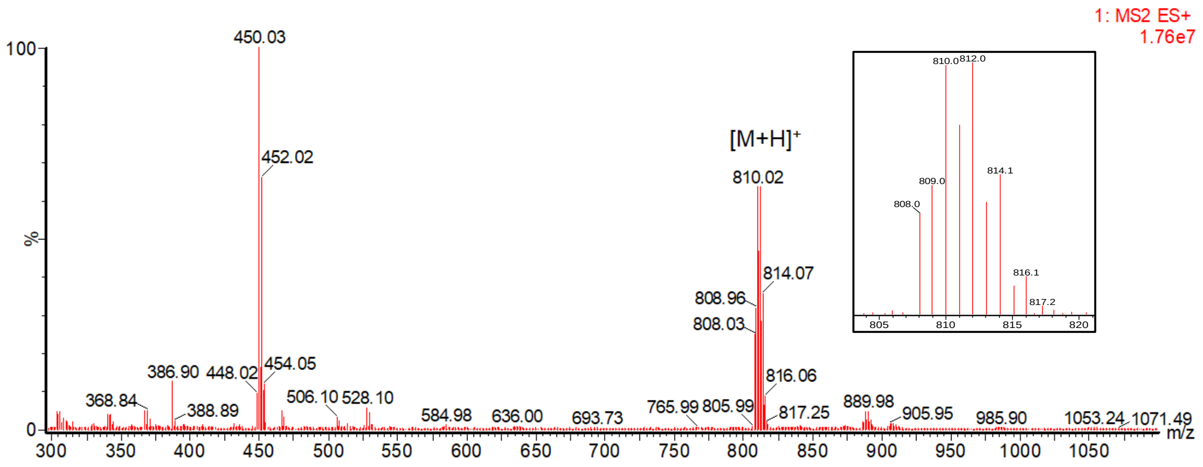

Figure S4. ESI-MS spectrum (+ve mode) of complex **2**.

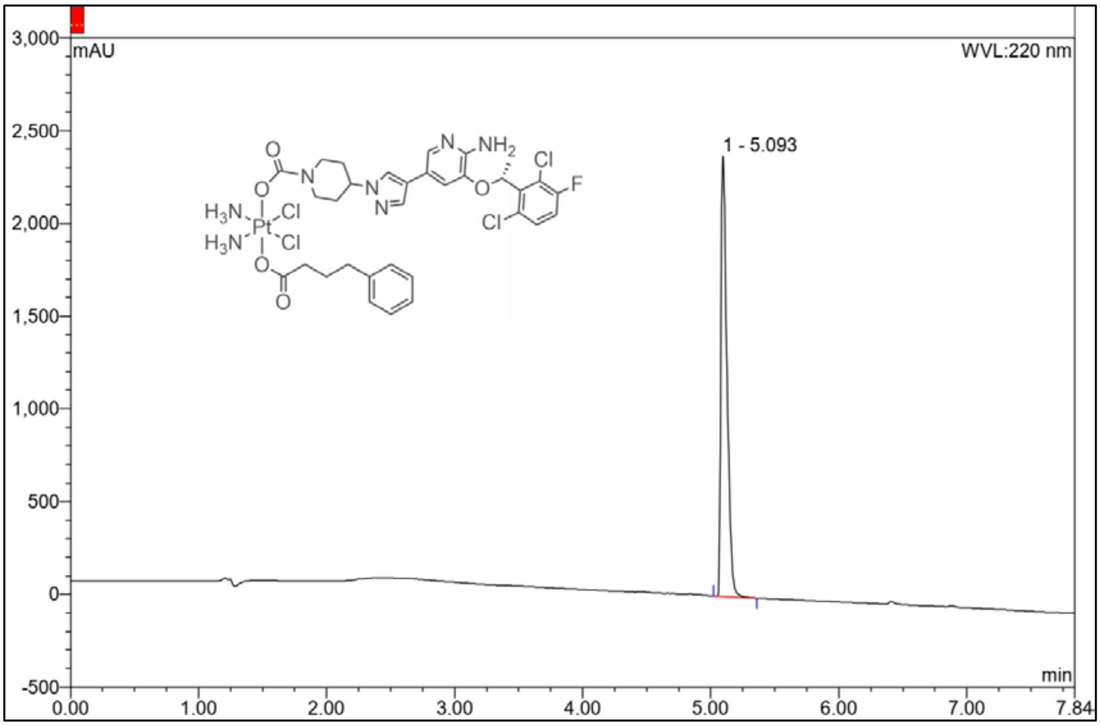

Figure S5. HPLC chromatogram of complex **3** ran with 0 – 100% linear gradient of acetonitrile in 0.1% TFA in water over 5.84 min + 2 min constant 100% acetonitrile

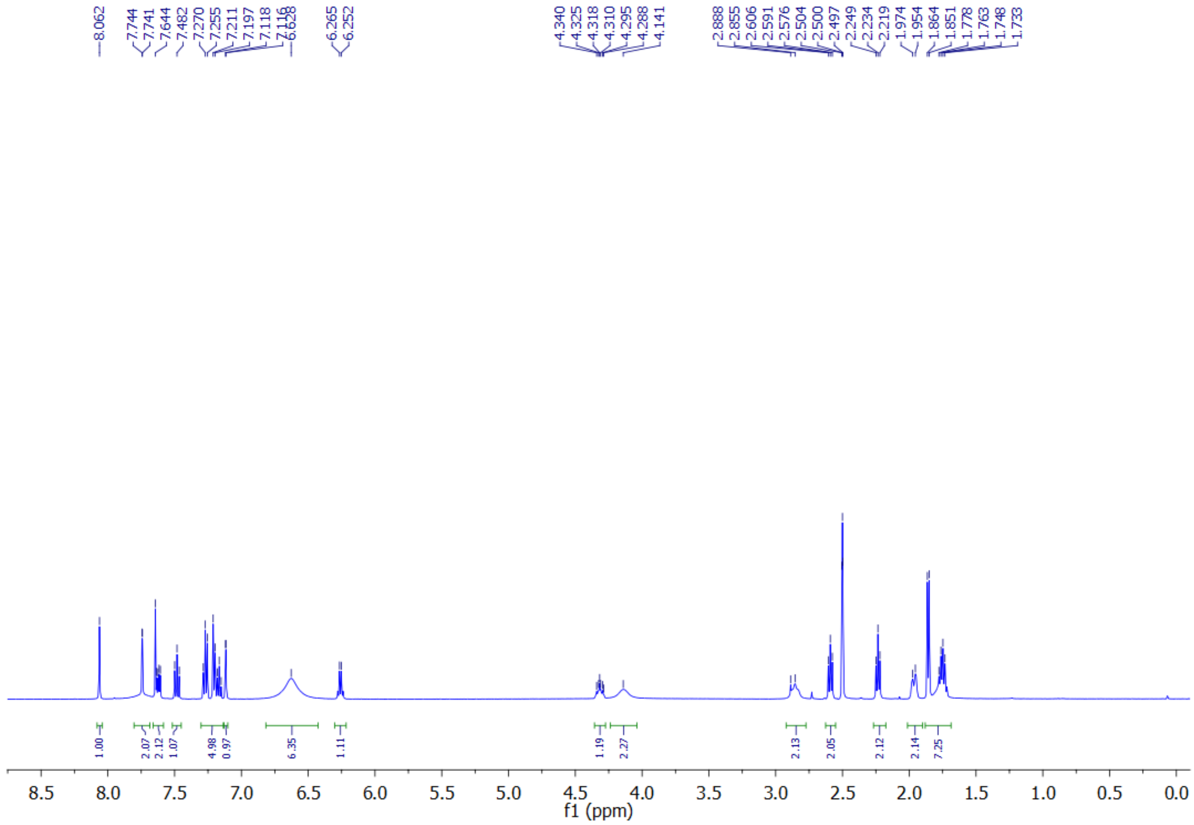

Figure S6. ^{1}H NMR of *ctc*-[Pt(NH_3)$_2$(crizotinib)(PhB)Cl_2] (**3**) in DMSO-d_6.

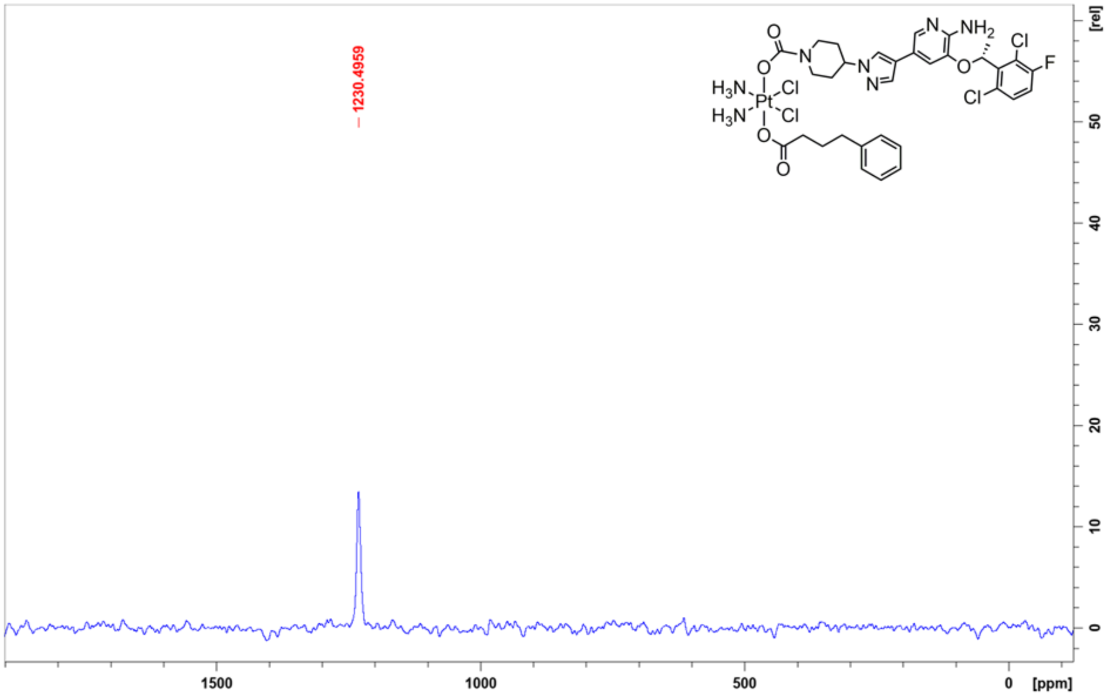

Figure S7. ^{195}Pt NMR of *ctc*-[Pt(NH_3)$_2$(crizotinib)(PhB)Cl_2] (**3**) in DMSO-d6.

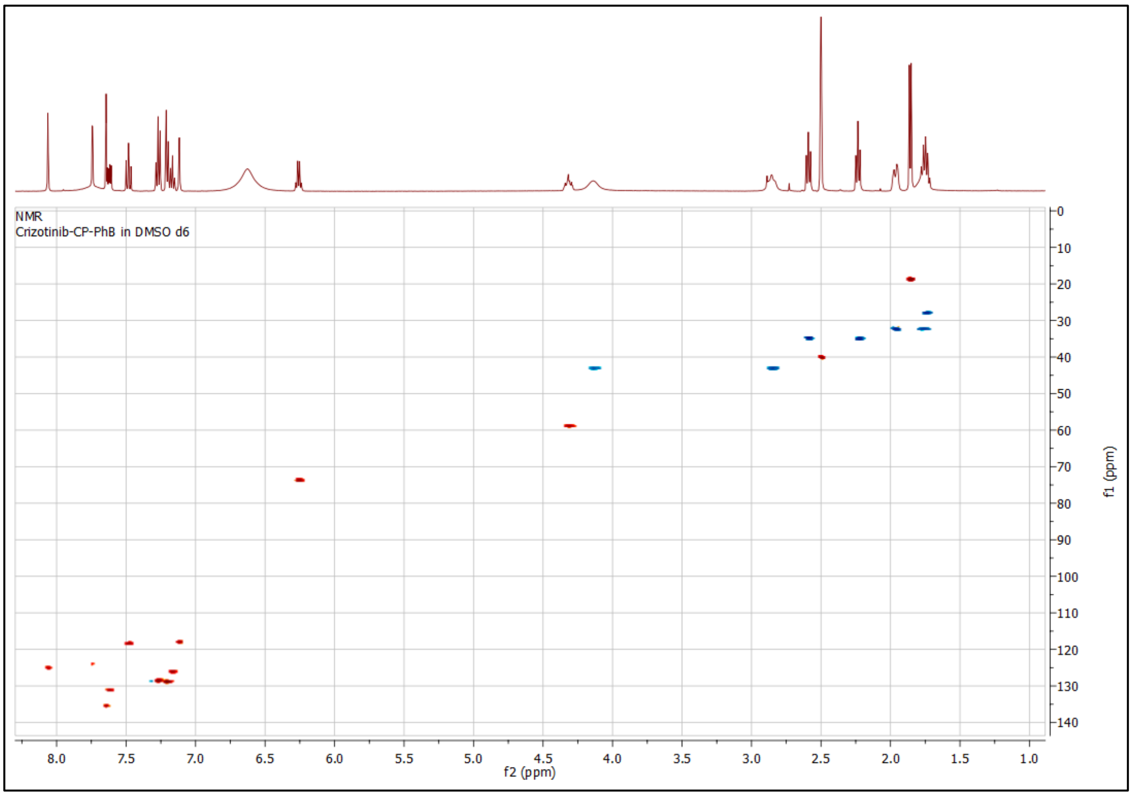

Figure S8. HSQC of *ctc*-[Pt(NH_3)$_2$(crizotinib)(PhB)Cl_2] (**3**) in DMSO-d_6.

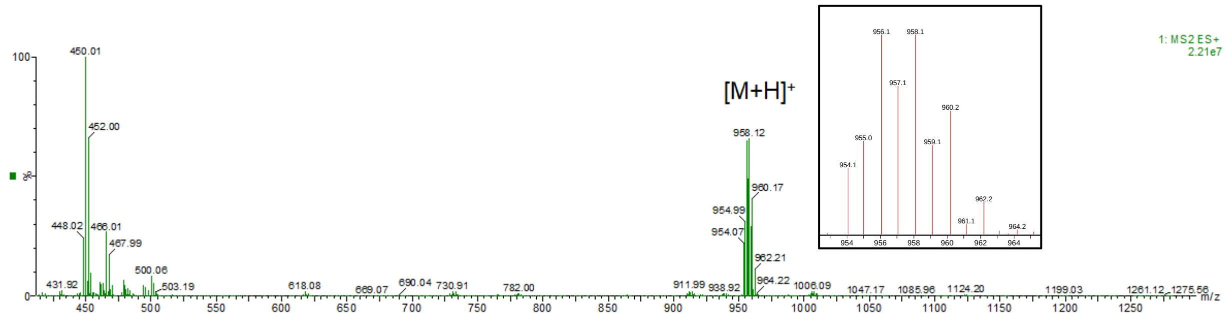

Figure S9. ESI-MS spectrum (+ve mode) of complex **3**.

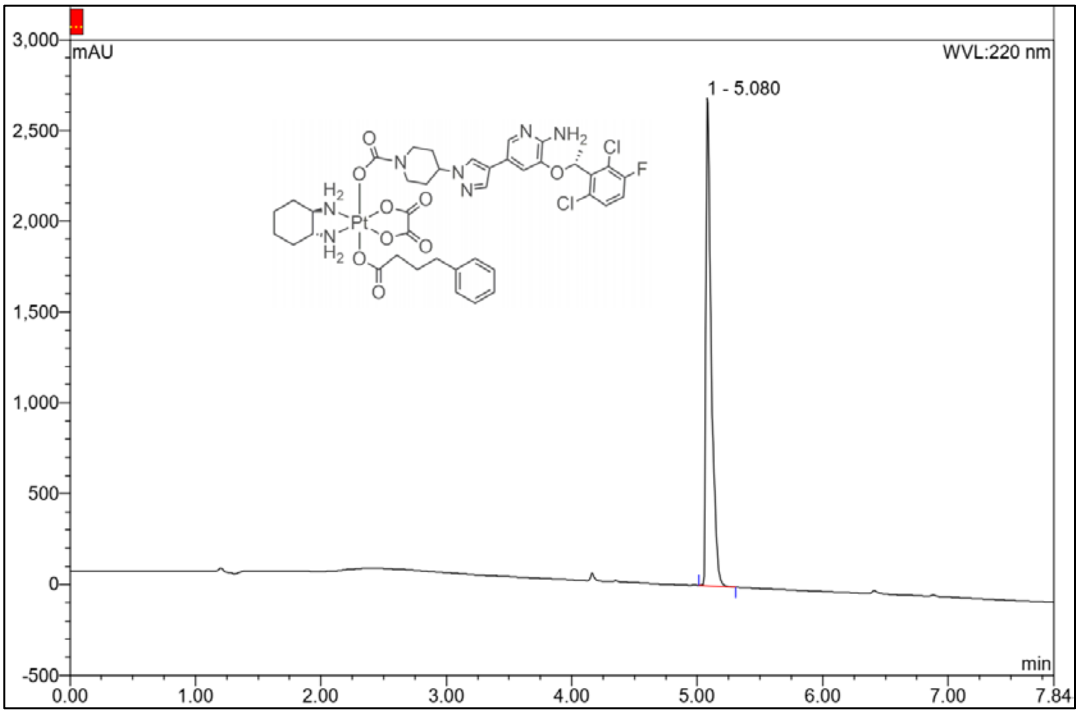

Figure S10. HPLC chromatogram of complex **4** ran with 0 – 100% linear gradient of acetonitrile in 0.1% TFA in water over 5.84 min + 2 min constant 100% acetonitrile.

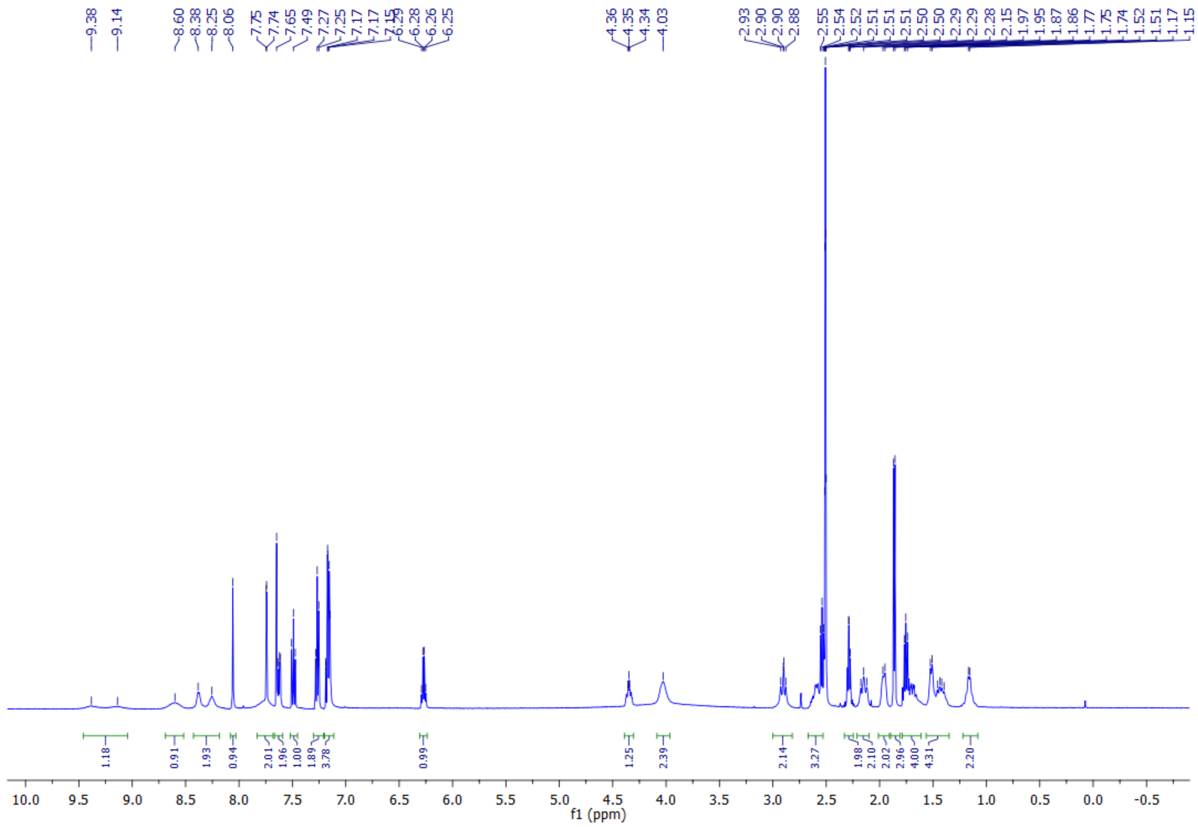

Figure S11. ^{1}H NMR of *ctc*-[Pt(DACH)(crizotinib)(PhB)(Ox)] (**4**) in DMSO-d_6.

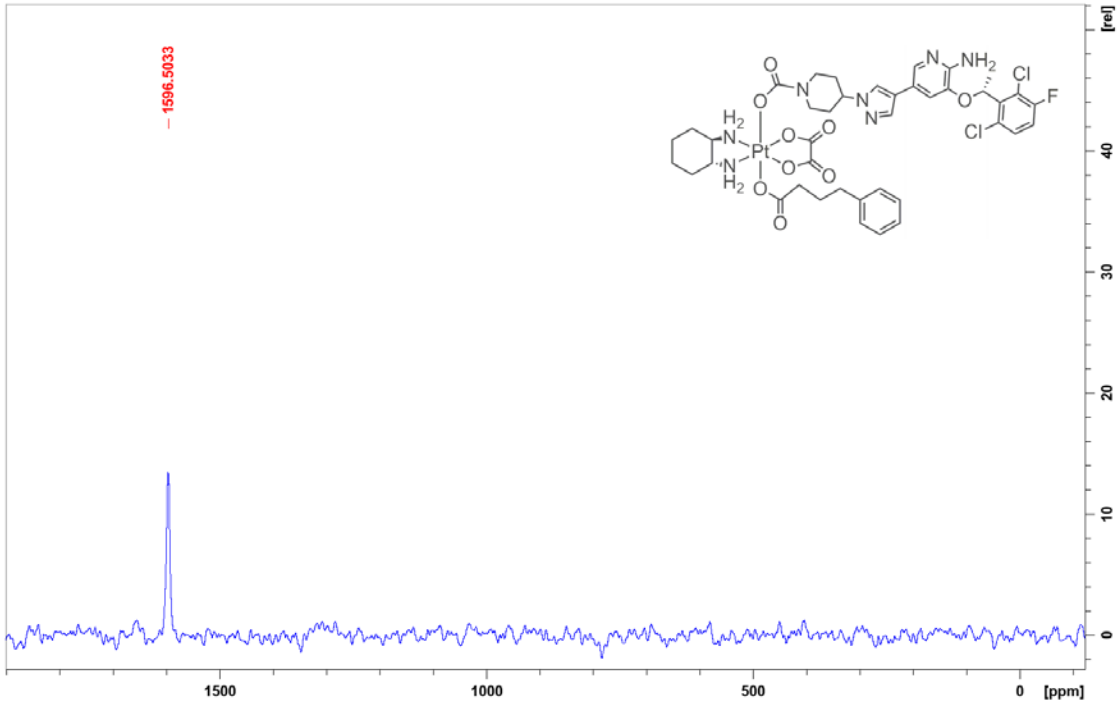

Figure S12. ^{195}Pt NMR of ctc-[Pt(DACH)(crizotinib)(PhB)(Ox)] (**4**) in DMSO-d_6.

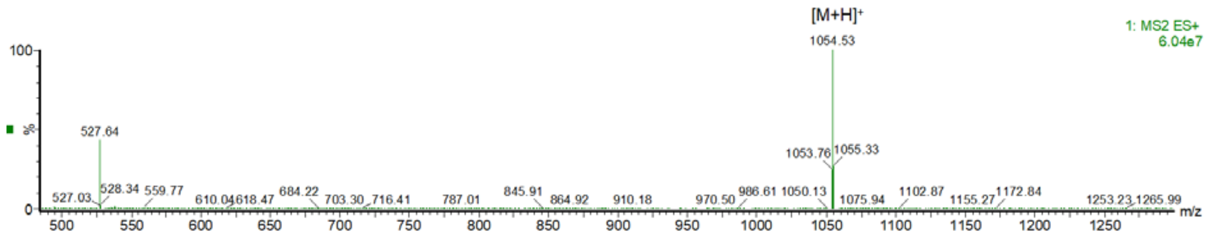

Figure S13. ESI-MS spectrum (+ve mode) of complex **4**.

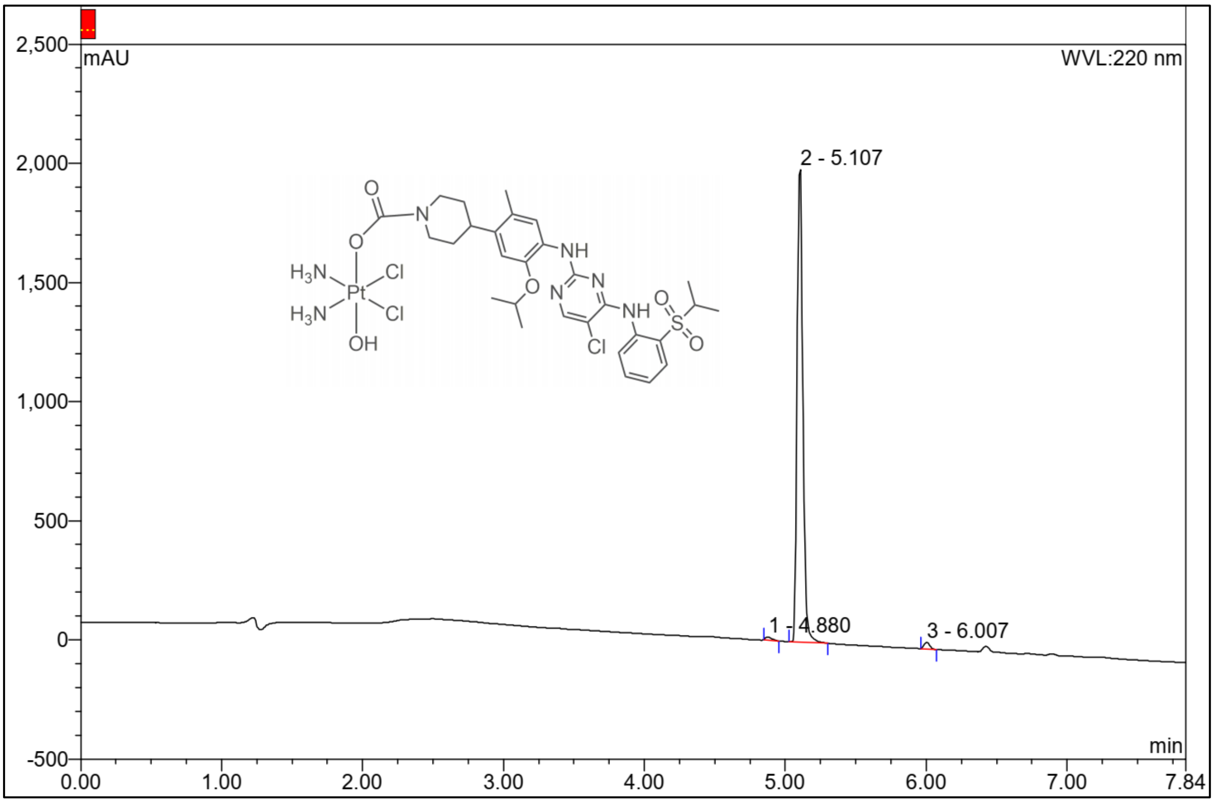

Figure S14. HPLC chromatogram of complex **6** ran with 0 – 100% linear gradient of acetonitrile in 0.1% TFA in water over 5.84 min + 2 min constant 100% acetonitrile

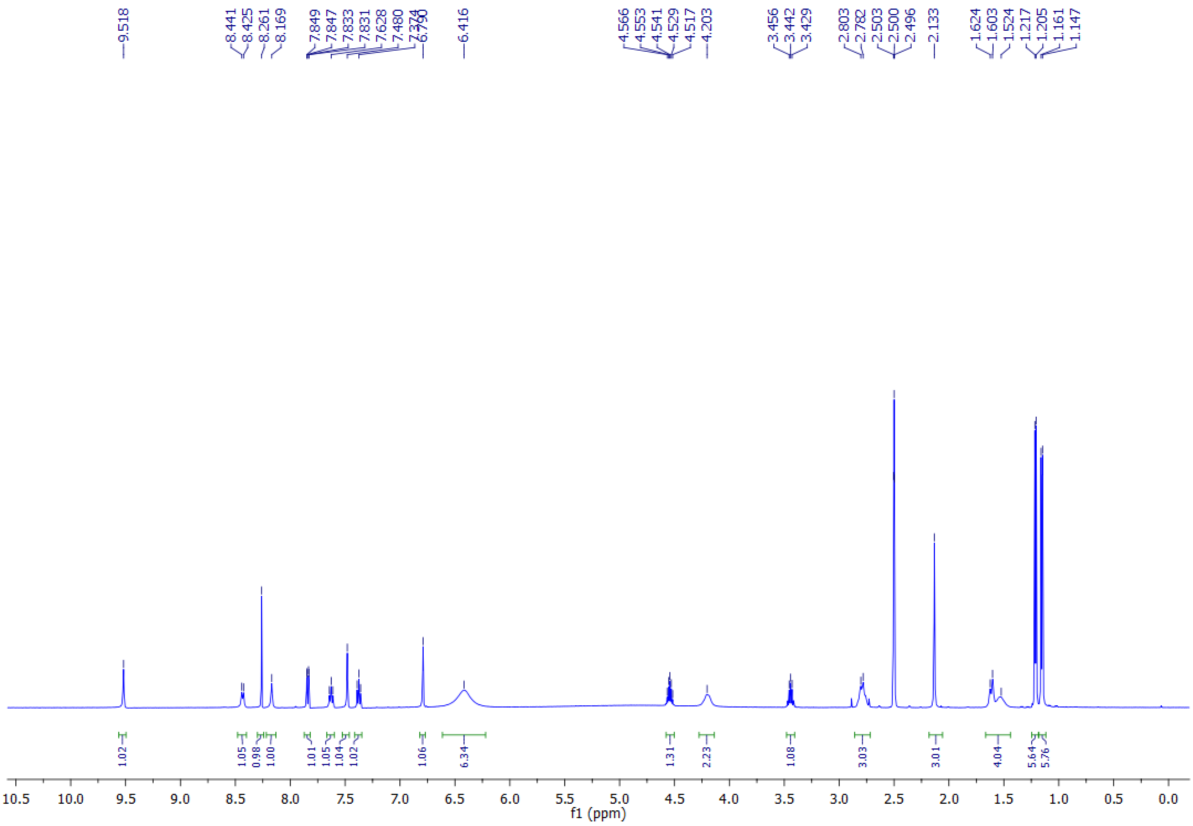

Figure S15. ^{1}H NMR of ctc-[Pt(NH_3)$_2$(ceritinib)(OH)Cl_2] (**6**) in DMSO-d6.

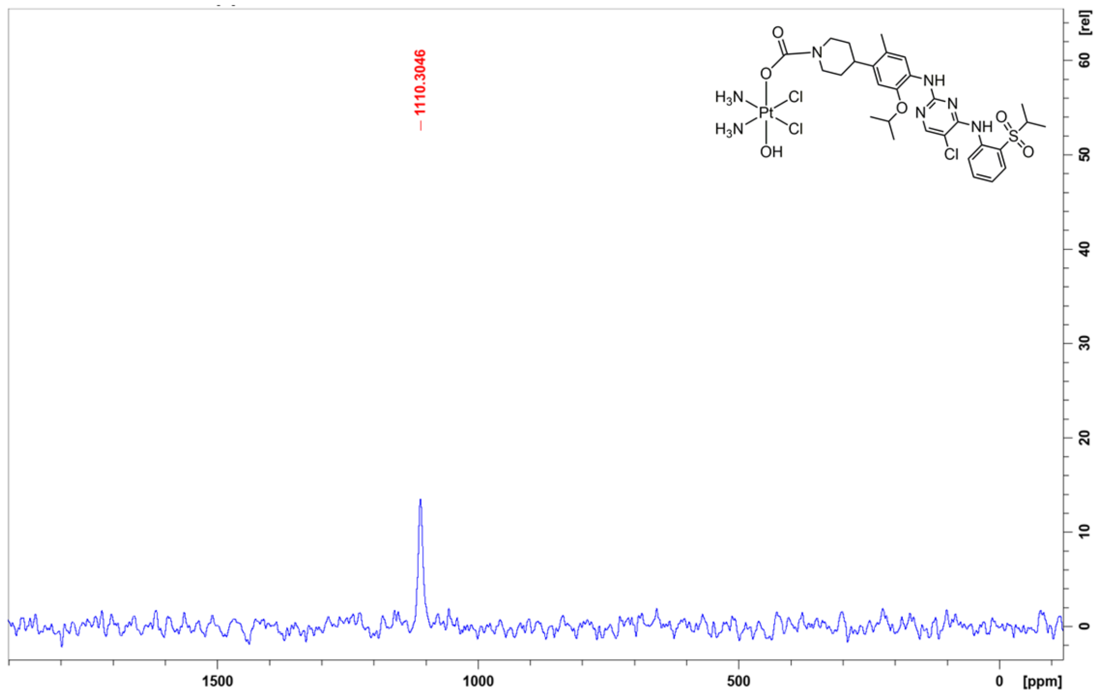

Figure S16. ^{195}Pt NMR of ctc-[Pt(NH_3)$_2$(ceritinib)(OH)Cl_2] (**6**) in DMSO-d6.

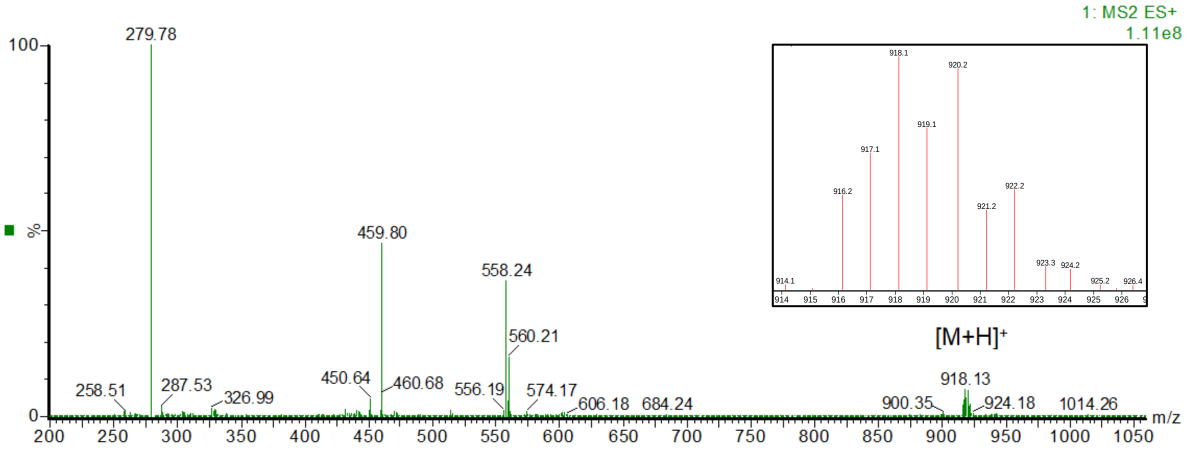

Figure S17. ESI-MS spectrum (+ve mode) of complex **6**.

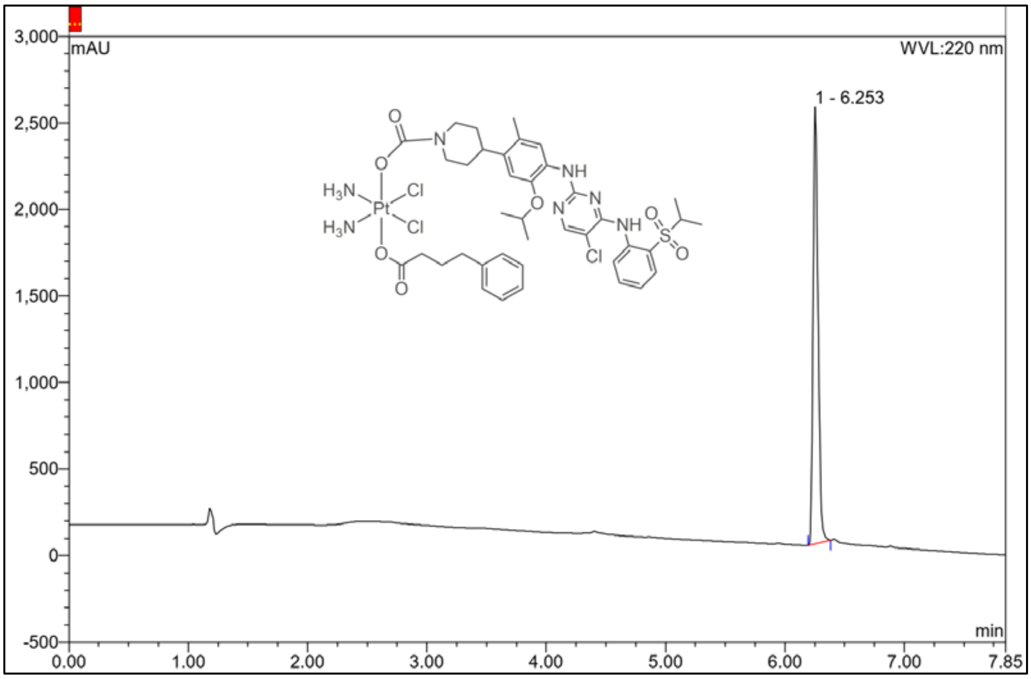

Figure S18. HPLC chromatogram of complex **7** ran with 0 – 100% linear gradient of acetonitrile in 0.1% TFA in water over 5.84 min + 2 min constant 100% acetonitrile

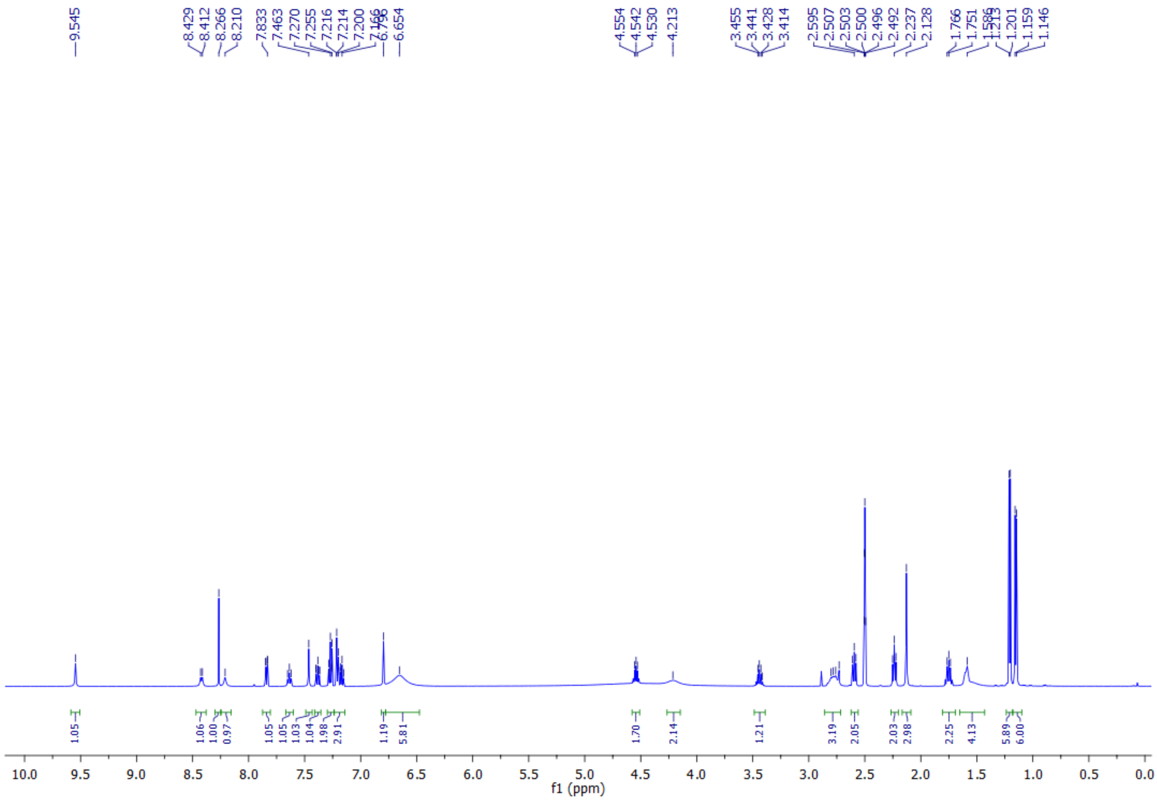

Figure S19. ^{1}H NMR of ctc-[Pt(NH$_3$)$_2$(ceritinib)(PhB)Cl$_2$] (**7**) in DMSO-d$_6$.

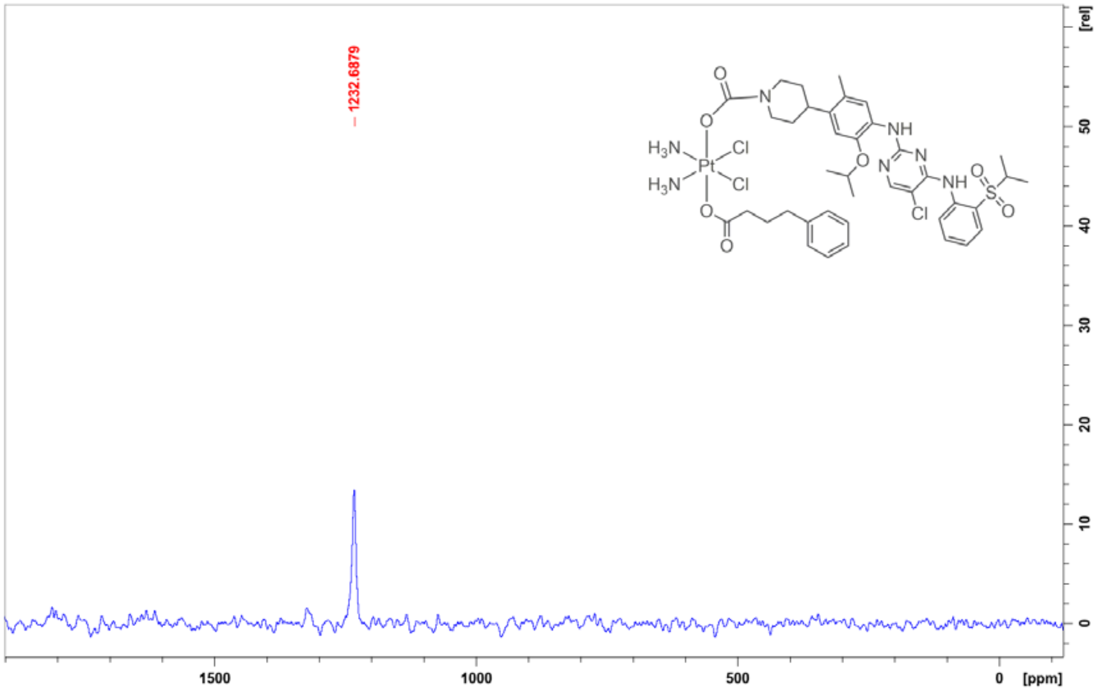

Figure S20. ^{195}Pt NMR of ctc-[Pt(NH$_3$)$_2$(ceritinib)(PhB)Cl$_2$] (**7**) in DMSO-d6.

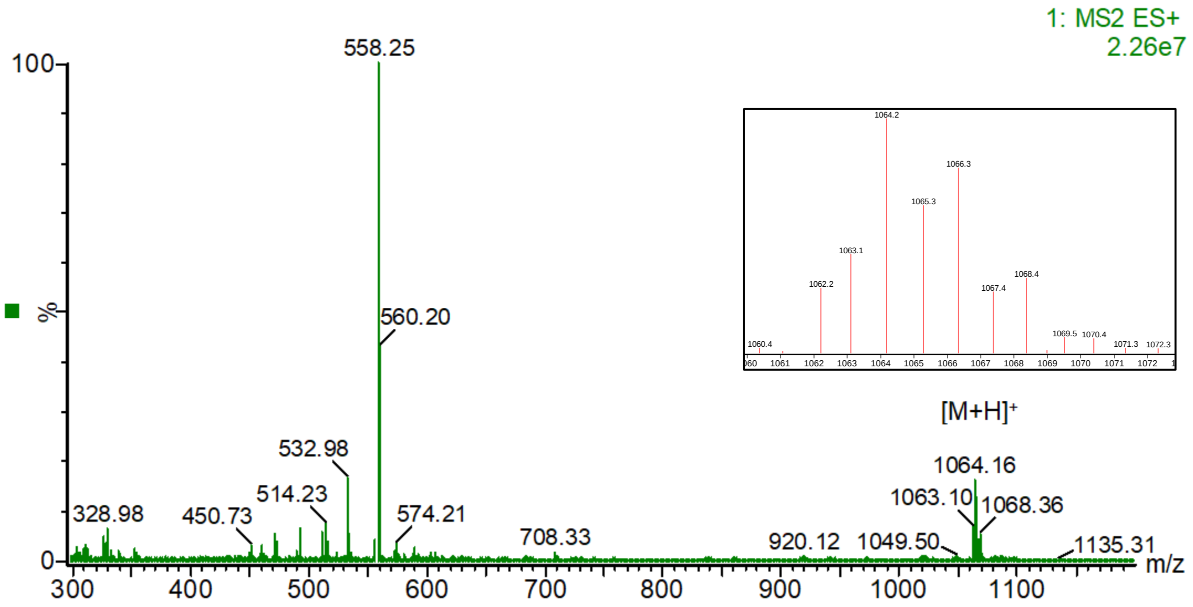

Figure S21. ESI-MS spectrum (+ve mode) of complex **7**.

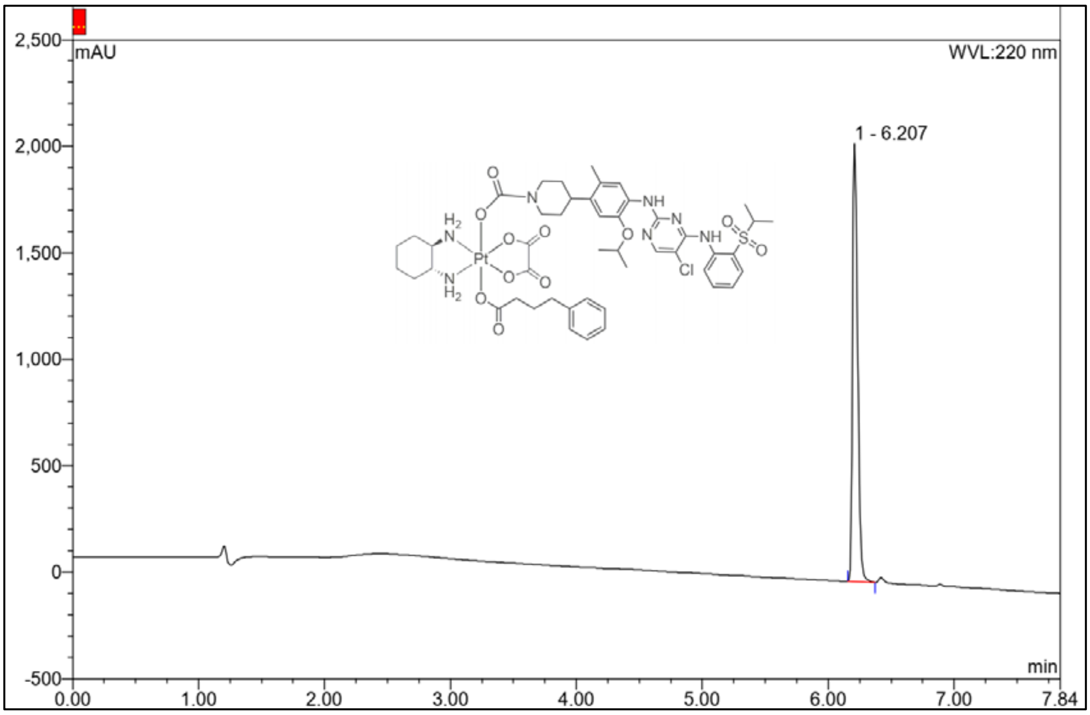

Figure S22. HPLC chromatogram of complex **8** ran with 0 – 100% linear gradient of acetonitrile in 0.1% TFA in water over 5.84 min + 2 min constant 100% acetonitrile

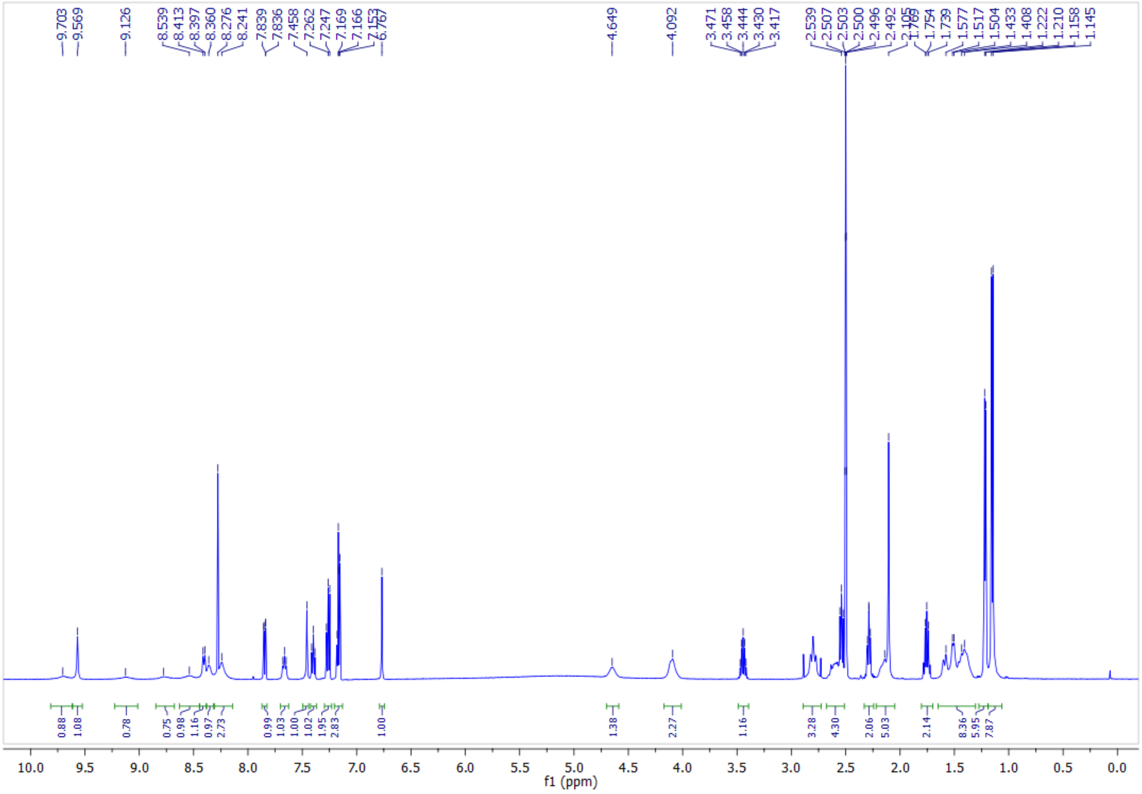

Figure S23. ^{1}H NMR of ctc-[Pt(DACH)(ceritinib)(PhB)(Ox)] (**8**) in DMSO-d6.

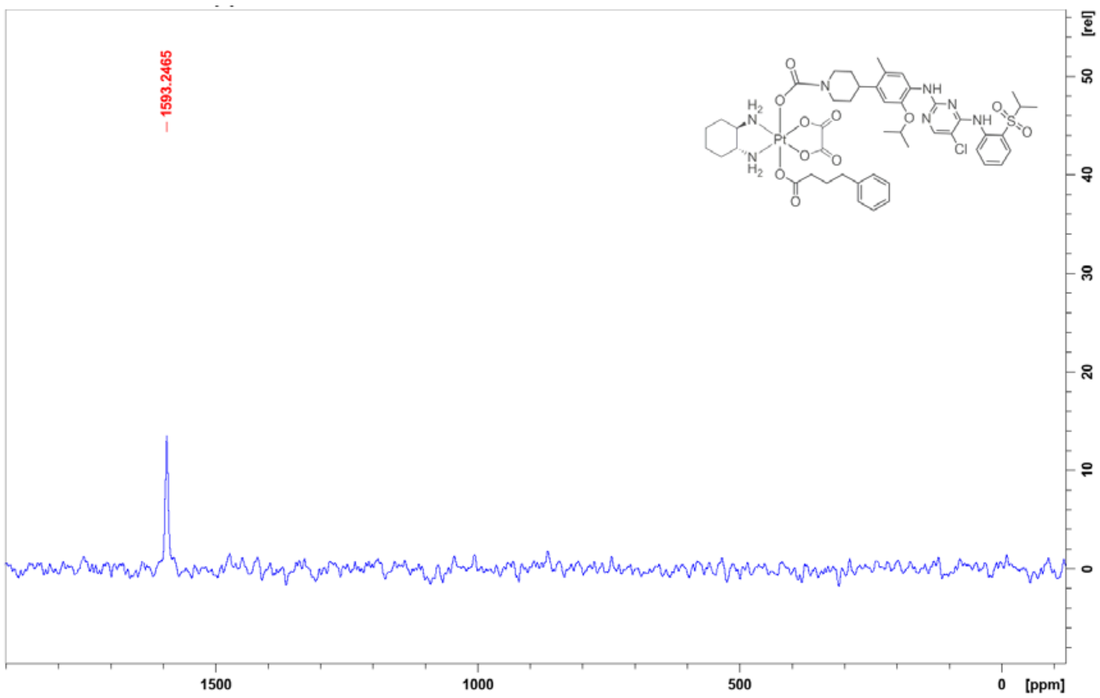

Figure S24. ^{195}Pt NMR of ctc-[Pt(DACH)(ceritinib)(PhB)(Ox)] (**8**) in DMSO-d6.

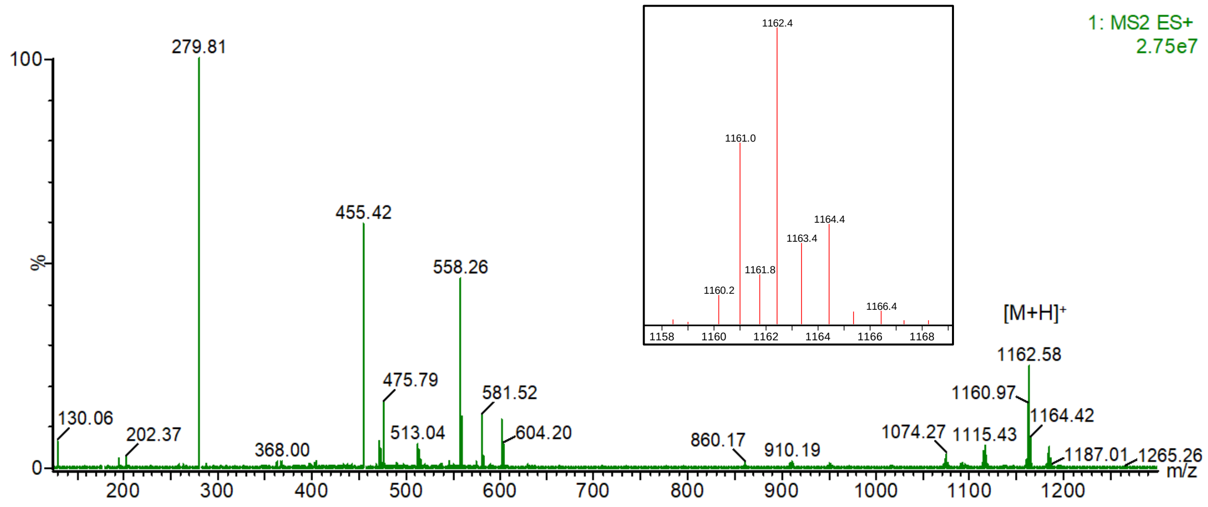

Figure S25. ESI-MS spectrum (+ve mode) of complex **8**.

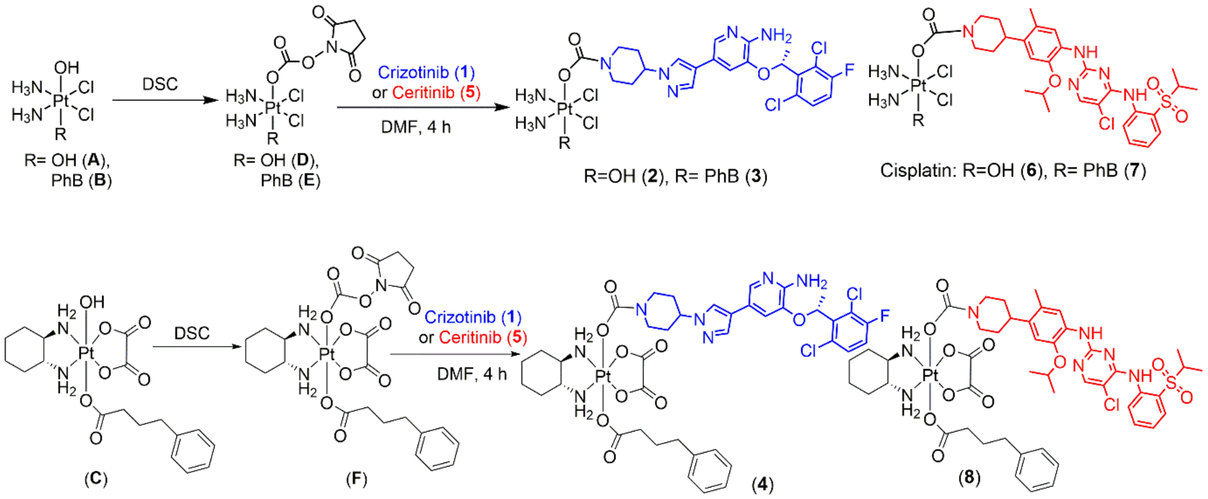

Scheme S1. Generalized synthetic approach to the preparation of complexes Pt(IV)-crizotinib (**2-4**) and Pt(IV)-ceritinib complexes (**6-8**) .

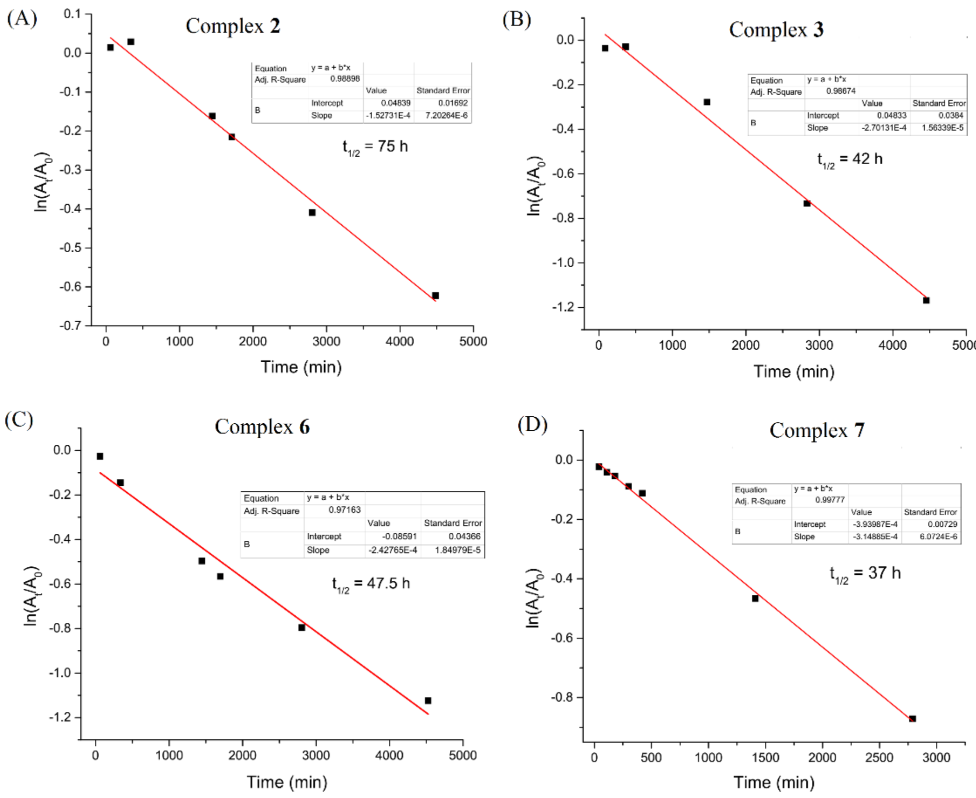

Figure S26. Stability half-lives of complexes (A) **2**, (B) **3**, (C) **6** and (D) **7** in 10 % DMSO in RPMI media at 37 °C determined by HPLC.

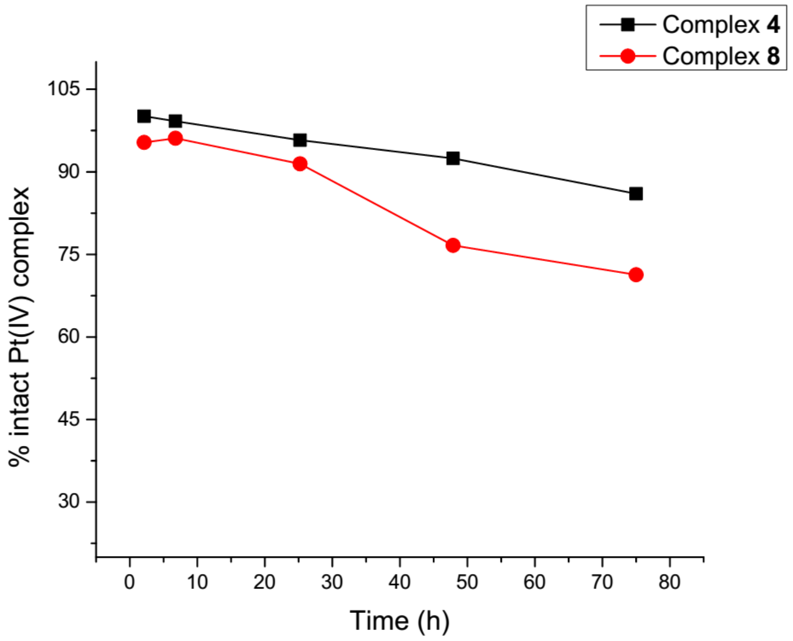

Figure S27. Percentage of intact complexes **4** and **8** in different time intervals in 10 % DMSO in RPMI media at 37 °C.

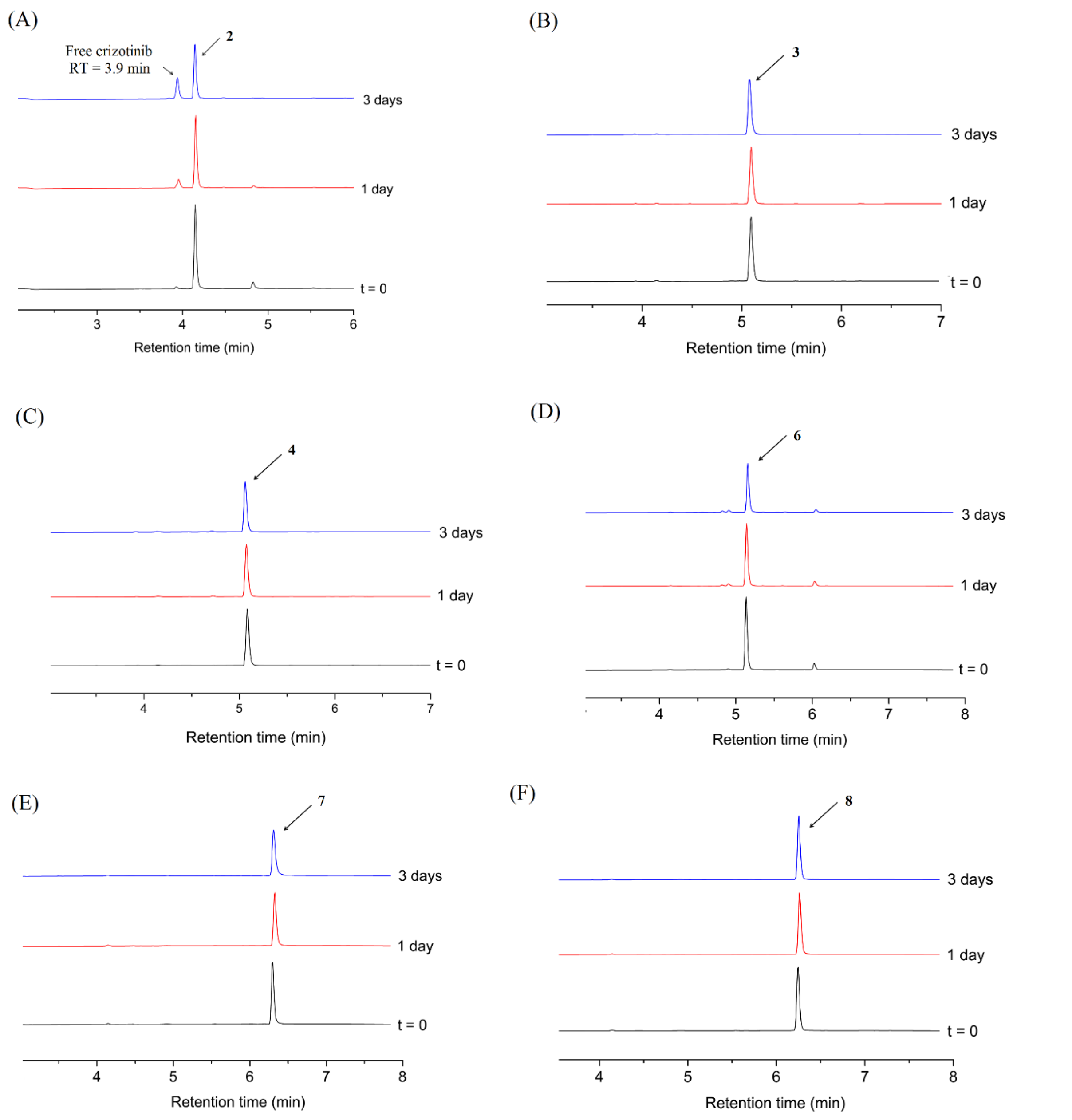

Figure S28. Stability plot of the complexes at different time intervals in 10 % DMSO in PBS at 37 °C monitored by HPLC.

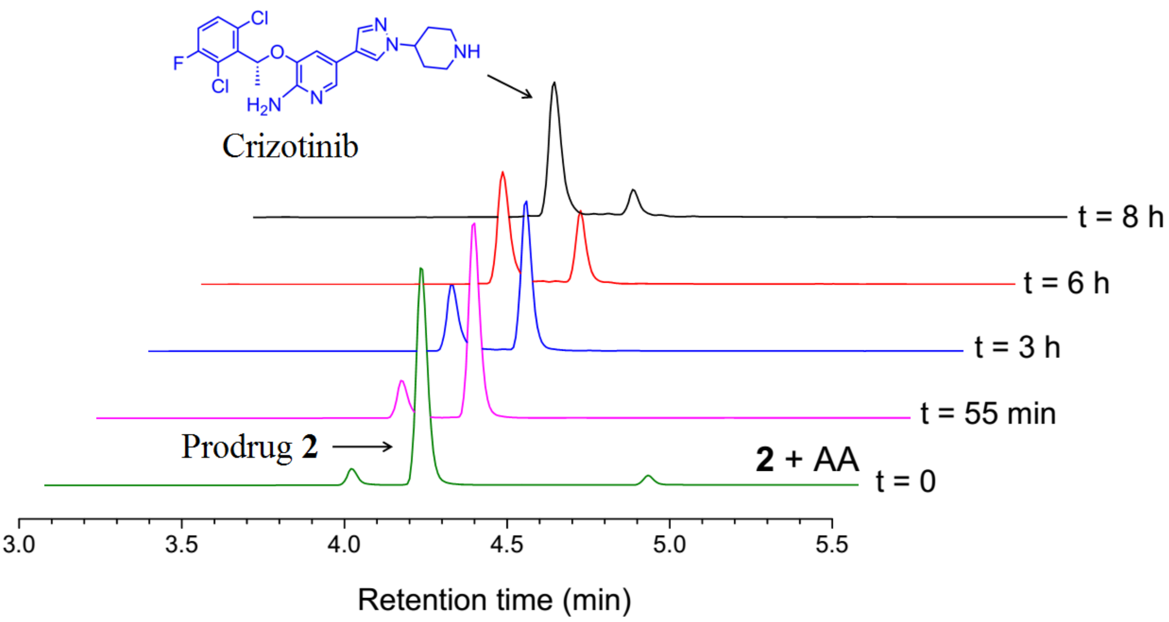

Figure S29. Reduction of **2** in the presence of 10 equiv. ascorbic acid taken at different time intervals in 100 mM phosphate buffer at pH 7.4 at 37 ºC

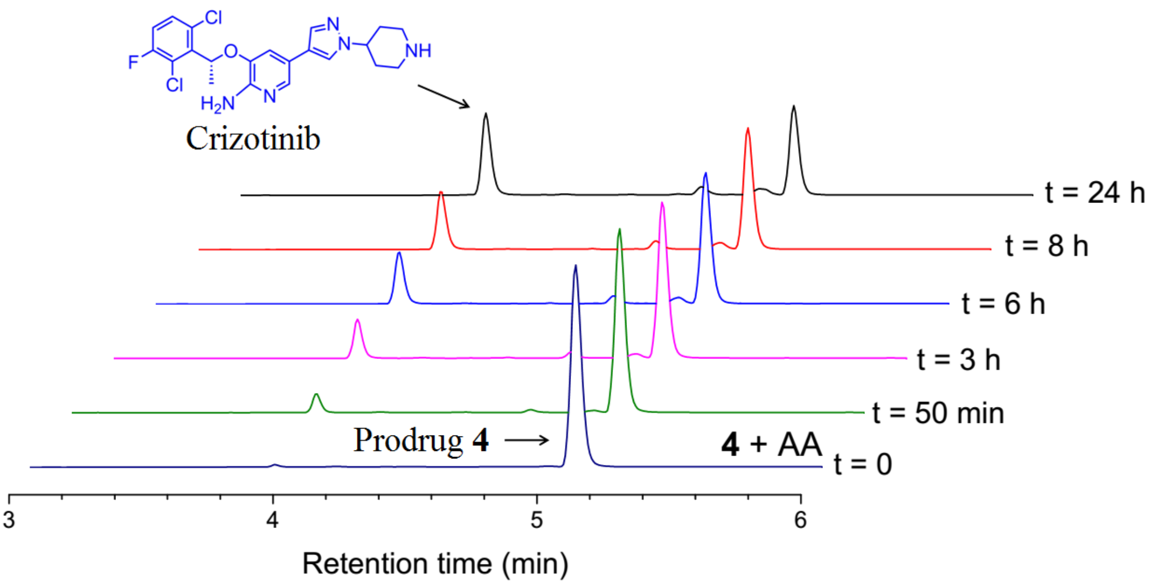

Figure S30. Reduction of **4** in the presence of 10 equiv. ascorbic acid taken at different time intervals in 100 mM phosphate buffer at pH 7.4 at 37 ºC.

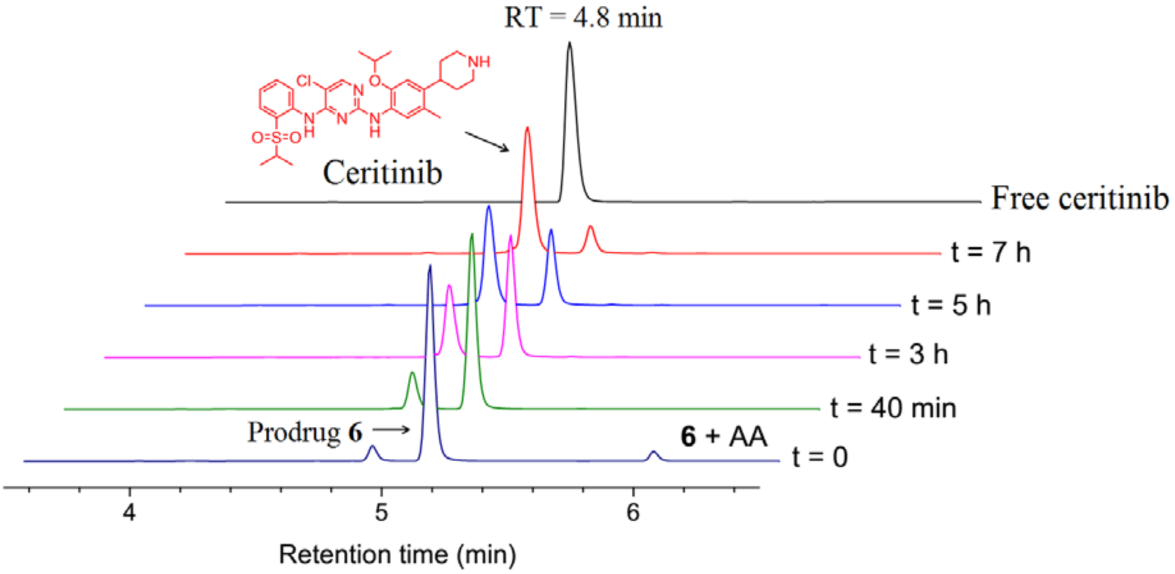

Figure S31. Reduction of **6** in the presence of 10 equiv. ascorbic acid taken at different time intervals in 100 mM phosphate buffer at pH 7.4 at 37 ºC

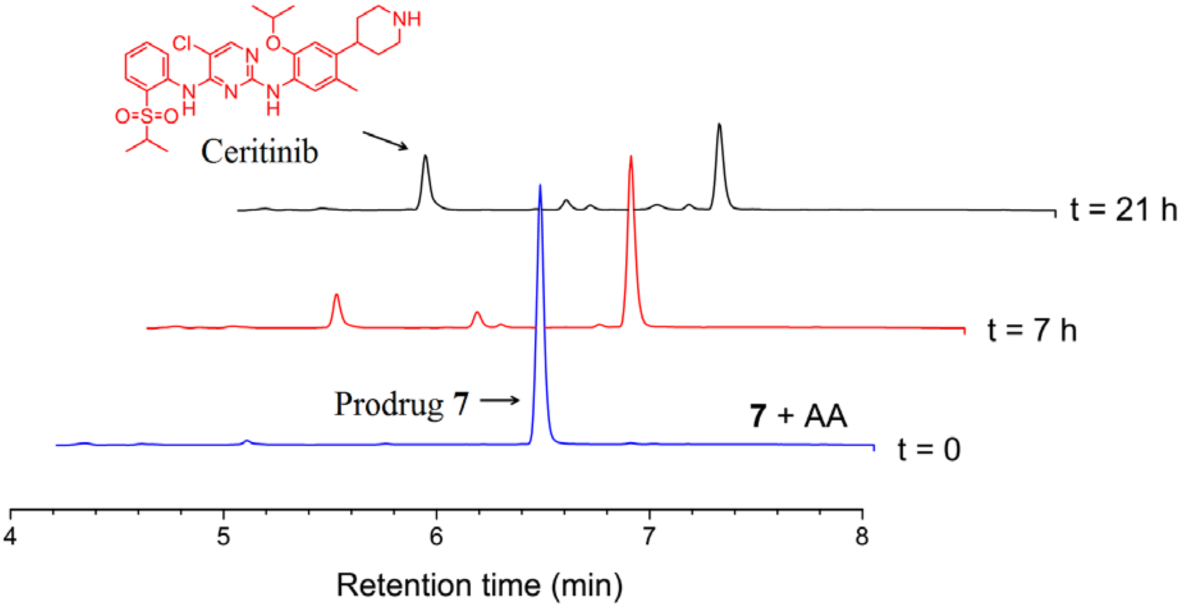

Figure S32. Reduction of **7** in the presence of 10 equiv. ascorbic acid taken at different time intervals in 100 mM phosphate buffer at pH 7.4 at 37 ºC

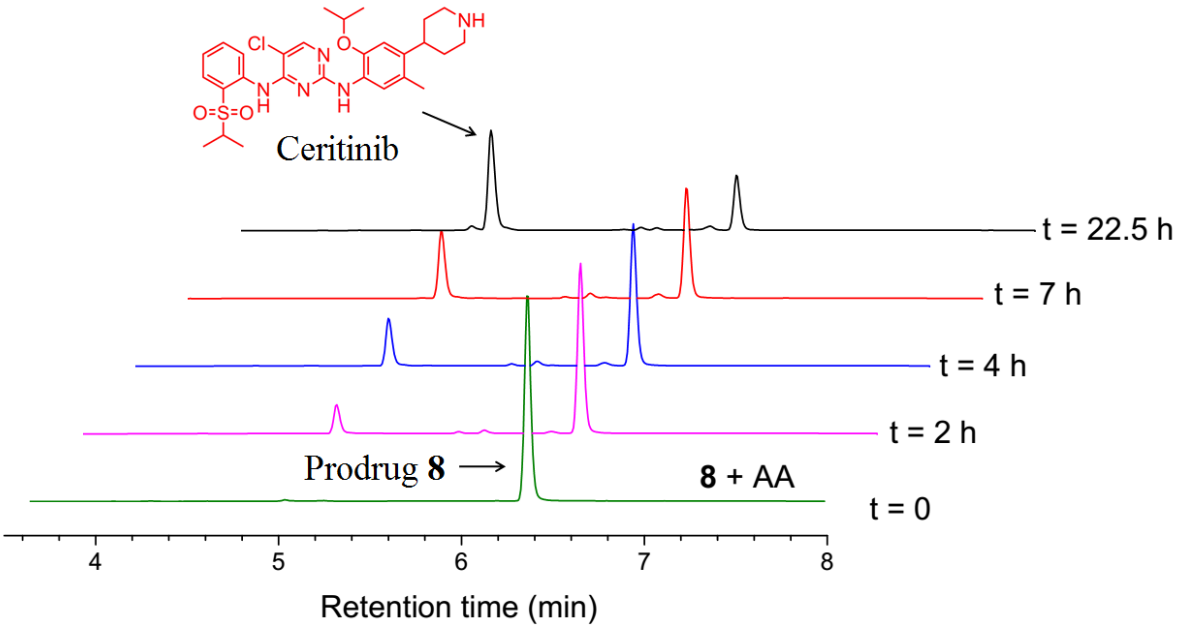

Figure S33. Reduction of **8** in the presence of 10 equiv. ascorbic acid taken at different time intervals in 100 mM phosphate buffer at pH 7.4 at 37 ºC.

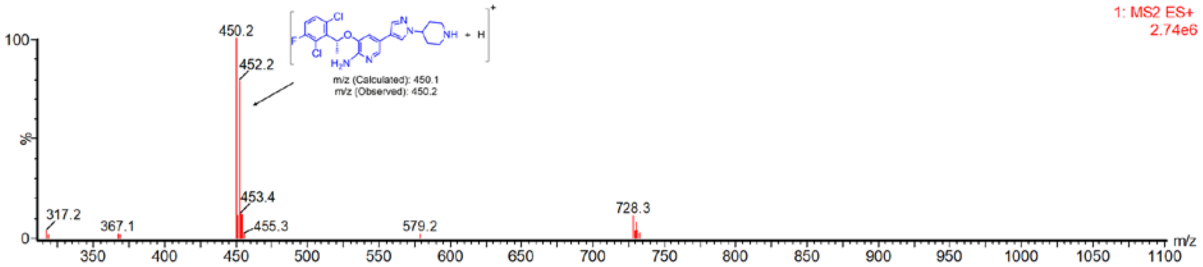

Figure S34. ESI-MS data for the reduction of **2** in the presence of 10 equiv. ascorbic acid in 100 mM phosphate buffer at pH 7.4 at 37 ºC after overnight incubation.

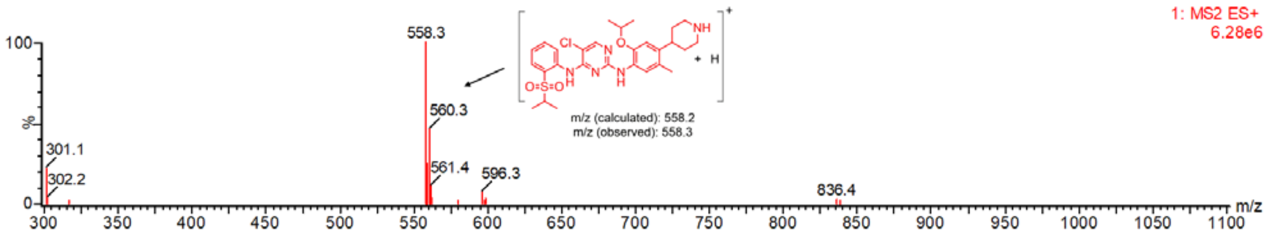

Figure S35. ESI-MS data for the reduction of **6** in the presence of 10 equiv. ascorbic acid in 100 mM phosphate buffer at pH 7.4 at 37 °C after overnight incubation.

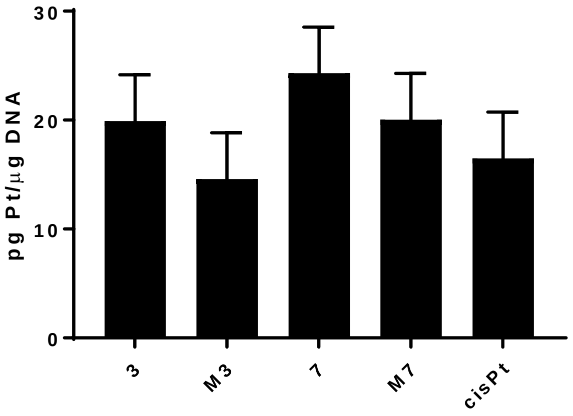

Figure S36. DNA platination. NCI-H2228 cells were exposed to 5 µM complexes for 4 h. Cell pellets were lysed, and DNA was isolated with DNAzol. DNA concentration was determined spectrophotometrically, and platinum content was determined with ICP-MS. The results are shown as MEAN±SD from two experiments.

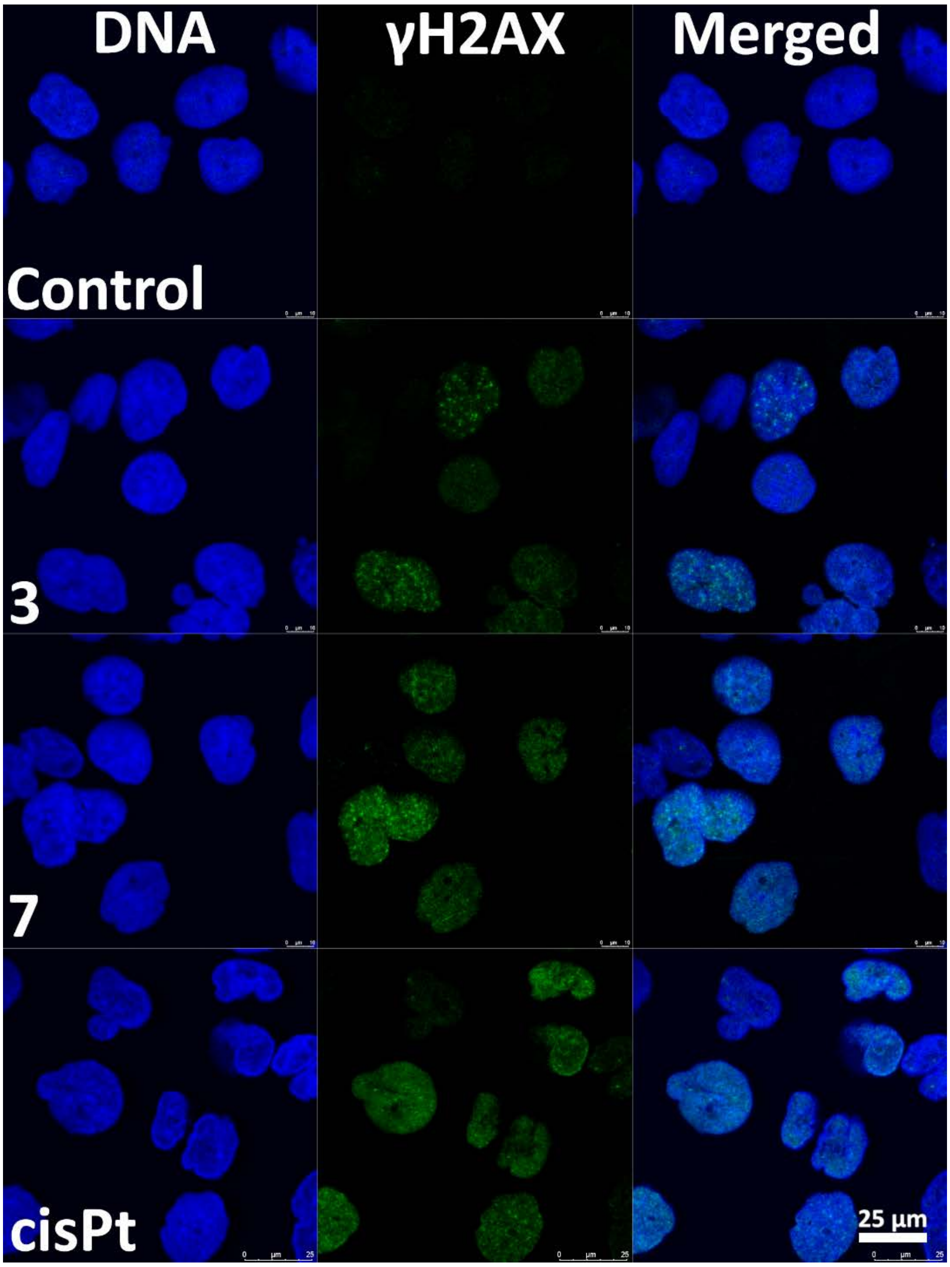

Figure S37. DNA damage. H2AX phosphorylation (γH2AX foci) was used as the indicator of DNA damage in samples exposed to **3**, **7,** and cisplatin. NCI-H2228 cells were nontreated or treated with the tested compounds at concentrations corresponding to their respective 3xIC_{50} values for 24 hours. The cells were stained with an anti-γH2AX antibody and secondary AlexaFluor 488 conjugated antibody and counter-stained with DAPI. The images were recorded with a confocal microscope.

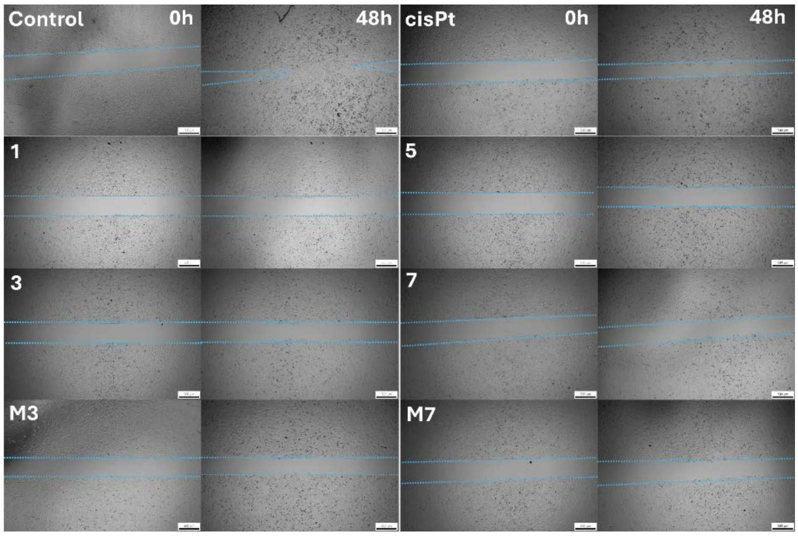

Figure S38. Scratch test. A549 cells were grown in 24-well plates with insert chambers (Ibidi). The inserts were then removed, and fresh medium containing the complexes at concentrations corresponding to IC_{50} values was added. The images were recorded immediately after the addition and then after 48 h. Representative images.

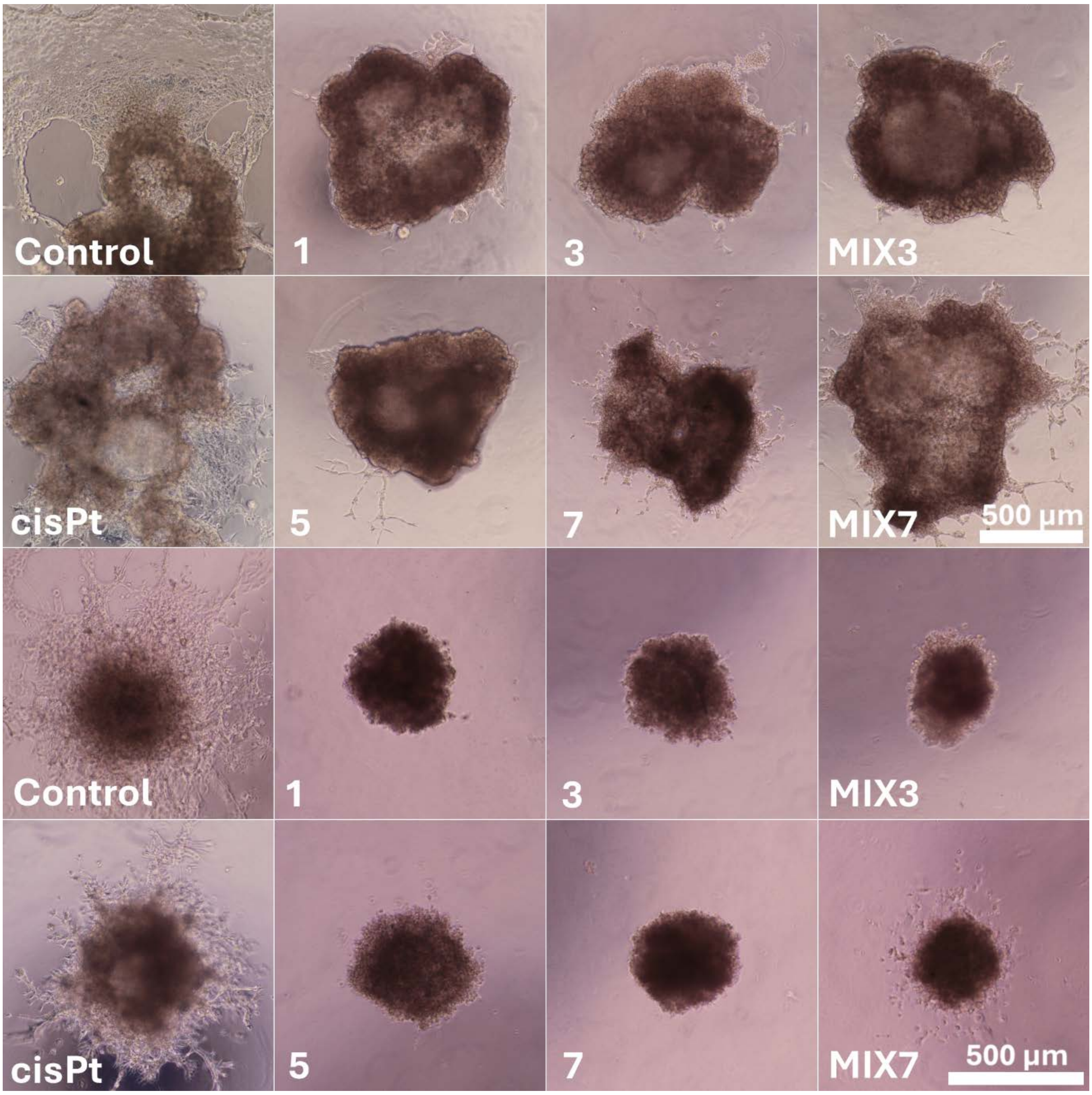

Figure S39. Spheroid outgrowing into the Matrigel. Top two rows - A549-derived spheroids. Bottom two rows – NCI-H2228 derived spheroids. The spheroids were grown for four days, then embedded in Matrigel and treated with the respective compounds at concentrations corresponding to their IC_{50} values. The images were recorded after 72 h of treatment. Representative images.

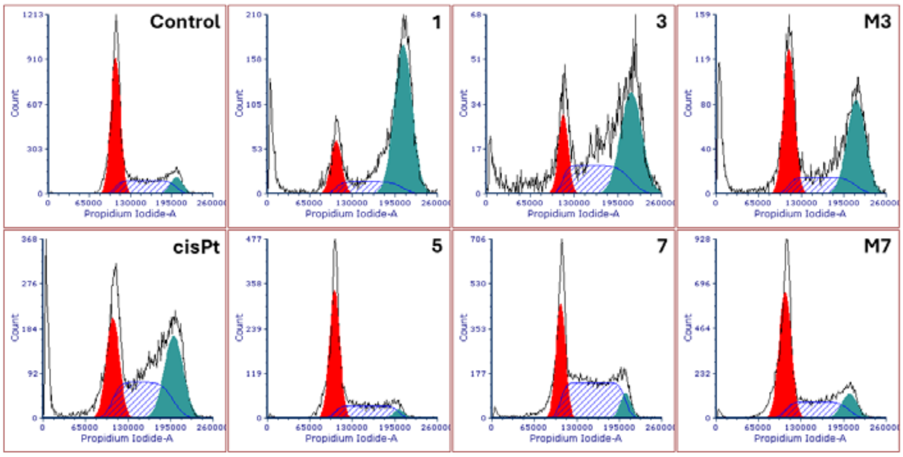

Figure S40. Cell cycle. Representative flow cytometry histograms of NCI-H2228 cells exposed to 2xIC_{50} concentrations of the investigated compounds for 48 h. Cell distribution into individual cell cycle phases was performed after staining with propidium iodide. Red – G1; Blue-striped – S; Green – G2/M.

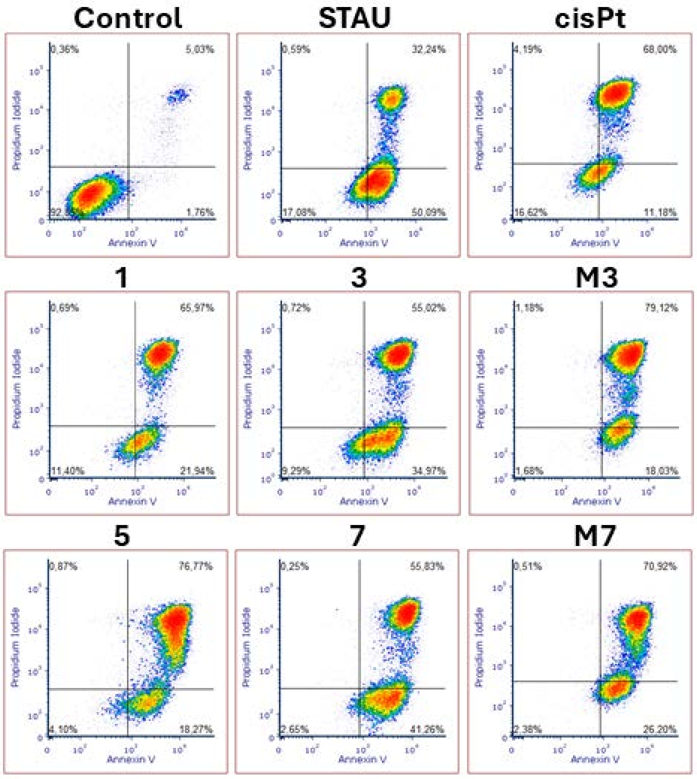

Figure S41. Cell death. NCI-H2228 cells were exposed to the investigated compounds at concentrations corresponding to 3xIC_{50} values for 24 h. The cells were then stained with Annexin V/propidium iodide (PI) and the cell distribution into the quadrants is as follows: left-bottom: Annexin V^+/PI^- (living cells); right-bottom: Annexin V^+/PI^- (early apoptotic cells); left-top: Annexin V^-/PI^+ (early necrotic cells); right-top: Annexin V^+/PI^+ (late apoptotic/necrotic cells). STAU – Staurosporine-treated cells.

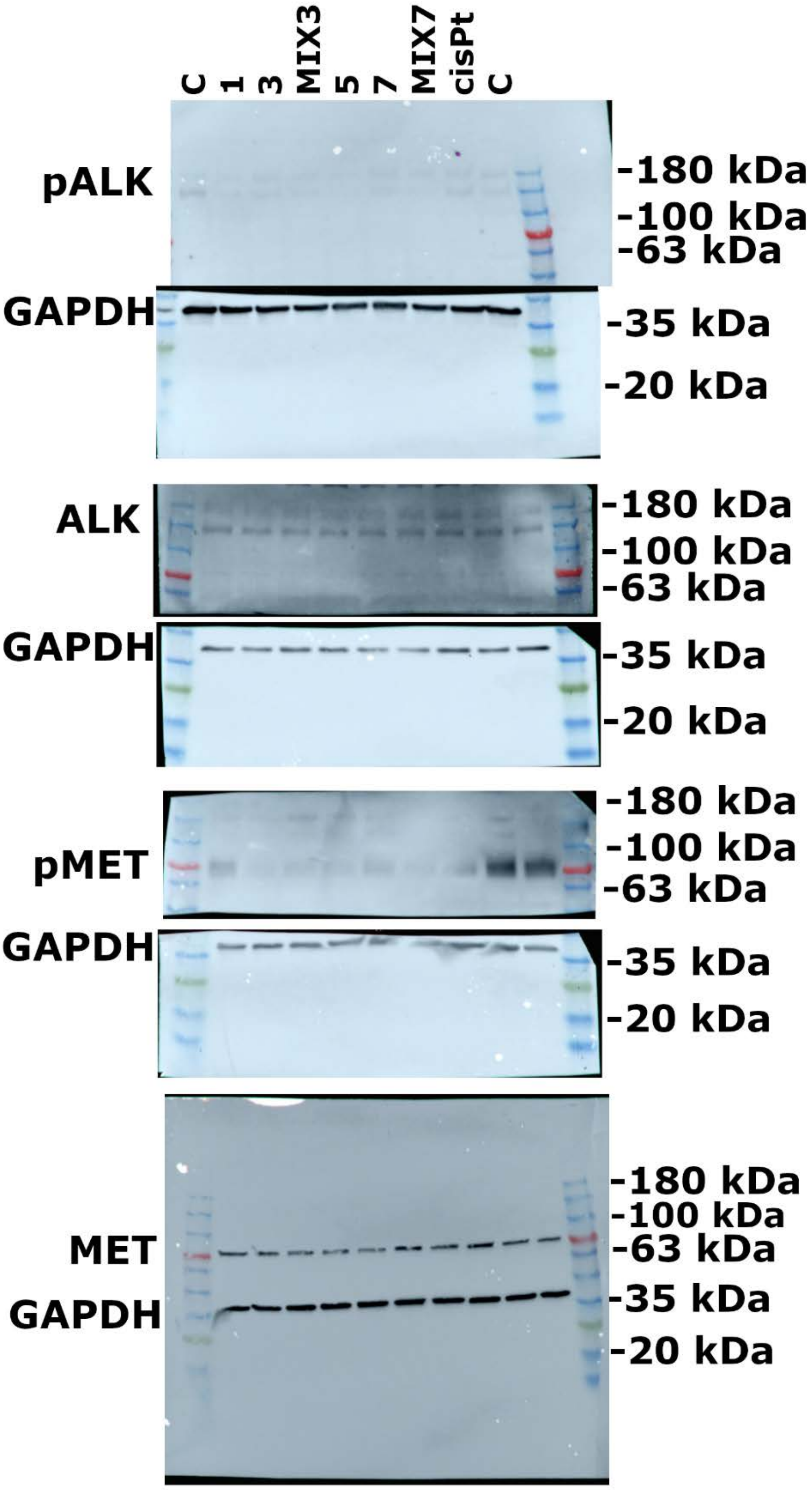

Figure S42. Western blot. Raw images for Figure 7. NCI-H2228 cells were treated with the indicated compounds at concentrations corresponding to their respective IC_{50} values for 24 h.

Table S1. Cytotoxic/antiproliferative activity of selected compounds in CT26 cell line[ab].

(µM)	1	3	MIX3	5	7	MIX7	DOX	cisPt
IC_{50}	1.8±0.4	0.8±0.2	1.5±0.4	1.2±0.3	0.27±0.07	1.1±0.1	2.1±0.3	6±1

[a]Cell viability was assessed with the MTT assay after 72 h of treatment.
[b]MEAN±SD from three independent experiments.

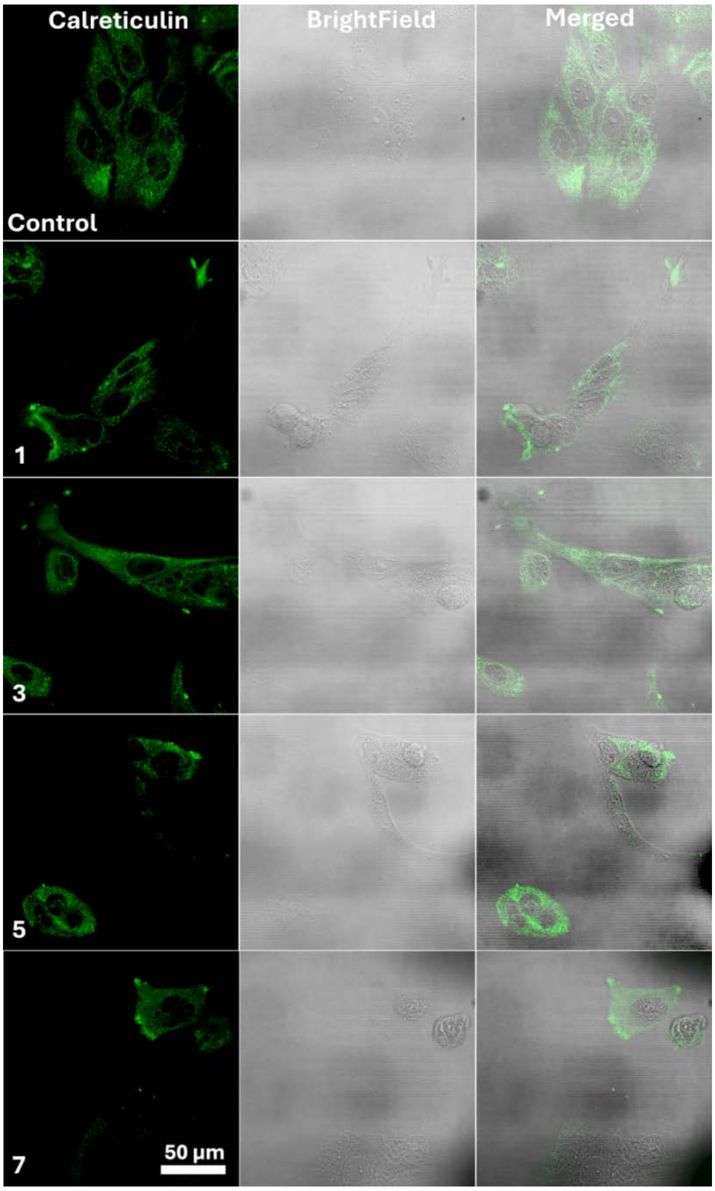

Figure S43. Calreticulin exposure to the cell membrane. NCI-H2228 cells were treated with the compounds at concentrations corresponding to the IC_{50} values for 16 h. Following fixation, the cells were stained with a primary anti-calreticulin antibody and a secondary AlexaFluor 488-conjugated antibody.

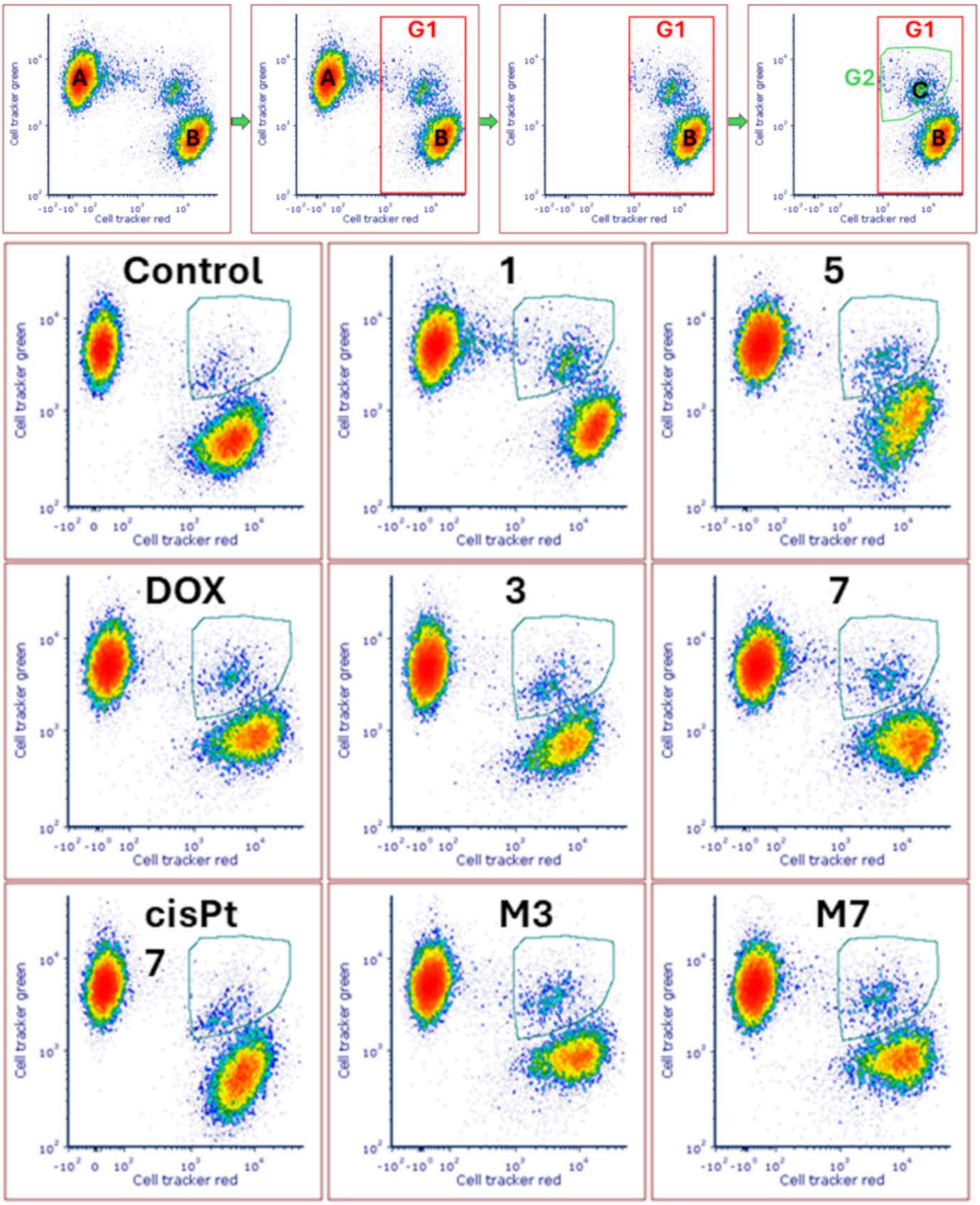

Figure S44. Phagocytosis. CT26 cells were treated with the compounds at concentrations corresponding to IC50 values for 24 h. CT26 samples were then stained with CellTracker red, and J774.A1 macrophages with CellTracker green. The cancer cells were co-incubated with the macrophages for 4 h. The top panel shows the process of evaluating the results. Representative images.
